# Expanding the toolbox of metabolically stable lipid prodrug strategies

**DOI:** 10.3389/fphar.2022.1083284

**Published:** 2023-01-06

**Authors:** Kiran S. Toti, Nicole Pribut, Michael D’Erasmo, Madhuri Dasari, Savita K. Sharma, Perry W. Bartsch, Samantha L. Burton, Hannah B. Gold, Anatoliy Bushnev, Cynthia A. Derdeyn, Adriaan E. Basson, Dennis C. Liotta, Eric J. Miller

**Affiliations:** ^1^ Department of Chemistry, College of Arts and Sciences, Emory University, Atlanta, GA, United States; ^2^ Emory National Primate Research Center, Emory University, Atlanta, GA, United States; ^3^ Emory Vaccine Center, Emory University, Atlanta, GA, United States; ^4^ Department of Pathology and Laboratory Medicine, School of Medicine, Emory University, Atlanta, GA, United States; ^5^ Department of Laboratory Medicine and Pathology, University of Washington, Seattle, WA, United States; ^6^ HIV Pathogenesis Research Unit, Department of Molecular Medicine and Haematology, University of the Witwatersrand, Johannesburg, Gauteng, South Africa; ^7^ Department of Pharmacology and Chemical Biology, School of Medicine, Emory University, Atlanta, GA, United States

**Keywords:** prodrugs, HIV, tenofovir, metabolism, lipids, nucleoside, nucleotide, antiviral

## Abstract

Nucleoside- and nucleotide-based therapeutics are indispensable treatment options for patients suffering from malignant and viral diseases. These agents are most commonly administered to patients as prodrugs to maximize bioavailability and efficacy. While the literature provides a practical prodrug playbook to facilitate the delivery of nucleoside and nucleotide therapeutics, small context-dependent amendments to these popular prodrug strategies can drive dramatic improvements in pharmacokinetic (PK) profiles. Herein we offer a brief overview of current prodrug strategies, as well as a case study involving the fine-tuning of lipid prodrugs of acyclic nucleoside phosphonate tenofovir (TFV), an approved nucleotide HIV reverse transcriptase inhibitor (NtRTI) and the cornerstone of combination antiretroviral therapy (cART). Installation of novel lipid terminal motifs significantly reduced fatty acid hepatic ω-oxidation while maintaining potent antiviral activity. This work contributes important insights to the expanding repertoire of lipid prodrug strategies in general, but particularly for the delivery and distribution of acyclic nucleoside phosphonates.

## 1 Introduction

**FIGURE 1:**
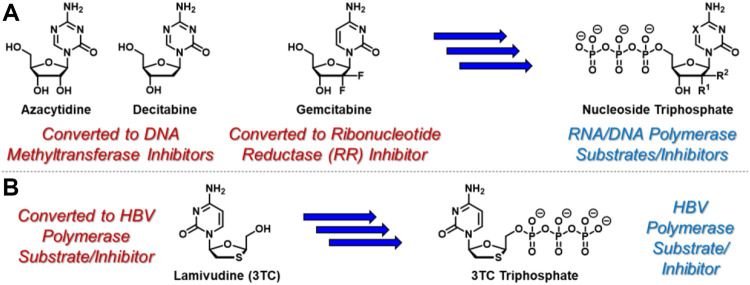
Selected anticancer nucleoside and nucleotide therapeutics and corresponding pleotropic mechanisms of action. **(A)** Azacytidine, decitabine, and gemcitabine are metabolically converted to the corresponding triphosphates which similarly incorporate into newly synthesized oligonucleotides. In addition, incorporated azacytidine and decitabine nucleotides inhibit DNA methyltransferases, while gemcitabine diphosphate inhibits ribonucleotide reductase. **(B)** Antiviral nucleoside-based therapeutic 3 TC is converted to its corresponding triphosphate, which is a suicide substrate for HBV polymerase.

Nucleoside- and nucleotide-based therapeutics represent a privileged class of anticancer (Shelton et al., 2016) and antiviral (Peterson and McKenna, 2009) agents and are critical components of modern clinical medicine that date back to the 1950’s (De Clercq, 2013). Synthetic nucleosides enter cells, either actively or passively, where they encounter nucleoside kinases that catalyze conversion to the corresponding nucleoside monophosphates (Stein and Moore, 2001). With few exceptions, subsequent kinase-mediated phosphorylation to the corresponding di- and triphosphates is generally required for therapeutic activation of nucleoside- and nucleotide-based therapies (Ray and Hostetler, 2011). For example, azacytidine and decitabine (Figure 1A), which are used clinically to treat myelodysplastic syndromes (Kaminskas et al., 2005) and acute myeloid leukemia (Welch et al., 2016), undergo conversion to the corresponding nucleoside triphosphates before incorporating into newly synthesized oligonucleotides and inhibiting DNA methyltransferase enzymes (Stresemann and Lyko, 2008). Mechanistically, this results in DNA hypomethylation and associated epigenetic alterations. In contrast, gemcitabine, a key therapeutic tool for clinical oncologists, elicits its cytotoxic activity at both nucleoside diphosphate and triphosphate stages (Mini et al., 2006). Similar to azacytidine and decitabine, gemcitabine is converted to the corresponding nucleoside triphosphate, leading to incorporation into growing strands of oligonucleotides and associated DNA/RNA damage. In addition, gemcitabine diphosphate is a potent inhibitor of ribonucleotide reductase (Wang et al., 2009), providing a complementary mechanism of cytotoxicity. Also similar to azacytidine and decitabine, hepatitis B virus (HBV)-targeting agent lamivudine (3TC, Figure 1B) requires conversion to the corresponding nucleoside triphosphate to exert its therapeutic effect as an HBV polymerase substrate and inhibitor (Dienstag et al., 1999). In contrast however, 3 TC is structurally unique because it features the opposite enantiomeric configuration of endogenous nucleosides. This unnatural stereochemical arrangement attractively facilitates efficient conversion to the corresponding nucleoside monophosphate, which is often reversible and the rate-limiting step during nucleoside analogue activation (Johnson et al., 1999). Although nucleoside- and nucleotide-based drugs are rarely classified as prodrugs, these example mechanisms of therapeutic activation indeed highlight these agents as a privileged class of prodrugs that are indispensable in modern healthcare (Jordheim et al., 2013).

**FIGURE 2:**
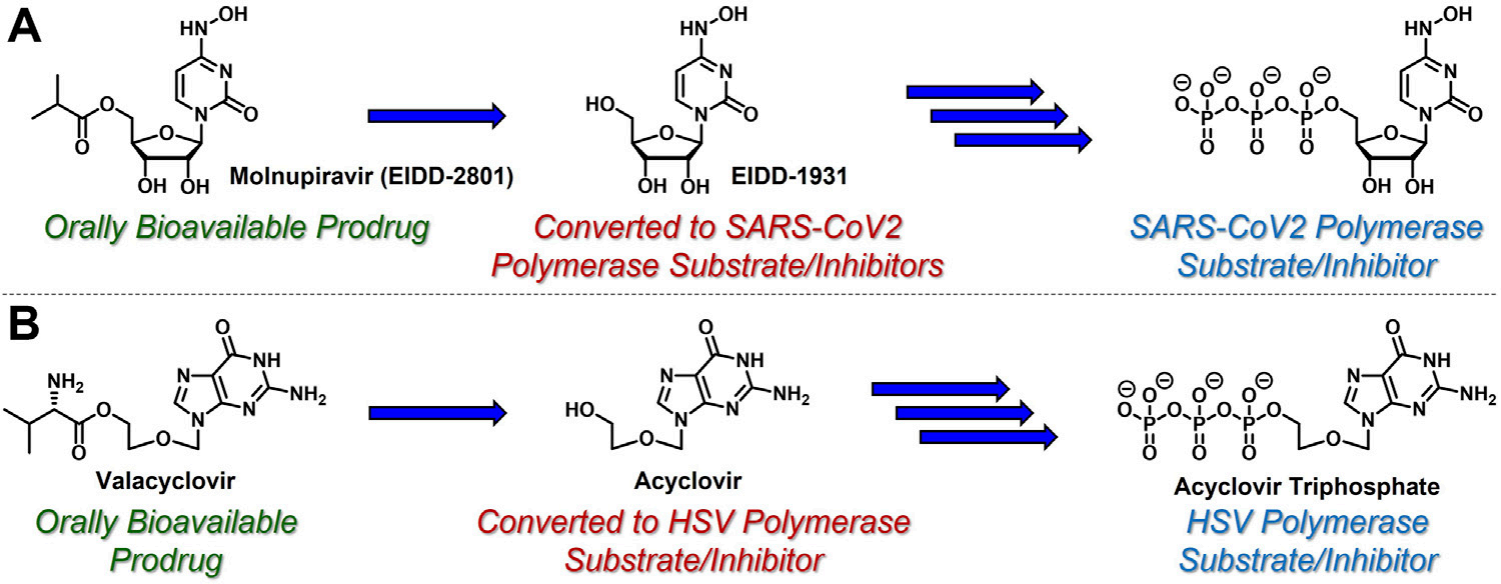
Nucleoside-based antiviral therapeutics **(A)** molnupiravir (EIDD-2801) and **(B)** valacyclovir are orally bioavailable prodrugs that release cyclic nucleoside EIDD-1931 and acyclic nucleoside acyclovir, respectively, both of which are then converted to the corresponding, viral polymerase-inhibiting triphosphates.

While azacytidine, decitabine, gemcitabine, and 3 TC are all prodrugs in and of themselves, nucleoside- and nucleotide-based therapeutics are often administered to patients using additional prodrug strategies to provide enhanced PK and oral delivery profiles (Thornton et al., 2016). For example, molnupiravir (EIDD-2801, Figure 2A) is an ester-based prodrug of *N*-hydroxycytidine (EIDD-1931) that demonstrated robust oral bioavailability and significant antiviral efficacy with a remarkably high barrier to resistance in human SARS-CoV-2 patients (Malone and Campbell, 2021). Similarly, valacyclovir (Valtrex) is an orally bioavailable amino acid-based prodrug of acyclic nucleoside acyclovir (Figure 2B) that is used to treat herpes simplex virus (Tyring et al., 2002) (HSV). Delivering EIDD-1931 and acyclovir using these ester- and amino acid-based prodrug tactics increases the concentration and duration of action of active nucleoside triphosphates in virally-infected cells. These are just two examples amongst a growing list of prodrug strategies reported for the delivery of nucleoside- and nucleotide-based therapeutics. This broad topic has been thoroughly reviewed elsewhere, for example, by Pradere et al. (2014). Wiemer (2020), Thornton et al (2016). and Peterson and McKenna, (2009). In contrast, this article highlights a few clinically effective strategies in the context of the case study described herein.

**FIGURE 3:**
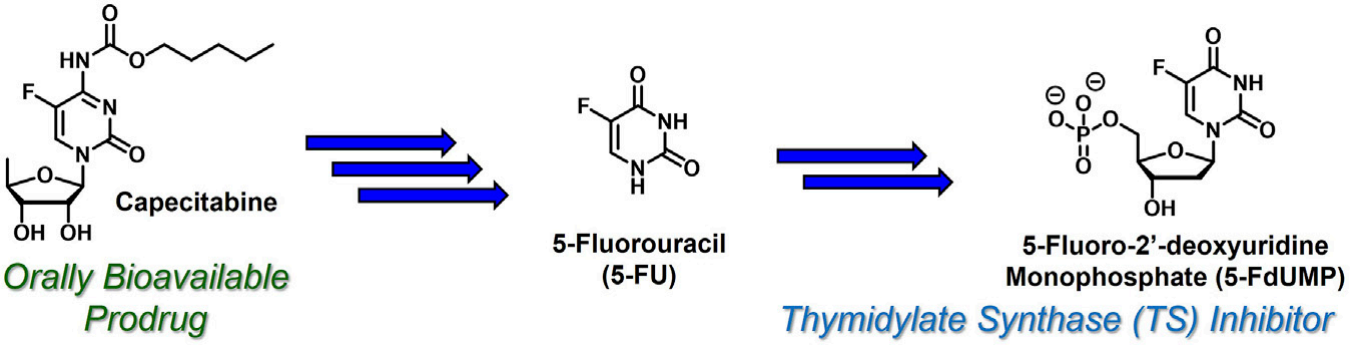
Capecitabine undergoes three enzymatic reactions to release 5-FU, which is then converted to TS inhibitor 5-FdUMP, which is uniquely active at the monophosphate stage.

Capecitabine (Walko and Lindley, 2005) (Figure 3) is an orally bioavailable prodrug of 5-fluorouracil (Longley et al., 2003) (5-FU) with dramatically improved safety and PK profiles relative to bolus 5-FU infusion. Administered to cancer patients beginning in 1962, 5-FU requires metabolic conversion to 5-fluoro-2'-deoxyuridine monophosphate (Santi et al., 1974) (5-FdUMP), which is uniquely active at the nucleoside monophosphate stage. 5-FdUMP covalently inhibits thymidylate synthase (TS)-mediated biosynthesis of thymidine, which reduces intracellular thymidine levels, inhibits DNA synthesis, and combats the proliferation of rapidly dividing cancer cells. Although 5-FU is effective, efficient hepatic metabolism by dihydropyrimidine dehydrogenase generates toxic metabolites and significantly limits oral bioavailability and duration of action (Miura et al., 2010). In contrast, substantially improved toxicity and oral PK profiles are observed with capecitabine (Mikhail et al., 2010), which requires three enzymatic reactions (mediated by carboxylesterase 1, cytidine deaminase, and thymidine phosphorylase) to release 5-FU. Capecitabine continues to be used clinically as an important component of various combination therapies to treat (most commonly) colorectal cancer.

**FIGURE 4:**
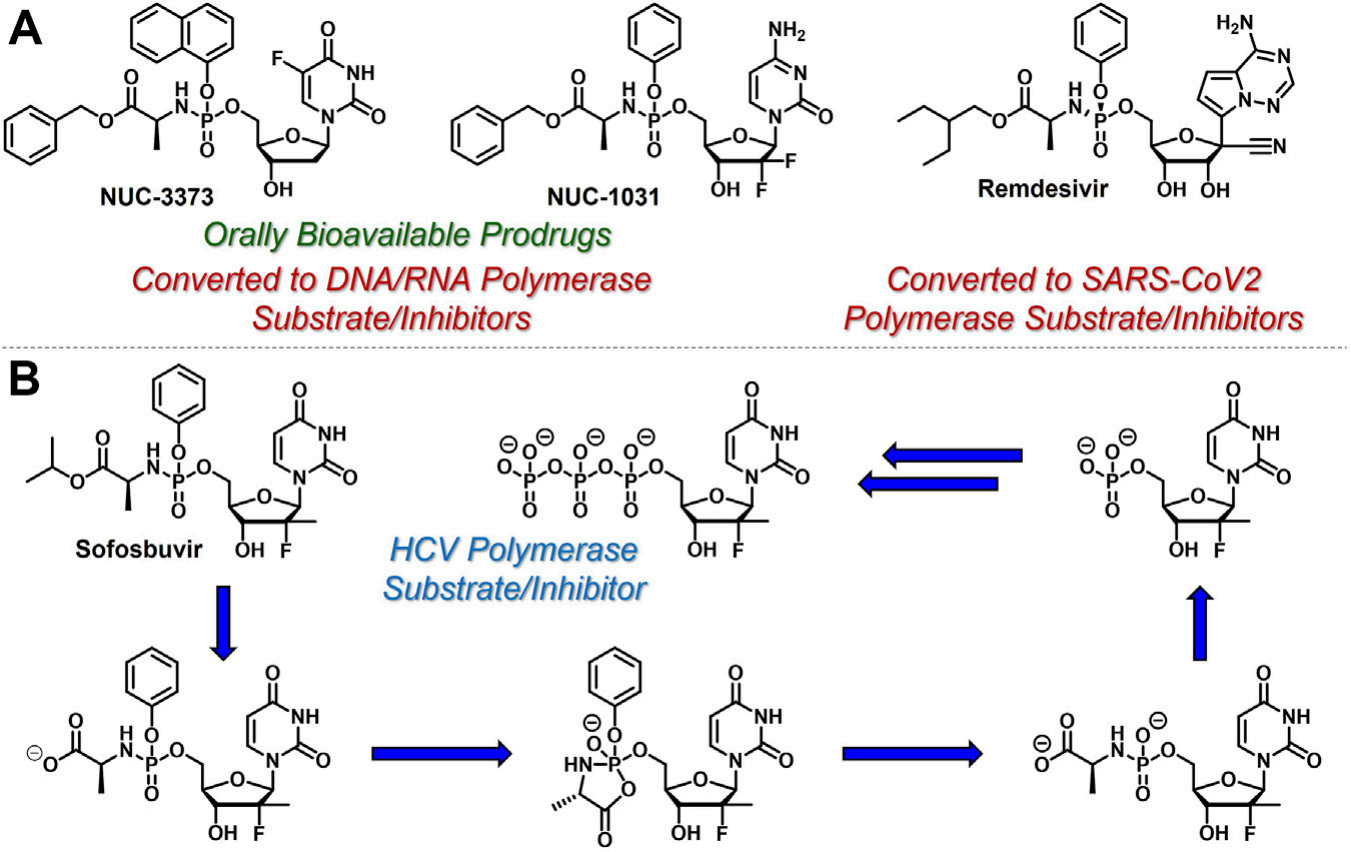
Selected structures of phosphoramidate or ProTide prodrugs of **(A)** anticancer (e.g., NUC- 3,373 and NUC-1031) and antiviral (e.g., remdesivir) nucleotide-based therapeutics. **(B)** The metabolic activation pathway of ProTides as highlighted by sofosbuvir.

Since conversion of nucleoside analogues to the corresponding monophosphates is typically both reversible and the rate-limiting step in the activation of nucleoside- and nucleotide-based therapeutics, slow phosphorylation kinetics could, in principle, be circumvented by directly administering nucleoside monophosphates (e.g., 5-FdUMP) to patients. However, these species are negatively charged at physiological pH and therefore are not orally bioavailable and do not efficiently achieve access to intracellular compartments (Pradere et al., 2014). To address these limitations, McGuigan and coworkers developed orally bioavailable phosphoramidate-based prodrugs of nucleoside monophosphates that mask the negative charges with so called ProTide technology (Mehellou et al., 2018). For example, Nucana’s ProTide of 5-FdUMP (Vande Voorde et al., 2011) (NUC-3373, Figure 4A) features a P-N bond that connects the nucleoside monophosphate phosphorus atom to the nitrogen atom of *l* -alanine benzyl ester. Similar substitution patterns are also exhibited by Nucana’s ProTide of gemcitabine (Kapacee et al., 2020) (NUC-1031), and Gilead’s remdesivir for SARS-CoV-2 infection (Wiemer, 2020). As further exemplified by Gilead’s sofosbuvir (McQuaid et al., 2015) (Figure 4B), which has been touted as a cure for hepatitis C virus (HCV), this class of ProTides relies on two sequential enzymatic reactions (i.e., esterase/peptidase-mediated hydrolysis followed by phosphoramidase-mediated hydrolysis) to activate and release the corresponding nucleoside monophosphates intracellularly. This prodrug strategy is particularly attractive because it bypasses the often sluggish first nucleoside phosphorylation step and masks the phosphate hydrophilicity to enable oral administration.

**FIGURE 5:**
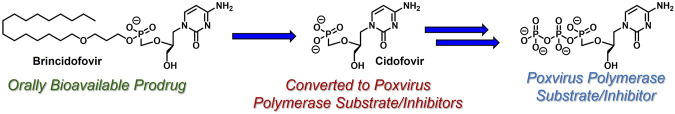
Acyclic nucleoside phosphonate cidofovir and its lipid-based prodrug brincidofovir, which can be administered orally to treat human poxvirus (e.g., smallpox and monkeypox infections).

Another common strategy to bypass the first kinase-mediated phosphorylation event involves the use of acyclic nucleoside phosphonates (De Clercq and Holý, 2005) (e.g., cidofovir, Figure 5). Like all acyclic nucleoside phosphonates, cidofovir mimics the corresponding nucleoside monophosphate with a metabolically stable C-P linkage, as opposed to the endogenous, metabolically labile O-P linkage. This atom swap disallows counterproductive cleavage back to nucleoside-like precursors, offering a unique advantage over the analogous nucleoside monophosphates. Interestingly and perhaps quite timely, brincidofovir, an orally bioavailable lipid-derived prodrug of cidofovir, achieved regulatory approval in June 2021 for the treatment of human poxvirus infections (Chan-Tack et al., 2021). Similar to ProTide technology, which delivers nucleoside monophosphates intracellularly, these lipid prodrugs partition into and cleave within pathogenic tissue by hijacking endogenous lipid trafficking and metabolic pathways (Porter et al., 2007). The therapeutic potential of this lipid prodrug tactic (reviewed elsewhere, for example, by Arouri et al. (2013). Hostetler, (2009), Trevaskis et al (2015). and Porter et al. (2007). will be highlighted in the case study presented below.

**FIGURE 6:**
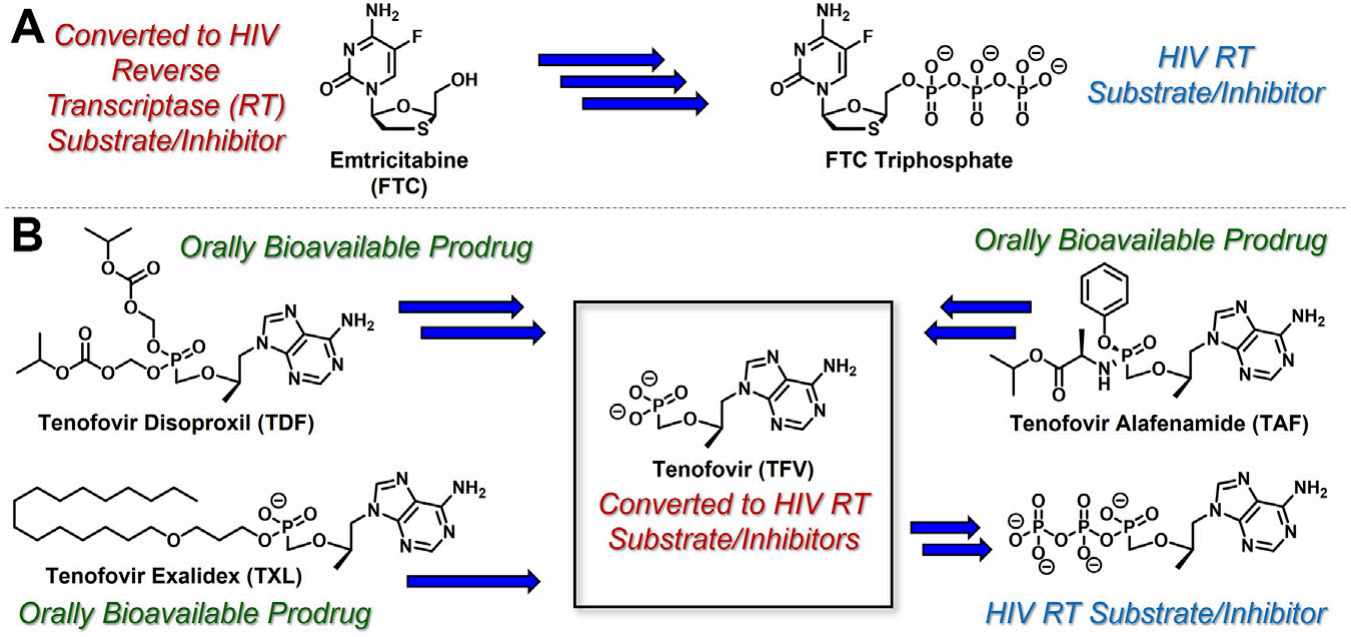
Antiretroviral **(A)** nucleoside and **(B)** nucleotide therapeutics developed to treat HIV infection.

While the convergence of two separate global public health emergencies since 2019 (i.e., outbreaks of SARS-CoV-2 and monkeypox infections) has necessarily taken hold of the drug development spotlight, we must not allow it to overshadow a currently uneradicated global pandemic that began in the 1980’s and continues today to affect 38 million patients across the globe: HIV/AIDS. In 1987, azidothymidine (AZT) became the first approved antiretroviral agent to treat HIV in the clinic (Yarchoan and Broder, 1987). AZT paved the way for other nucleoside and nucleotide HIV reverse transcriptase inhibitors (NRTIs and NtRTIs) with improved pharmacological profiles, including NRTI emtricitabine (Liotta and Painter, 2016) (FTC) and NtRTI TFV (Wainberg, 2013). Like 3TC, FTC (Figure 6A) adopts the unnatural nucleoside stereochemical configuration and inhibits HIV reverse transcriptase (RT) at the triphosphate stage. Similar to cidofovir on the other hand, TFV (Figure 6B) is an acyclic nucleoside phosphonate with a metabolically stable C-P bond and anionic phosphonate group. Because TFV is poorly orally bioavailable and cell membrane permeable, several of the prodrug strategies described above offer a variety of methods to alter PK profiles and improve oral bioavailability. Currently, TFV is administered orally as one of two FDA approved prodrugs, tenofovir disoproxil fumarate (Kearney et al., 2004) (TDF) or ProTide tenofovir alafenamide (Ray et al., 2016) (TAF). As TDF and TAF feature different prodrug motifs to mask the TFV phosphonate, distinct enzyme-mediated cleavage mechanisms are employed to release TFV into various physiological compartments after oral dose, some desired and some not. TDF is cleaved to TFV relatively non-specifically by esterases, which are particularly concentrated in the plasma (Augustinsson, 1961) and in the liver (Ecobichon and Kalow, 1962). Consequently, a significant fraction of each TDF dose is converted to TFV in plasma, as well as in hepatocytes, where some TFV remains trapped as TFV diphosphate (TFV-DP), while the rest escapes into the plasma. Plasma TFV then accumulates in the kidneys (Ray et al., 2006). Concentration of metabolites in these organs causes bone mineral density depletion (Gafni et al., 2006), renal toxicity (Fernandez-Fernandez et al., 2011), and changes in liver function (Ng et al., 2015) over the course of chronic treatment.

Improving drug safety by reducing TFV plasma levels, TAF (Figure 6B) is a ProTide and undergoes a sofosbuvir-like activation mechanism (Figure 4B). Although TFV plasma exposure is dramatically reduced relative to TDF (Wang et al., 2016), renal toxicity has already been observed clinically (Novick et al., 2017), despite FDA-approval of TAF only 6 years ago (Ray et al., 2016). This is perhaps not surprising, considering that in dogs, 17% of a single TAF dose was converted to plasma TFV (Lee et al., 2005), which is primed for bone and kidney sequestration. Furthermore, 65% was extracted by the liver (Babusis et al., 2013), concentrating in hepatocytes as TFV-DP (Murakami et al., 2015) due to cleavage by carboxylesterase 1^24^, encouraging hepatotoxicity (Alhankawi et al., 2018). As a consequence of this undesired metabolism, only 18% remained available to access HIV-infected cells, while 82% was sequestered for toxic side effects that compromise patient adherence to TFV prodrug-containing cART (Kim et al., 2015). This premature prodrug processing not only depletes significant fractions of each dose, but it also causes nephrotoxicity and bone mineral reduction due to organ specific accumulation of TFV and its phosphorylated metabolites. TDF- and TAF-containing cART also require strict, lifelong adherence due to frequent dosing regimens, presenting ample opportunity for the emergence of resistant virus (Ortego et al., 2011).

In parallel to the development of TAF, tenofovir Exalidex (Painter et al., 2007) (TXL), a lipid prodrug of TFV (Figure 6B) akin to brincidofovir (Figure 5) was identified to have similar benefits over TDF. However, unlike ProTide-based strategies, this lipid prodrug technology enables the potential for reduced frequency of dosing. As demonstrated in MRC-5 human lung fibroblasts by Aldern et al. (2003), brincidofovir delivered the active metabolite cidofovir diphosphate with an intracellular t_1/2_ of 10 days. Once weekly or biweekly antiretroviral dosing regimens could offer significant advantages over current cART by increasing patient compliance, elevating the barrier to resistance, and ultimately dramatically decreasing the number of HIV-infected patients that progress to AIDS (Nyaku et al., 2017). However, this potential for prolonged duration of action and reduced frequency of dosing is completely abrogated by rapid fatty acid metabolism in the liver. This is one of the major limitations of current lipid prodrug technology. Like endogenous lipids, brincidofovir and TXL undergo rapid hepatic ω-hydroxylation (Painter et al., 2008), which, like TAF, results in substantial extraction of each dose in the liver, accumulation of TFV in kidney, and concomitant organ-specific toxicities. To prevent this deleterious ω-oxidation mechanism of prodrug elimination, we previously installed metabolically inert motifs to disfavor the mechanism of metabolism (Pribut et al., 2021). Herein we further demonstrate that small modifications to alter the metabolic soft spots of lipid prodrugs can drive dramatic improvements in PK profiles.

## 2 Materials and methods

### 2.1 Organic synthesis

#### 2.1.1 General Chemical Synthesis and characterization

Anhydrous solvents were purchased from commercial sources and used without further drying or purification, unless otherwise noted. Room temperature (rt) was consistently measured to be = 25 ± 3°C. Automated column chromatography was performed using a Teledyne ISCO CombiFlash Companion system with RediSepRf normal-phase silica gel-packed columns or RediSepRf reverse-phase C18 gold columns (Teledyne Isco). Analytical thin-layer chromatography (TLC) was performed using commercially available (Sigma) aluminum-supported (thickness: 200 μm) or glass (2.5 × 7.5 cm) silica gel plates with fluorescent indicator (F-254). Visualization of compounds on TLC plates was accomplished using UV light (254 nm) and/or using ethanolic phosphomolybdic acid solution (PMA). NMR spectra (^1^H, ^13^C, ^19^F, and ^31^P) were obtained using either 600, 500, or 400 MHz Varian INOVA spectrometers, a 400 MHz Varian VNMR spectrometer, a 300 MHz Varian Mercury spectrometer, or a 600 MHz Bruker Avance Neo (Emory University NMR Center, directed by Dr. Shaoxiong Wu). NMR samples were prepared in deuterated chloroform (CDCl_3_) or deuterated methanol (CD_3_OD) using residual solvent peaks (CDCl_3_: ^1^H = 7.26 ppm, ^13^C = 77.2 ppm; CD_3_OD: ^1^H = 3.31 ppm, ^13^C = 49.0 ppm) for internal reference. Alternatively, the residual CHCl_3_ or CH_3_OH peak in ^1^H NMR was used as an absolute reference for ^31^P NMR and ^19^F NMR, unless otherwise specified. MestReNova software was used to process all NMR spectra. NMR data are reported to include chemical shifts (δ) reported in ppm, multiplicities indicated as s (singlet), d (doublet), t (triplet), q (quartet), m (multiplet), br (broad), or app (apparent), and coupling constants (*J*) reported in Hz. ^1^H NMR integrations are normalized to 1 proton. Overlapping carbon signals without distinct resonances are indicated below. High resolution mass spectrometry (HRMS) was performed by the Emory University Mass Spectrometry Center, directed by Dr. Fred Strobel. Liquid chromatography-mass spectrometry (LC-MS) was performed on an Agilent 1200 HPLC equipped with a 6,120 Quadrupole mass spectrometer (ESI) eluting with mixtures of HPLC grade CH_3_OH and H_2_O or CH_3_CN and H_2_O (all spiked with 0.1% HCO_2_H) through an analytical, reverse-phase, Agilent ZORBAX Eclipse XDB-C18 column (4.6 mm × 50 mm, 3.5 µm) or Agilent InfinityLab Poroshell 120 EC-C8 (2.1 mm × 50 mm, 2.7 µm) column. LC-MS samples were prepared in H_2_O/CH_3_OH mixtures. Final compound purity was assessed using NMR and LC-MS, and purity of all final compounds reported herein was determined to be ≥ 95% pure, except for compounds 32 and 42, which were determined to be 94% and 90% pure, respectively.

#### 2.1.2 Synthetic Procedures and compound characterization

##### 2.1.2.1 3-[(4-Methoxyphenyl)methoxy]propan-1-ol (1)

A solution of 1,3-propanediol (4.60 ml, 63.9 mmol, 2.0 equiv) in DMSO (50 ml) was added to a flask with a magnetic stir bar and subsequently cooled to 0°C. At this temperature, potassium hydroxide (KOH) pellets (3.58 g, 63.9 mmol, 2.0 equiv) were added in a portionwise fashion over approximately 5 min. The reaction mixture was then vigorously stirred at rt until most of the KOH pellets had dissolved before being treated with 4-methoxybenzyl chloride (4.30 ml, 31.9 mmol, 1.0 equiv) and then left to stir vigorously overnight. The next morning the mixture was cooled to 0°C, diluted with DCM and carefully quenched by the addition of 1 N aqueous HCl. The phases were separated, and the resulting aqueous layer was extracted with DCM. The organic phases were combined, dried over anhydrous MgSO4, filtered and then concentrated under reduced pressure. The crude material was purified by column chromatography eluting along a gradient of 10%–50% EtOAc in hexanes to afford a clear oil (4.26 g, 21.7 mmol, 68% yield). 1H NMR (500 MHz, CDCl3) δ 7.27–7.23 (m, 2H), 6.89–6.85 (m, 2H), 4.44 (s, 2H), 3.80 (s, 3H), 3.77–3.74 (m, 2H), 3.63 (t, J = 5.8 Hz, 2H), 2.44 (br s, 1H), 1.84 (p, J = 5.8 Hz, 2H). 13C NMR (126 MHz, CDCl3) δ 159.4, 130.3, 129.4, 114.0, 73.0, 69.2, 62.0, 55.4, 32.2 (9 out of 11 carbon signals observed due to overlapping signals lacking distinct resonances).

##### 2.1.2.2 1-[3-(5-Bromopentoxy)propoxymethyl]-4-methoxy-benzene (3)

3-[(4-Methoxyphenyl)methoxy]propan-1-ol (1, 2.30 ml, 10.2 mmol, 1.0 equiv) was added to a flask equipped with a magnetic stir bar and a reflux condenser and diluted with THF (20 ml) and saturated aqueous NaOH (20 ml). Tetrabutylammonium bromide (657 mg, 2.04 mmol, 0.20 equiv) and 1,5-dibromopentane (2.80 ml, 20.4 mmol, 2.0 equiv) were added, and the resulting reaction mixture was heated to 75°C, and stirred vigorously overnight. The following morning, the reaction mixture was cooled to rt and then partitioned between DCM and H2O. The resulting aqueous layer was extracted with DCM, and the combined organic phases were washed with brine, dried over anhydrous MgSO4, and concentrated under reduced pressure. The resulting crude material was purified by column chromatography eluting along a gradient of 0%–10% EtOAc in hexanes to yield a clear oil (1.74 g, 5.05 mmol, 50% yield). 1H NMR (500 MHz, CDCl3) δ 7.29–7.21 (m, 2H), 6.91–6.84 (m, 2H), 4.43 (s, 2H), 3.80 (s, 3H), 3.53 (t, J = 6.4 Hz, 2H), 3.50 (t, J = 6.4 Hz, 2H), 3.40 (app td, J = 6.6, 1.9 Hz, 4H), 1.93–1.79 (m, 4H), 1.62–1.53 (m, 2H), 1.53–1.44 (m, 2H). 13C NMR (126 MHz, CDCl3) δ 159.2, 130.7, 129.3, 113.8, 72.7, 70.6, 67.9, 67.1, 55.3, 33.8, 32.7, 30.2, 28.9, 25.0 (14 out of 16 carbon signals observed due to overlapping signals lacking distinct resonances). HRMS (APCI) m/z calculated for C16H24O379Br- [M–H]-, 343.09033 found, 343.09053.

##### 2.1.2.3 5-(5-Trityloxypentoxy)pentan-1-ol (4)

To a suspension of NaH (60% in mineral oil, 360 mg, 8.89 mmol, 1.5 equiv) in anhydrous DMF (8 ml) at 0°C was added neat 1,5-pentanediol (2.8 ml, 27 mmol, 4.5 equiv). After 5 min, the ice bath was removed and stirred at rt for 30 min. A solution of 5-(trityloxypentyl 4-methylbenzenesulfonate (Zhang et al., 2015; Lahav et al., 2017) (2, 5.3 ml, 5.9 mmol, 1.0 equiv) in anhydrous DMF (4 ml) was then added at rt and stirred overnight. The following morning, the reaction was cooled to 0°C, quenched with the slow addition of saturated ammonium chloride, and then extracted with EtOAc. The combined organic layers were rewashed with H_2_O, followed by brine. The organic layer was dried over Na_2_SO_4_, filtered, and concentrated under reduced pressure. The resulting crude material was purified by column chromatography eluting along a gradient of 0%–50% EtOAc in hexanes to afford a clear, viscous liquid (1.86 g, 4.32 mmol, 73% yield). ^1^H NMR (600 MHz, CDCl_3_) δ 7.46–7.44 (m, 6H), 7.31–7.27 (m, 6H), 7.24–7.21 (m, 3H), 3.64 (dt, *J* = 13.0, 6.5 Hz, 2H), 3.46–3.34 (m, 4H), 3.06 (td, *J* = 6.7, 5.3 Hz, 2H), 1.68–1.51 (m, 8H), 1.49–1.37 (m, 4H). ^13^C NMR (151 MHz, CDCl_3_) δ 144.4, 128.6, 127.6, 126.7, 86.2, 70.8, 70.7, 63.5, 62.7, 32.4, 29.8, 29.5, 29.4, 22.9, 22.4 (15 out of 29 carbon signals observed due to overlapping signals lacking distinct resonances). HRMS (ESI) m/z calculated for C_29_H_36_O_3_Na^+^ [M + Na]^+^, 455.25567 found, 455.25611.

##### 2.1.2.4 5-[5-[3-[(4-Methoxyphenyl)methoxy]propoxy]pentoxy]pentan-1-ol (5)

Pentane-1,5-diol (1.0 ml, 9.9 mmol, 2.0 equiv) was added to a flask equipped with a magnetic stir bar and a reflux condenser and diluted with THF (10 ml) and saturated aqueous NaOH (10 ml). Tetrabutylammonium bromide (318 mg, 0.985 mmol, 0.20 equiv) and 1-[3-(5-bromopentoxy)propoxymethyl]-4-methoxy-benzene (3, 1.70 g, 4.92 mmol, 1.0 equiv) were added, and the resulting reaction mixture was heated to 75°C, and stirred vigorously overnight. The following morning, the reaction mixture was cooled to rt and then partitioned between DCM and H2O. The resulting aqueous layer was extracted with DCM, and the combined organic phases were washed with brine, dried over anhydrous MgSO4 and concentrated under reduced pressure. The resulting crude material was purified by column chromatography eluting along a gradient of 0%–10% EtOAc in hexanes to yield a clear oil (798 mg, 2.17 mmol, 44% yield). 1H NMR (500 MHz, CDCl3) δ 7.24–7.18 (m, 2H), 6.86–6.80 (m, 2H), 4.39 (s, 2H), 3.75 (s, 3H), 3.59–3.53 (m, 2H), 3.49 (t, J = 6.3 Hz, 2H), 3.46 (t, J = 6.4 Hz, 2H), 3.41–3.32 (m, 6H), 2.49 (br s, 1H), 1.82 (p, J = 6.4 Hz, 2H), 1.58–1.49 (m, 8H), 1.43–1.28 (m, 4H). 13C NMR (126 MHz, CDCl3) δ 159.1, 130.6, 129.2, 113.7, 72.6, 70.9, 70.8, 70.8, 67.7, 67.1, 62.5, 55.2, 32.5, 30.1, 29.5, 29.5, 29.4, 22.8, 22.4 (19 out of 21 carbon signals observed due to overlapping signals lacking distinct resonances). HRMS (APCI) *m/z* calculated for C_21_H_37_O_5_^+^ [M + H]^+^, 369.26465 found, 369.26383.

##### 2.1.2.5 5-((5-(Octyloxy)pentyl)oxy)pentan-1-ol (6)

To a solution of 5-(5-trityloxypentoxy)pentan-1-ol (4, 890 mg, 2.06 mmol, 1.0 equiv) in toluene (16 ml) was added sequentially *n*-octylbromide (0.4 ml, 2.5 mmol, 1.2 equiv) and tetrabutylammonium hydrogen sulfate (349 mg, 1.03 mmol, 0.5 equiv) and 50% aqueous NaOH (4.0 ml) and heated at 65°C overnight. The following morning, the mixture was allowed to cool to rt, diluted with H_2_O, and extracted with EtOAc. The combined organic layers were rewashed with H_2_O and then brine. The organic layer was dried over Na_2_SO_4_, filtered, and concentrated under reduced pressure to obtain the crude mixture. Purification of the mixture by column chromatography eluting along a gradient of 0%–20% EtOAc in hexanes afforded an oil (670 mg, 1.22 mmol, 60% yield) which was used in the subsequent step. To (((5-((5-(octyloxy)pentyl)oxy)pentyl)oxy)methanetriyl)tribenzene (670 mg, 1.23 mmol, 1.0 equiv) was added 80% aqueous acetic acid (6 ml) and heated at 60°C for 2 h. White solid precipitated out during the reaction. The mixture was allowed to cool to rt, concentrated under reduced pressure, and co-concentrated with toluene to get the crude mixture. Purification of the mixture by column chromatography eluting along a gradient of 0%–50% EtOAc in hexanes afforded an oil (248 mg, 0.81 mmol, 67% yield). ^1^H NMR (600 MHz, CDCl_3_) δ 3.58 (t, *J* = 6.6 Hz, 2H), 3.40–3.31 (m, 8H), 2.26 (s, 1H), 1.58–1.49 (m, 10H), 1.42–1.31 (m, 4H), 1.31–1.16 (m, 10H), 0.83 (t, *J* = 7.0 Hz, 3H). ^13^C NMR (151 MHz, CDCl_3_) δ 71.1, 70.9, 70.8, 70.8, 62.6, 32.5, 31.9, 29.8, 29.6, 29.6, 29.5, 29.5, 29.3, 26.2, 22.9, 22.7, 22.5, 14.1. HRMS (ESI) *m*/*z* calculated for C_18_H_38_O_3_Na^+^ [M + Na]^+^, 325.27132 found, 325.27090.

##### 2.1.2.6 5-[5-[3-[(4-Methoxyphenyl)methoxy]propoxy]pentoxy]pentyl 4-methylbenzenesulfonate (7)

5-[5-[3-[(4-Methoxyphenyl)methoxy]propoxy]pentoxy]pentan-1-ol (5, 448 mg, 1.22 mmol, 1.0 equiv), Et3N (0.20 ml, 1.5 mmol, 1.2 equiv) and DMAP (1.5 mg, 0.010 mmol, 0.01 equiv) were added to an oven-dried flask equipped with a magnetic stir bar under an atmosphere of Ar and then diluted with DCM (5 ml). The resulting reaction mixture was cooled to before p-toluenesulfonyl chloride (278 mg, 1.46 mmol, 1.2 equiv) was added in a portionwise fashion over 5 min, and then left to stir vigorously at rt overnight. The following morning the reaction mixture was concentrated under reduced pressure and the resulting crude material was purified by column chromatography eluting along a gradient of 5%–40% EtOAc in hexanes to give a clear oil (444 mg, 0.850 mmol, 70% yield). 1H NMR (400 MHz, CDCl3) δ 7.72–7.65 (m, 2H), 7.27–7.18 (m, 2H), 7.18–7.12 (m, 2H), 6.81–6.73 (m, 2H), 4.33 (s, 2H), 3.92 (t, J = 6.5 Hz, 2H), 3.69 (s, 3H), 3.42 (app dt, J = 14.3, 6.4 Hz, 4H), 3.34–3.20 (m, 6H), 2.34 (s, 3H), 1.77 (p, J = 6.4 Hz, 2H), 1.62–1.52 (m, 2H), 1.52–1.35 (m, 6H), 1.34–1.21 (m, 4H). 13C NMR (126 MHz, CDCl3) δ 159.1, 144.6, 133.2, 130.7, 129.8, 129.1, 127.8, 113.7, 72.5, 70.8, 70.8, 70.5, 70.3, 67.7, 67.1, 55.2, 30.1, 29.5, 29.0, 28.6, 22.9, 22.1, 21.5 (23 out of 28 carbon signals observed due to overlapping signals lacking distinct resonances). HRMS (APCI) *m/z* calculated for C_28_H_43_O_7_^32^S^+^ [M + H]^+^, 523.27240 found, 523.27342.

##### 2.1.2.7 1-[3-[5-(5-Butoxypentoxy)pentoxy]propoxymethyl]-4-methoxy-benzene (8)

To an oven-dried flask equipped with a magnetic stir bar was added a solution of 1-butanol (0.30 ml, 3.1 mmol, 1.0 equiv) in DMF (10 ml) under an atmosphere of Ar and cooled to 0°C. NaH (60% in mineral oil, 149 mg, 3.72 mmol, 1.2 equiv) was added, and the resulting mixture was stirred at 0°C under Ar for 30 min. After this time, 5-[5-[3-[(4-methoxyphenyl)methoxy]propoxy]pentoxy]pentyl 4-methylbenzenesulfonate (7, 1.95 g, 3.72 mmol, 1.2 equiv) was added, and the reaction was allowed to slowly warm to rt and stirred vigorously overnight. The following morning, the reaction was quenched with saturated NH4Cl and then extracted with EtOAc. The organic phases were combined, washed with brine, dried over anhydrous MgSO4, filtered and then concentrated under reduced pressure. The resulting crude oil was then purified by column chromatography eluting along a gradient of 5%–20% EtOAc in hexanes to yield a clear oil (966 mg, 2.28 mmol, 73% yield). 1H NMR (500 MHz, CDCl3) δ 7.27–7.19 (m, 2H), 6.88–6.81 (m, 2H), 4.41 (s, 2H), 3.78 (s, 3H), 3.52 (t, J = 6.4 Hz, 2H), 3.48 (t, J = 6.4 Hz, 2H), 3.41–3.35 (m, 10H), 1.85 (p, J = 6.4 Hz, 2H), 1.62–1.49 (m, 10H), 1.43–1.29 (m, 6H), 0.90 (t, J = 7.4 Hz, 3H). 13C NMR (126 MHz, CDCl3) δ 159.2, 130.8, 129.3, 113.8, 72.7, 72.7, 72.7, 71.0, 70.9, 70.9, 70.8, 70.7, 67.8, 67.2, 55.3, 31.9, 30.3, 29.7, 29.6, 22.9, 22.9, 19.5, 14.0 (23 out of 25 carbon signals observed due to overlapping signals lacking distinct resonances). HRMS (APCI) *m/z* calculated for C_25_H_43_O_5_^−^ [M–H]^-^, 423.31050 found, 423.31071.

#### 2.1.3 General PMB deprotection procedure

In a flask equipped with a magnetic stir bar, PMB-protected alcohol (1.0 equiv) was dissolved in a (10:1) mixture of MeOH and H2O (0.2 M). The reaction was cooled to 0°C, and CAN (3.0 equiv) was added in a portionwise fashion over 5 min. The reaction mixture was then warmed to rt and stirred vigorously for 3 h, after which TLC confirmed the consumption of the starting material. The reaction was subsequently quenched with H2O and extracted with DCM. The combined organic phases were dried over anhydrous MgSO4 and concentrated under reduced pressure.

##### 2.1.3.1 3-[5-(5-Butoxypentoxy)pentoxy]propan-1-ol (9)

Synthesis was carried out according to the general PMB deprotection procedure using 1-[3-[5-(5-butoxypentoxy)pentoxy]propoxymethyl]-4-methoxy-benzene (8, 966 mg, 2.28 mmol) and was purified by column chromatography eluting along a gradient of 5%–40% EtOAc in hexanes to afford a pale yellow oil (425 mg, 1.37 mmol, 61% yield). 1H NMR (500 MHz, CDCl3) δ 3.73 (t, *J* = 5.6 Hz, 2H), 3.57 (t, *J* = 5.8 Hz, 2H), 3.43–3.33 (m, 10H), 2.65 (br s, 1H), 1.79 (p, *J* = 5.7 Hz, 2H), 1.60–1.48 (m, 10H), 1.41–1.29 (m, 6H), 0.88 (t, *J* = 7.4 Hz, 3H). 13C NMR (126 MHz, CDCl3) δ 71.3, 70.9, 70.9, 70.8, 70.7, 70.2, 62.1, 32.1, 31.9, 29.7, 29.7, 29.6, 29.6, 22.9, 22.9, 19.5, 14.0. HRMS (APCI) *m/z* calculated for C_17_H_37_O_4_^+^ [M + H]^+^, 305.26864 found, 305.26854.

#### 2.1.4 General TFV coupling procedure A

To a stirring suspension of TFV (1.0 equiv), DCC (2.0 equiv), and alcohol (1.2 equiv) in anhydrous NMP (0.2 M) under an Ar atmosphere was added DMAP (0.10 equiv). The resulting reaction mixture was stirred at rt for 10 min and then heated to 100°C overnight. The next morning, formation of the desired product was detected by LC-MS.

##### 2.1.4.1 Ammonium [(1R)-2-(6-aminopurin-9-yl)-1-methyl-ethoxy]methyl-[3-[5-(5-butoxypentoxy)pentoxy]propoxy]phosphinate (10)

Synthesis was carried out according to general TFV coupling procedure A using 3-[5-(5-butoxypentoxy)pentoxy]propan-1-ol (9, 127 mg, 0.420 mmol) and Et_3_N (0.10 ml, 0.70 mmol). Purification was carried out by column chromatography eluting along a gradient of 0%–100% 80:20:3 DCM:MeOH:NH4OH (solvent B) in DCM (solvent A). Fractions containing the desired product were collected, concentrated under reduced pressure, and then purified by reverse phase (C18) column chromatography eluting along a gradient of 10%–100% MeOH in H2O. Fractions containing the desired product were collected, concentrated under reduced pressure, stirred with 7 N ammonia in MeOH for 10 min at rt, and dried under vacuum to yield an off-white solid (51 mg, 0.086 mmol, 25% yield). 1H NMR (500 MHz, CD3OD) δ 8.30 (s, 1H), 8.20 (s, 1H), 4.37 (dd, *J* = 14.4, 3.2 Hz, 1H), 4.23 (dd, *J* = 14.4, 6.8 Hz, 1H), 3.93–3.81 (m, 3H), 3.71 (dd, *J* = 12.8, 9.5 Hz, 1H), 3.49–3.34 (m, 13H), 1.80–1.72 (m, 2H), 1.61–1.48 (m, 10H), 1.44–1.31 (m, 6H), 1.17 (d, *J* = 6.2 Hz, 3H), 0.92 (t, *J* = 7.4 Hz, 3H). 13C NMR (126 MHz, CD3OD) δ 157.2, 153.5, 151.0, 144.1, 119.6, 77.0 (d, *JCP* = 12.8 Hz), 71.9, 71.8, 71.6, 65.5 (d, *JCP* = 159.9 Hz), 63.1 (d, *JCP* = 5.7 Hz), 49.1, 32.9, 32.4 (d, *JCP* = 6.1 Hz), 30.6, 30.5, 23.9, 23.9, 20.4, 16.9, 14.2 (21 out of 26 carbon signals observed due to overlapping signals lacking distinct resonances). 31P NMR (162 MHz, CD3OD) δ 15.35. HRMS (ESI) *m/z* calculated for C26H49O7N5P+ [M + H]+, 574.33641 found, 574.33705. LC-MS (ESI, C8, 0.5 ml/min) 35%–95% MeCN in H2O (0.1% HCO2H), 6 min, RT = 0.880 min, *m/z* = 574.6 [M + H]+; 10%–95% MeCN in H2O (0.1% HCO2H), 6 min, RT = 2.671 min, *m/z* = 574.6 [M + H]+.

##### 2.1.4.2 Ammonium 5-(5-octoxypentoxy)pentoxy-[[(1R)-2-(6-aminopurin-9-yl)-1-methyl-ethoxy]methyl]phosphinate (11)

To a suspension of TFV (500 mg, 1.74 mmol, 1.0 equiv) in anhydrous DMF (3 ml) was added a drop of water followed by *N*,*N*-dimethylformamide dimethyl acetal (1.20 ml, 9.08 mmol, 5 equiv) and stirred at rt. After 3 h, DMF and excess reagent were removed by vacuum distillation. The resulting mixture was vacuum dried overnight to obtain a white gooey solid. In a flame dried microwave vial, a mixture of freshly prepared (*R*,*Z*)-*N*'-(9-(2-hydroxypropyl)-9*H*-purin-6-yl)-*N*,*N*-dimethylformimidamide (130 mg, 0.38 mmol, 1.2 equiv) and 5-(5-octoxypentoxy)pentan-1-ol (6, 100 mg, 0.330 mmol, 1.0 equiv) were dissolved in pyridine (4 ml) and added cyanotrichloromethane (2.0 ml, 20 mmol, 60 equiv). The resulting solution was heated in µwave at 80°C for 90 min. The reaction was left unstirred at rt overnight and was then quenched with H_2_O and stirred for 1 h. The reaction was then concentrated under reduced pressure, co-concentrated with toluene to get a brown crude mixture. The crude mixture was dissolved in methanol (5 ml), ammonium hydroxide (1.5 ml) was added, and stirred at rt overnight. The following morning, the mixture was concentrated under reduced pressure and purified by column chromatography eluting along a gradient of 0%-30% MeOH (spiked with 5% NH_4_OH) in DCM to afford a white solid (73 mg, 0.12 mmol, 37% yield). ^1^H NMR (399 MHz, CD_3_OD) δ 8.38 (s, 1H), 8.25 (s, 1H), 4.45 (dd, *J* = 14.4, 2.9 Hz, 1H), 4.26 (dd, *J* = 14.5, 6.9 Hz, 1H), 4.03 (td, *J* = 6.5, 2.8 Hz, 1H), 3.90–3.73 (m, 3H), 3.62 (dd, *J* = 12.9, 9.3 Hz, 1H), 3.40 (tdd, *J* = 6.4, 4.6, 1.4 Hz, 8H), 1.64–1.49 (m, 10H), 1.46–1.26 (m, 14H), 1.17 (d, *J* = 6.2 Hz, 3H), 0.96–0.83 (t, *J* = 6.8 Hz, 3H). ^13^C NMR (151 MHz, CD_3_OD) δ 153.2, 150.6, 147.3, 146.2, 119.4, 76.7 (d, *J*_*CP*_ = 11.9 Hz), 72.1 (d, *J*_*CP*_ = 13.2 Hz), 72.0, 66.2 (d, *J*_*CP*_ = 5.6 Hz), 65.0 (d, *J*_*CP*_ = 160.8 Hz), 33.2, 32.1 (d, *J*_*CP*_ = 5.6 Hz), 31.0, 30.8, 30.7, 30.6, 30.6, 27.5, 24.1, 23.9, 23.8, 17.2, 14.7 (23 out of 27 carbon signals observed due to overlapping signals lacking distinct resonances). ^31^P NMR (162 MHz, CD_3_OD) δ 16.10. HRMS (ESI) *m*/*z* calculated for C_27_H_51_O_6_N5P [M + H]^+^, 572.35715 found, 572.35616. LC-MS (ESI, C18, 1.0 ml/min) 85%–95% MeOH in H_2_O (0.1% HCO_2_H), 10 min, RT = 2.21 min, *m/z* = 572.4 [M + H]^+^; 75%–95% MeOH in H_2_O (0.1% HCO_2_H), 6 min, RT = 3.72 min, *m/z* = 572.2 [M + H]^+^.

#### 2.1.5 General TFV coupling procedure B

To an oven-dried flask equipped with a magnetic stir bar were added TFV (1.0 equiv), alcohol (1.0 equiv), EDC hydrochloride (2.0 equiv), and anhydrous DMF (0.3 M) at rt under an atmosphere of Ar. Et_3_N (2.0 equiv) and DMAP (0.1 equiv) were added, and the resulting reaction mixture was then heated to 90°C-105°C and stirred vigorously overnight.

##### 2.1.5.1 Ammonium 2,5,8,11,14,17-hexaoxanonadecan-19-yl (R)-(((1-(6-amino-9H-purin-9-yl)propan-2-yl)oxy)methyl)phosphonate (12)

Synthesis was carried out according to general TFV coupling procedure B using hexaethylene glycol monomethyl ether (206 mg, 0.696 mmol). Upon reaction completion, the reaction mixture was cooled to rt, quenched with H_2_O, and purified by column chromatography eluting along a gradient of 0%-85% 80:20:3 DCM:MeOH:NH_4_OH (solvent B) in DCM (solvent A). The product fractions were then collected and concentrated under reduced pressure. Finally, the resulting solid was washed with acetone and dried under vacuum to yield a white powder (131 mg, 0.231 mmol, 33% yield). ^1^H NMR (400 MHz, CD_3_OD) δ 8.32 (s, 1H), 8.21 (s, 1H), 4.38 (dd, *J* = 14.4, 3.1 Hz, 1H), 4.23 (dd, *J* = 14.4, 6.8 Hz, 1H), 3.96–3.83 (m, 3H), 3.72 (dd, *J* = 12.8, 9.4 Hz, 1H), 3.64–3.58 (m, 18H), 3.57–3.45 (m, 5H), 3.35 (s, 3H), 1.17 (d, *J* = 6.2 Hz, 3H). ^13^C NMR (151 MHz, CD_3_OD) δ 157.1, 153.3, 151.0, 144.3, 119.6, 76.9 (d, *J*_*CP*_ = 12.7 Hz), 72.8, 72.1 (d, *J*_*CP*_ = 6.1 Hz), 71.3, 71.3, 71.2, 71.1, 65.6 (d, *J*_*CP*_ = 159.8 Hz), 65.0 (d, *J*_*CP*_ = 5.5 Hz), 59.0, 16.9 (16 out of 22 carbon signals observed due to overlapping signals lacking distinct resonances). ^31^P NMR (162 MHz, CD_3_OD) δ 16.31. HRMS (NSI) *m/z* calculated for C_22_H_41_N_5_O_10_P^+^ [M + H]^+^: 566.25856, found 566.25871. LC-MS (ESI, C18, 0.5 ml/min) 25%–95% MeOH in H_2_O (0.1% HCO2H), 10 min, RT = 2.172 min, *m/z* = 566.2 [M + H]^+^; 5%–95% MeOH in H_2_O, 10 min, RT = 4.627 min, *m/z* = 566.2 [M + H]^+^.

##### 2.1.5.2 Ammonium [(1R)-2-(6-aminopurin-9-yl)-1-methyl-ethoxy]methyl-[2-[2-[2-(2-methoxyethoxy)ethoxy]ethoxy]ethoxy]phosphinate (13)

Synthesis was carried out according to general TFV coupling procedure B using 2-[2-[2-(2-methoxyethoxy)ethoxy]ethoxy]ethanol (0.15 ml, 0.69 mmol). Upon reaction completion, the reaction mixture was concentrated under reduced pressure. The resulting crude material was then taken up in a 1:1 mixture of DCM and 7 N ammonia in MeOH and stirred vigorously for 2 h before concentrating under reduced pressure. Purification was carried out by column chromatography eluting along a gradient of 0%–85% 80:20:3 DCM:MeOH:NH_4_OH (solvent B) in DCM (solvent A). Fractions containing the desired product were collected and concentrated under reduced pressure to afford a white solid (67 mg, 0.14 mmol, 20% yield). 1H NMR (400 MHz, CD3OD) δ 8.33 (s, 1H), 8.23 (s, 1H), 4.40 (dd, J = 14.4, 3.1 Hz, 1H), 4.24 (dd, *J* = 14.4, 7.0 Hz, 1H), 4.00–3.83 (m, 3H), 3.74 (dd, *J* = 12.9, 9.4 Hz, 1H), 3.64–3.59 (m, 10H), 3.58–3.48 (m, 5H), 3.35 (s, 3H), 1.17 (d, *J* = 6.2 Hz, 3H). 13C NMR (126 MHz, CD3OD) δ 156.8, 152.9, 150.9, 144.4, 119.4, 77.0 (d, *JCP* = 12.9 Hz), 72.8, 72.1 (d, *JCP* = 6.5 Hz), 71.3, 71.3, 71.1, 65.5 (d, *JCP* = 160.2 Hz), 65.1 (d, *JCP* = 5.8 Hz), 59.0, 49.1, 16.9 (16 out of 18 carbon signals observed due to overlapping signals lacking distinct resonances). 31P NMR (162 MHz, CD3OD) δ 15.81. HRMS (APCI) *m/z* calculated for C_18_H_31_N_5_O_8_P^−^ [M–H]^-^: 476.19157, found 476.19267. LC-MS: (ESI, C18, 0.5 ml/min) 10%–95% MeCN in H2O (0.1% HCO2H), 6 min, RT = 0.441 min, *m/z* = 478.4 [M + H]+; 5%–10% MeCN in H2O (0.1% HCO2H), 6 min, RT = 4.022 min, *m/z* = 478.4 [M + H]+.

##### 2.1.5.3 Ammonium [(1R)-2-(6-aminopurin-9-yl)-1-methyl-ethoxy]methyl-[2-(2-methoxyethoxy)ethoxy]phosphinate (14)

Synthesis was carried out according to general TFV coupling procedure B using diethylene glycol monomethyl ether (82 μL, 0.70 mmol). Upon reaction completion, the reaction mixture was concentrated under reduced pressure. The resulting crude material was then taken up in a 1:1 mixture of DCM and 7 N ammonia in MeOH and stirred vigorously for 2 h before concentrating under reduced pressure. Purification was carried out by column chromatography eluting along a gradient of 0%–85% 80:20:3 DCM:MeOH:NH_4_OH (solvent B) in DCM (solvent A). Fractions containing the desired product were collected and concentrated under reduced pressure to afford a white solid (70 mg, 0.17 mmol, 25% yield). 1H NMR (400 MHz, CD3OD) δ 8.33 (s, 1H), 8.23 (s, 1H), 4.40 (dd, *J* = 14.4, 3.2 Hz, 1H), 4.25 (dd, *J* = 14.4, 6.9 Hz, 1H), 3.99–3.86 (m, 3H), 3.74 (dd, *J* = 12.8, 9.4 Hz, 1H), 3.63–3.46 (m, 7H), 3.34 (s, 3H), 1.19 (d, *J* = 6.2 Hz, 3H). 13C NMR (126 MHz, CD3OD) δ 157.0, 153.2, 150.9, 144.2, 119.6, 77.1 (d, *JCP* = 13.0 Hz), 72.8, 72.0 (d, *JCP* = 6.7 Hz), 71.1, 65.6 (d, *JCP* = 160.7 Hz), 65.1 (d, *JCP* = 6.0 Hz), 59.0, 49.2, 16.9. 31P NMR (162 MHz, CD3OD) δ 15.68. HRMS (APCI) *m/z* calculated for C_14_H_23_N_5_O_6_P^−^ [M–H]^-^: 388.13914, found 388.14010. LC-MS: (ESI, C18, 0.5 ml/min) 5%–10% MeCN in H2O (0.1% HCO2H), 6 min, RT = 1.192 min, *m/z* = 390.3 [M + H]+; 5% isocratic MeCN in H2O (0.1% HCO2H), 5 min, RT = 1.192 min, *m/z* = 390.3 [M + H]+.

#### 2.1.6 General williamson etherification procedure

To an oven-dried flask equipped with a magnetic stir bar was added a solution of alcohol (1.5 equiv) in DMF (0.2 M) under an atmosphere of Ar. The reaction was cooled to 0°C, and NaH (60% in mineral oil, 1.5 equiv) was added in a portionwise fashion over 5 min. The resulting reaction mixture was stirred at 0°C under Ar for 30 min to 1 h. After this time, alkyl bromide (1.0 equiv) was added, and the reaction was allowed to slowly warm to rt and stirred vigorously overnight. The following morning, the reaction was quenched with H2O and then extracted with DCM. The organic phases were combined, washed with brine, dried over anhydrous MgSO4, filtered and then concentrated under reduced pressure.

##### 2.1.6.1 2-Heptadecoxyethanol (15)

Synthesis was carried out according to the general Williamson etherification procedure using ethylene glycol (0.26 ml, 4.7 mmol) and 1-bromoheptadecane (1.0 g, 3.1 mmol). Purification was carried out by column chromatography eluting along a gradient of 5%–30% EtOAc in hexanes to yield a white solid (237 mg, 0.789 mmol, 25% yield). 1H NMR (400 MHz, CDCl3) δ 3.74–3.69 (m, 2H), 3.55–3.50 (m, 2H), 3.46 (t, *J* = 6.7 Hz, 2H), 2.17 (br s, 1H), 1.62–1.53 (m, 2H), 1.37–1.21 (m, 28H), 0.89–0.84 (m, 3H). 13C NMR (126 MHz, CDCl3) δ 71.9, 71.6, 62.0, 32.1, 29.8, 29.8, 29.8, 29.7, 29.6, 29.5, 26.3, 22.8, 14.2 (13 out of 19 carbon signals observed due to overlapping signals lacking distinct resonances). HRMS (ESI) *m/z* calculated for C_19_H_40_O_2_^23^Na^+^ [M + Na]^+^: 323.29205, found 323.29129.

##### 2.1.6.2 4-Pentadecoxybutan-1-ol (16)

Synthesis was carried out according to the general Williamson etherification procedure using butane-1,4-diol (0.24 ml, 2.6 mmol) and 1-bromopentadecane (0.50 ml, 1.7 mmol). Purification was carried out by column chromatography eluting along a gradient of 5%–30% EtOAc in hexanes to yield a white solid (405 mg, 1.35 mmol, 79% yield). 1H NMR (500 MHz, CDCl3) δ 3.66–3.59 (m, 2H), 3.47–3.38 (m, 4H), 2.56 (br s, 1H), 1.71–1.62 (m, 4H), 1.60–1.53 (m, 2H), 1.34–1.21 (m, 24H), 0.90–0.84 (m, 3H). 13C NMR (126 MHz, CDCl3) δ 71.4, 71.0, 62.9, 32.1, 30.6, 29.8, 29.8, 29.8, 29.8, 29.8, 29.7, 29.6, 29.5, 27.2, 26.3, 22.8, 14.2 (17 out of 19 carbon signals observed due to overlapping signals lacking distinct resonances). HRMS (APCI) *m/z* calculated for C_19_H_41_O_2_^+^ [M + H]^+^: 301.31011, found 301.30989.

##### 2.1.6.3 5-Tetradecoxypentan-1-ol (17)

Synthesis was carried out according to the general Williamson etherification procedure using pentane-1,5-diol (0.28 ml, 2.7 mmol) and 1-bromotetradecane (500 mg, 1.80 mmol). Purification was carried out by column chromatography eluting along a gradient of 5%–30% EtOAc in hexanes to yield a white solid (257 mg, 0.856 mmol, 47% yield). 1H NMR (400 MHz, CDCl3) δ 3.60 (t, *J* = 6.5 Hz, 2H), 3.37 (q, *J* = 6.8 Hz, 4H), 2.14 (br s, 1H), 1.63–1.49 (m, 6H), 1.45–1.34 (m, 2H), 1.32–1.18 (m, 22H), 0.88–0.81 (m, 3H). 13C NMR (126 MHz, CDCl3) δ 71.2, 70.9, 62.7, 32.6, 32.0, 29.8, 29.8, 29.8, 29.8, 29.8, 29.7, 29.6, 29.5, 29.5, 26.3, 22.8, 22.5, 14.2 (18 out of 19 carbon signals observed due to overlapping signals lacking distinct resonances). HRMS (ESI) *m/z* calculated for C_19_H_40_O_2_^23^Na^+^ [M + Na]^+^: 323.29205, found 323.29105.

#### 2.1.7 General TFV coupling procedure C

TFV (1.0 equiv) and pyridine (0.2 M) were added to an oven-dried flask equipped with a magnetic stir bar under an atmosphere of Ar. The alcohol (1.5 equiv) was subsequently added, followed by trisyl chloride (3.0 equiv), and the resulting reaction mixture was stirred vigorously at rt for 48 h under Ar. After this time, the reaction mixture was concentrated under reduced pressure, taken up in saturated aqueous NH_4_Cl, stirred vigorously at rt for 15 min, and then concentrated under reduced pressure. The resulting salt was then vigorously stirred in a 4:1 mixture of DCM and MeOH for 1 h. The resulting solution was filtered, and the resulting mother liquor was concentrated under reduced pressure.

##### 2.1.7.1 Ammonium 2-heptadecoxyethoxy-[[(1R)-2-(6-aminopurin-9-yl)-1-methyl-ethoxy]methyl]phosphinate (18)

Synthesis was carried out according to general TFV coupling procedure C using 2-heptadecoxyethanol (15, 188 mg, 0.627 mmol) and purified by column chromatography eluting along a gradient of 0%–100% 80:20:3 DCM:MeOH:NH4OH (solvent B) in DCM (solvent A). Fractions containing the desired product were collected, concentrated under reduced pressure, and then purified by reverse phase (C18) column chromatography eluting along a gradient of 10%–100% MeOH in H2O. Fractions containing the desired product were collected, concentrated under reduced pressure, stirred with 7 N ammonia in MeOH for 10 min at rt, and dried under vacuum to yield a white solid (58 mg, 0.099 mmol, 24% yield). 1H NMR (600 MHz, CD3OD) δ 8.33 (s, 1H), 8.20 (s, 1H), 4.38 (dd, *J* = 14.4, 3.2 Hz, 1H), 4.23 (dd, *J* = 14.4, 6.6 Hz, 1H), 3.96–3.86 (m, 3H), 3.73 (dd, *J* = 12.8, 9.4 Hz, 1H), 3.53–3.44 (m, 3H), 3.41–3.34 (m, 2H), 1.51–1.44 (m, 2H), 1.34–1.23 (m, 28H), 1.15 (d, *J* = 6.3 Hz, 3H), 0.90 (t, *J* = 7.0 Hz, 3H). 13C NMR (151 MHz, CD3OD) δ 157.2, 153.5, 151.0, 144.3, 119.6, 76.9 (d, *JCP* = 12.7 Hz), 72.3, 71.7 (d, *JCP* = 6.8 Hz), 65.7 (d, *JCP* = 160.2 Hz), 65.1 (d, *JCP* = 5.6 Hz), 33.1, 30.8, 30.8, 30.8, 30.8, 30.7, 30.6, 30.5, 27.2, 23.7, 16.8, 14.4 (22 out of 28 carbon signals observed due to overlapping signals lacking distinct resonances). 31P NMR (162 MHz, CD3OD) δ 15.52. HRMS (APCI) *m/z* calculated for C28H51O5N5P- [M–H]-, 568.36333 found, 568.36321. LC-MS: (ESI, C8, 0.5 ml/min) 60%–95% MeCN in H2O (0.1% HCO2H), 6 min, RT = 1.589 min, *m/z* = 570.6 [M + H]+; 40%–95% MeCN in H2O (0.1% HCO2H), 6 min, RT = 4.405 min, *m/z* = 570.6 [M + H]+.

##### 2.1.7.2 Ammonium 4-pentadecoxybutoxy-[[(1R)-2-(6-aminopurin-9-yl)-1-methyl-ethoxy]methyl]phosphinate (19)

Synthesis was carried out according to general TFV coupling procedure C using 4-pentadecoxybutan-1-ol (16, 392 mg, 1.31 mmol) and purified by column chromatography eluting along a gradient of 0%–100% 80:20:3 DCM:MeOH:NH4OH (solvent B) in DCM (solvent A). Fractions containing the desired product were collected, concentrated under reduced pressure, and then purified by reverse phase (C18) column chromatography eluting along a gradient of 10%–100% MeOH in H2O. Fractions containing the desired product were collected, concentrated under reduced pressure, stirred with 7 N ammonia in MeOH for 10 min at rt, and dried under vacuum to yield a white solid (278 mg, 0.474 mmol, 54% yield). 1H NMR (600 MHz, CD3OD) δ 8.31 (s, 1H), 8.20 (s, 1H), 4.37 (dd, *J* = 14.4, 3.2 Hz, 1H), 4.23 (dd, *J* = 14.4, 6.7 Hz, 1H), 3.93–3.86 (m, 1H), 3.83–3.74 (m, 2H), 3.71 (dd, *J* = 12.7, 9.5 Hz, 1H), 3.47 (dd, *J* = 12.7, 10.0 Hz, 1H), 3.39–3.35 (m, 4H), 1.60–1.54 (m, 4H), 1.54–1.48 (m, 2H), 1.28 (s, 24H), 1.16 (d, *J* = 6.2 Hz, 3H), 0.90 (t, *J* = 7.0 Hz, 3H). 13C NMR (151 MHz, CD3OD) δ 157.2, 153.5, 151.0, 144.3, 119.6, 76.9 (d, *JCP* = 13.0 Hz), 71.9, 71.5, 65.7 (d, *JCP* = 5.7 Hz), 65.5 (d, *JCP* = 159.9 Hz), 33.1, 30.8, 30.8, 30.8, 30.8, 30.8, 30.7, 30.7, 30.6, 30.5, 28.9, 28.8, 27.3, 27.1, 23.7, 16.8, 14.4 (27 out of 28 carbon signals observed due to overlapping signals lacking distinct resonances). 31P NMR (162 MHz, CD3OD) δ 15.25. HRMS (APCI) m/z calculated for C28H51O5N5P- [M–H]-, 568.36333 found, 568.36270. LC-MS: (ESI, C8, 0.5 ml/min) 60%–95% MeCN in H2O (0.1% HCO2H), 6 min, RT = 1.313 min, m/z = 570.6 [M + H]+; 40%–95% MeCN in H2O (0.1% HCO2H), 6 min, RT = 4.102 min, *m/z* = 570.6 [M + H]+.

##### 2.1.7.3 Ammonium [(1R)-2-(6-aminopurin-9-yl)-1-methyl-ethoxy]methyl-(5-tetradecoxypentoxy)phosphinate (20)

Synthesis was carried out according to general TFV coupling procedure C using 5-tetradecoxypentan-1-ol (17, 188 mg, 0.627 mmol) and purified by column chromatography eluting along a gradient of 0%–100% 80:20:3 DCM:MeOH:NH4OH (solvent B) in DCM (solvent A). Fractions containing the desired product were collected, concentrated under reduced pressure, and then purified by reverse phase (C18) column chromatography eluting along a gradient of 10%–100% MeOH in H2O. Fractions containing the desired product were collected, concentrated under reduced pressure, stirred with 7 N ammonia in MeOH for 10 min at rt, and dried under vacuum to yield a white solid (85 mg, 0.15 mmol, 35% yield). 1H NMR (600 MHz, CD3OD) δ 8.31 (s, 1H), 8.20 (s, 1H), 4.37 (dd, *J* = 14.4, 3.2 Hz, 1H), 4.23 (dd, *J* = 14.4, 6.8 Hz, 1H), 3.93–3.86 (m, 1H), 3.79–3.68 (m, 3H), 3.46 (dd, *J* = 12.7, 10.1 Hz, 1H), 3.37 (q, *J* = 6.4 Hz, 4H), 1.56–1.49 (m, 6H), 1.36–1.26 (m, 24H), 1.16 (d, *J* = 6.2 Hz, 3H), 0.90 (t, *J* = 7.0 Hz, 3H). 13C NMR (151 MHz, CD3OD) δ 157.2, 153.5, 151.0, 144.2, 119.6, 76.9 (d, *JCP* = 12.8 Hz), 72.0, 71.8, 65.8 (d, *JCP* = 5.9 Hz), 65.5 (d, *JCP* = 159.7 Hz), 33.1, 31.9, 31.9, 30.8, 30.8, 30.8, 30.7, 30.6, 30.5, 30.5, 27.3, 23.7, 23.6, 16.8, 14.4 (25 out of 28 carbon signals observed due to overlapping signals lacking distinct resonances). 31P NMR (162 MHz, CD3OD) δ 15.27. HRMS (APCI) *m/z* calculated for C28H51O5N5P- [M–H]-, 568.36333 found, 568.36284. LC-MS: (ESI, C8, 0.5 ml/min) 60%–95% MeCN in H2O (0.1% HCO2H), 6 min, RT = 1.171 min, *m/z* = 570.6 [M + H]+; 40%–95% MeCN in H2O (0.1% HCO2H), 6 min, RT = 3.960 min, *m/z* = 570.6 [M + H]+.

##### 2.1.7.4 Ammonium 2-octadecoxyethoxy-[[(1R)-2-(6-aminopurin-9-yl)-1-methyl-ethoxy]methyl]phosphinate (21)

Synthesis was carried out according to general TFV coupling procedure A using 2-octadecoxyethanol (131 mg, 0.420 mmol) and purified by column chromatography eluting along a gradient of 0%–100% 80:20:3 DCM:MeOH:NH4OH (solvent B) in DCM (solvent A). Fractions containing the desired product were collected, concentrated under reduced pressure, and then purified by reverse phase (C18) column chromatography eluting along a gradient of 10%–100% MeOH in H2O. Fractions containing the desired product were collected, concentrated under reduced pressure, stirred with 7 N ammonia in MeOH for 10 min at rt, and dried under vacuum to yield an off-white solid (104 mg, 0.173 mmol, 50% yield). 1H NMR (400 MHz, CD3OD) δ 8.34 (s, 1H), 8.20 (s, 1H), 4.39 (dd, *J* = 14.4, 3.2 Hz, 1H), 4.23 (dd, *J* = 14.4, 6.6 Hz, 1H), 3.97–3.86 (m, 3H), 3.73 (dd, *J* = 12.8, 9.4 Hz, 1H), 3.54–3.44 (m, 3H), 3.42–3.33 (m, 2H), 1.48 (p, *J* = 6.6 Hz, 2H), 1.35–1.21 (m, 30H), 1.15 (d, *J* = 6.3 Hz, 3H), 0.90 (t, *J* = 7.0 Hz, 3H). 13C NMR (151 MHz, CD3OD) δ 157.0, 153.2, 150.9, 144.4, 119.5, 76.9 (d, *JCP* = 12.7 Hz), 72.3, 71.7 (d, *JCP* = 6.7 Hz), 65.7 (d, *JCP* = 160.5 Hz), 65.1 (d, *JCP* = 5.6 Hz), 33.1, 30.8, 30.8, 30.8, 30.8, 30.7, 30.6, 30.5, 27.2, 23.7, 16.8, 14.4 (22 out of 29 carbon signals observed due to overlapping signals lacking distinct resonances). 31P NMR (243 MHz, CD3OD) δ 15.51. HRMS (APCI) *m/z* calculated for C_₂₉_H_₅₃_O_₅_N_₅_P- [M–H]-, 582.37898 found, 582.37888. LC-MS: (ESI, C8, 0.5 ml/min) 60%–95% MeCN in H2O (0.1% HCO2H), 6 min, RT = 2.159 min, *m/z* = 584.7 [M + H]+; 40%–95% MeCN in H2O (0.1% HCO2H), 6 min, RT = 4.833 min, m/z = 584.7 [M + H]+.

#### 2.1.8 General Procedure for the formation of terminal alkyne intermediates (25–27)

In an oven-dried flask equipped with a magnetic stir bar, pyridine (2.0 equiv) and then *p*-toluenesulfonyl chloride (1.5 equiv) were added to a solution of alcohol (1.0 equiv) in DCM (0.2 M) at 0°C under an atmosphere of Ar. The reaction mixture was then slowly warmed to rt and stirred vigorously overnight. The following morning, the reaction was diluted with DCM and quenched with H_2_O. The phases were then separated, and the organic phase was sequentially washed with 2 M aqueous HCl, saturated aqueous NaHCO_3_, H_2_O and brine. The organic phase was then dried over Na_2_SO_4_, filtered, concentrated under reduced pressure and purified by column chromatography eluting along a gradient of 0%–10% EtOAc in hexanes to afford the tosylated alcohol as a white solid. To a solution of 2-[(4-methoxyphenyl)methoxy]ethanol (1.2 equiv) in DMF (0.3 M) at 0°C under an atmosphere of Ar was added NaH (60% in mineral oil, 1.2 equiv). After vigorous stirring at 0°C for 30 min, tosylated alcohol (1.0 equiv) was added and the resulting suspension was slowly warmed to rt and stirred vigorously overnight. The following morning, the reaction was quenched with saturated aqueous NH_4_Cl and extracted with DCM. The organic phases were combined, dried over Na_2_SO_4_, filtered and concentrated under reduced pressure. The resulting crude material was then purified by column chromatography eluting along a gradient of 20%–80% DCM in hexanes.

##### 2.1.8.1 1-(2-Pentadec-14-ynoxyethoxymethyl)-4-methoxy-benzene (25)

Synthesis was carried out according to the general procedure for the formation of terminal alkyne intermediates starting from pentadec-14-yn-1-ol (22, 1.70 g, 7.58 mmol) and purified by column chromatography eluting along a gradient of 20%–80% DCM in hexanes to afford a white solid (1.82 g, 4.67 mmol, 62% yield over 2 steps). ^1^H NMR (600 MHz, CDCl_3_) δ 7.29 (d, *J* = 8.7 Hz, 2H), 6.89 (d, *J* = 8.6 Hz, 2H), 4.52 (s, 2H), 3.82 (s, 3H), 3.61 (app s, 4H), 3.47 (t, *J* = 6.8 Hz, 2H), 2.19 (td, *J* = 7.2, 2.7 Hz, 2H), 1.95 (t, *J* = 2.6 Hz, 1H), 1.64–1.57 (m, 2H), 1.54 (p, *J* = 7.2 Hz, 2H), 1.44–1.37 (m, 2H), 1.37–1.24 (m, 16H). ^13^C NMR (151 MHz, CDCl_3_) δ 159.2, 130.5, 129.4, 113.8, 84.8, 72.9, 71.6, 70.2, 69.1, 68.1, 55.3, 29.7, 29.6, 29.6, 29.6, 29.5, 29.1, 28.8, 28.5, 26.1, 18.4 (21 out of 25 carbon signals observed due to overlapping signals lacking distinct resonances). HRMS (APCI) *m/z* calculated for C_25_H_40_O₃^+·^ [M]^+·^, 388.29775 found, 388.29665.

##### 2.1.8.2 1-(2-Hexadec-15-ynoxyethoxymethyl)-4-methoxy-benzene (26)

Synthesis was carried out according to the general procedure for the formation of terminal alkyne intermediates starting from hexadec-15-yn-1-ol (23, 1.60 g, 6.71 mmol) and purified by column chromatography eluting along a gradient of 20%–80% DCM in hexanes to afford a white solid (2.20 g, 5.46 mmol, 81% yield over 2 steps). ^1^H NMR (600 MHz, CDCl_3_) δ 7.29 (d, *J* = 8.3 Hz, 2H), 6.89 (d, *J* = 8.4 Hz, 2H), 4.52 (s, 2H), 3.82 (br s, 3H), 3.61 (app d, *J* = 1.1 Hz, 4H), 3.47 (t, *J* = 6.8 Hz, 2H), 2.20 (td, *J* = 7.2, 2.6 Hz, 2H), 1.97–1.93 (m, 1H), 1.60 (p, *J* = 6.9 Hz, 2H), 1.54 (p, *J* = 7.2 Hz, 2H), 1.40 (p, *J* = 6.9 Hz, 2H), 1.37–1.24 (m, 18H). ^13^C NMR (151 MHz, CDCl_3_) δ 159.2, 130.5, 129.4, 113.8, 84.8, 72.9, 71.6, 70.2, 69.1, 68.1, 55.3, 29.7, 29.7, 29.7, 29.6, 29.5, 29.1, 28.8, 28.5, 26.1, 18.4 (21 out of 26 carbon signals observed due to overlapping signals lacking distinct resonances). HRMS (APCI) *m/z* calculated for C_26_H_42_O₃^+·^ [M]^+·^, 403.32067 found, 403.32114.

##### 2.1.8.3 1-(2-Dodec-11-ynoxyethoxymethyl)-4-methoxy-benzene (27)

Synthesis was carried out according to the general procedure for the formation of terminal alkyne intermediates starting from dodec-11-yn-1-ol (24, 500 mg, 2.74 mmol) and purified by column chromatography eluting along a gradient of 20%–80% DCM in hexanes to afford a white solid (370 mg, 1.07 mmol, 39% yield over 2 steps). ^1^H NMR (400 MHz, CDCl_3_) δ 7.30–7.21 (m, 2H), 6.91–6.81 (m, 2H), 4.49 (br s, 2H), 3.78 (br s, 3H), 3.57 (app d, *J* = 1.1 Hz, 4H), 3.43 (m, 2H), 2.16 (m, 2H), 1.92 (m, 1H), 1.65–1.45 (m, 4H), 1.41–1.21 (m, 12H). HRMS (APCI) *m/z* calculated for C_22_H_35_O_3_^+^ [M + H]^+^, 347.25807 found, 347.25831.

##### 2.1.8.4 1-Methoxy-4-[2-(16,16,16-trifluorohexadec-14-ynoxy)ethoxymethyl]benzene (28)

An oven-dried flask equipped with a magnetic stir bar were charged with CuI (1.32 g, 6.95 mmol, 1.5 equiv), K_2_CO_3_ (1.92 g, 13.9 mmol, 3.0 equiv), TMEDA (1.1 ml, 7.1 mmol, 1.5 equiv), and DMF (10 ml) under a balloon of air. The resulting blue mixture was stirred vigorously at rt for 15 min. Trifluoromethyltrimethylsilane (1.40 ml, 9.26 mmol, 2.0 equiv) was added, and the resulting reaction mixture was stirred for an additional 5 min at rt before cooling to 0°C. A solution of 1-(2-pentadec-14-ynoxyethoxymethyl)-4-methoxy-benzene (25, 1.80 g, 4.63 mmol, 1.0 equiv) and trifluoromethyltrimethylsilane (1.40 ml, 9.26 mmol, 2.0 equiv) in DMF (10 ml) was added, and the resulting reaction mixture was allowed to warm to rt and stirred vigorously for 48 h. After this time, the reaction was quenched with H_2_O and extracted with DCM. The organic phases were combined, dried over anhydrous Na_2_SO_4_, filtered, and concentrated under reduced pressure. The crude material was subsequently purified by column chromatography eluting along a gradient of 5–20% EtOAc in hexanes to yield a white solid (1.80 g, 3.94 mmol, 85% yield). ^1^H NMR (600 MHz, CDCl_3_) δ 7.29 (d, *J* = 8.6 Hz, 2H), 6.89 (d, *J* = 8.6 Hz, 2H), 4.53 (s, 2H), 3.82 (s, 3H), 3.61 (app s, 4H), 3.47 (t, *J* = 6.8 Hz, 2H), 2.31 (app oct, *J* = 3.7 Hz, 2H), 1.64–1.56 (m, 4H), 1.44–1.24 (m, 18H). ^13^C NMR (151 MHz, CDCl_3_) δ 159.2, 130.5, 129.4, 114.2 (q, *J*_*CF*_ = 256.7 Hz), 113.7, 89.4 (q, *J*_*CF*_ = 6.3 Hz), 72.9, 71.6, 70.2, 69.1, 68.3 (q, *J*_*CF*_ = 51.7 Hz), 55.2, 29.7, 29.6, 29.5, 29.5, 29.4, 29.0, 28.7, 27.2 (q, *J*_*CF*_ = 1.7 Hz), 26.1, 18.1 (q, *J*_*CF*_ = 1.6 Hz) (22 out of 26 carbon signals observed due to overlapping signals lacking distinct resonances). ^19^F NMR (565 MHz, CDCl_3_) δ -49.33 (t, *J* = 3.9 Hz). HRMS (ESI) *m/z* calculated for C_26_H_39_O₃F₃^+^ [M + Na]^+^, 479.27435 found, 479.27423.

##### 2.1.8.5 1-Methoxy-4-[2-(16,16,16-trifluoroheptadec-15-ynoxy)ethoxymethyl]benzene (29)

An oven-dried flask equipped with a magnetic stir bar was charged with CuI (1.56 g, 8.20 mmol, 1.5 equiv), K_2_CO_3_ (2.27 g, 16.4 mmol, 3.0 equiv), TMEDA (1.2 ml, 8.2 mmol, 1.5 equiv), and DMF (10 ml) under a balloon of air. The resulting blue mixture was stirred vigorously at rt for 15 min. Trifluoromethyltrimethylsilane (1.60 ml, 10.9 mmol, 2.0 equiv) was added, and the resulting reaction mixture was stirred for an additional 5 min at rt before cooling to 0°C. A solution of 1-(2-hexadec-15-ynoxyethoxymethyl)-4-methoxy-benzene (26, 2.20 g, 5.46 mmol, 1.0 equiv) and trifluoromethyltrimethylsilane (1.60 ml, 10.9 mmol, 2.0 equiv) in DMF (10 ml) was added, and the resulting reaction mixture was allowed to warm to rt and stirred vigorously for 48 h. After this time, the reaction was quenched with H_2_O and extracted with DCM. The organic phases were combined, dried over anhydrous Na_2_SO_4_, filtered, and concentrated under reduced pressure. The crude material was subsequently purified by column chromatography eluting along a gradient of 5–20% EtOAc in hexanes to yield a white solid (1.80 g, 3.82 mmol, 70% yield). ^1^H NMR (600 MHz, CDCl_3_) δ 7.29 (d, *J* = 8.4 Hz, 2H), 6.89 (d, *J* = 8.5 Hz, 2H), 4.53 (s, 2H), 3.82 (s, 3H), 3.61 (app s, 4H), 3.47 (t, *J* = 6.8 Hz, 2H), 2.31 (app oct, *J* = 3.7 Hz, 2H), 1.60 (app hex, *J* = 7.2 Hz, 4H), 1.41 (app t, *J* = 7.5 Hz, 2H), 1.37–1.24 (m, 18H). ^13^C NMR (151 MHz, CDCl_3_) δ 159.2, 130.5, 129.4, 114.2 (q, *J*_*CF*_ = 256.7 Hz), 113.8, 89.4 (q, *J*_*CF*_ = 6.2 Hz), 72.9, 71.6, 70.2, 69.1, 68.3 (q, *J*_*CF*_ = 51.9 Hz), 55.2, 29.7, 29.6, 29.6, 29.6, 29.5, 29.4, 29.0, 28.7, 27.2, 26.1, 18.1 (23 out of 27 carbon signals observed due to overlapping signals lacking distinct resonances). ^19^F NMR (565 MHz, CDCl_3_) δ -49.33 (t, *J* = 4.0 Hz). HRMS (APCI) *m/z* calculated for C_27_H_41_O₃F₃^+·^ [M]^+·^, 470.30023 found, 470.30142.

##### 2.1.8.6 2-(16,16,16-Trifluorohexadec-14-ynoxy)ethanol (30).

Synthesis was carried out according to the general PMB deprotection procedure using 1-methoxy-4-[2-(16,16,16-trifluorohexadec-14-ynoxy)ethoxymethyl]benzene (28, 900 mg, 1.97 mmol) and was purified by column chromatography eluting along a gradient of 5%–40% EtOAc in hexanes to afford a white solid (700 mg with impurities). The resulting material was carried forward without additional purification. 1H NMR (600 MHz, CDCl3) δ 3.86–3.81 (m, 2H), 3.63–3.59 (m, 2H), 3.53 (t, *J* = 6.8 Hz, 2H), 2.32 (app oct, *J* = 3.7 Hz, 2H), 1.65–1.55 (m, 4H), 1.40 (p, *J* = 7.1 Hz, 2H), 1.37–1.27 (m, 16H). 13C NMR (151 MHz, CDCl3) δ 114.2 (q, *JCF* = 255.9 Hz), 89.4 (q, *JCF* = 6.2 Hz), 71.7, 71.1, 68.3 (q, *JCF* = 51.1 Hz), 61.8, 29.6, 29.6, 29.5, 29.5, 29.4, 29.4, 29.4, 28.9, 28.7, 27.2, 26.0, 18.1. 19F NMR (565 MHz, CDCl3) δ -49.35 (t, J = 4.5 Hz).

##### 2.1.8.7 2-(17,17,17-Trifluoroheptadec-15-ynoxy)ethanol (31)

Synthesis was carried out according to the general PMB deprotection procedure using 1-methoxy-4-[2-(16,16,16-trifluoroheptadec-15-ynoxy)ethoxymethyl]benzene (29, 900 mg, 1.91 mmol) and was purified by column chromatography eluting along a gradient of 5%–40% EtOAc in hexanes to afford a white solid (640 mg, 1.82 mmol, 96% yield). 1H NMR (600 MHz, CDCl3) δ 3.74 (t, *J* = 4.6 Hz, 2H), 3.56–3.53 (m, 2H), 3.48 (t, *J* = 6.7 Hz, 2H), 2.31 (app oct, *J* = 3.6 Hz, 2H), 2.07 (s, 1H), 1.59 (h, *J* = 7.2 Hz, 4H), 1.40 (app hex, *J* = 7.4 Hz, 2H), 1.37–1.22 (m, 18H). 13C NMR (151 MHz, CDCl3) δ 114.2 (q, *JCF* = 255.9 Hz), 89.4 (q, *JCF* = 6.2 Hz), 71.7, 71.4, 68.3 (q, *JCF* = 51.8 Hz), 61.9, 29.7, 29.6, 29.6, 29.5, 29.5, 29.4, 28.9, 28.7, 27.2, 26.1, 18.1 (17 out of 19 carbon signals observed due to overlapping signals lacking distinct resonances). 19F NMR (565 MHz, CDCl3) δ -49.36 (t, *J* = 4.3 Hz). HRMS (APCI) *m/z* calculated for C_19_H_34_O_2_F₃^+^ [M + H]^+^, 351.5054 found, 351.25064.

##### 2.1.8.8 Ammonium 2-((16,16,16-trifluorohexadec-14-yn-1-yl)oxy)ethyl (R)-(((1-(6-amino-9h-purin-9-yl)propan-2-yl)oxy)methyl)phosphonate (32)

Synthesis was carried out according to general TFV coupling procedure A using 2-((16,16,16-trifluorohexadec-14-ynoxy)ethanol (30, 105 mg) and purified by column chromatography eluting along a gradient of 0%–100% 80:20:3 DCM:MeOH:NH4OH (solvent B) in DCM (solvent A). Fractions containing the desired product were collected, concentrated under reduced pressure, and then purified by reverse phase (C18) column chromatography eluting along a gradient of 10%–100% MeOH in H2O. Fractions containing the desired product were collected, concentrated under reduced pressure, stirred with 7 N ammonia in MeOH for 10 min at rt, and dried under vacuum to yield an off-white solid (69 mg, 0.11 mmol, 43% yield over 2 steps). ^1^H NMR (400 MHz, CD_3_OD) δ 8.36 (s, 1H), 8.22 (s, 1H), 4.41 (dd, *J* = 14.4, 3.0 Hz, 1H), 4.23 (dd, *J* = 14.4, 6.6 Hz, 1H), 3.99–3.86 (m, 3H), 3.76 (dd, *J* = 12.8, 9.2 Hz, 1H), 3.58–3.45 (m, 3H), 3.43–3.35 (m, 2H), 2.36 (tt, *J* = 7.6, 3.8 Hz, 2H), 1.62–1.51 (m, 2H), 1.47 (app q, *J* = 6.8 Hz, 1H), 1.39 (app t, *J* = 7.4 Hz, 1H), 1.34–1.18 (m, 18H), 1.13 (d, *J* = 6.2 Hz, 3H). ^13^C NMR (151 MHz, CD_3_OD) δ 155.4, 151.5, 149.5, 143.0, 118.1, 114.3 (q, *J*_*CF*_ = 254.9 Hz), 90.1 (q, *J*_*CF*_ = 6.3 Hz), 75.6 (d, *J*_*CP*_ = 12.8 Hz), 70.9, 70.3 (d, *J*_*CP*_ = 6.6 Hz), 67.3 (q, *J*_*CF*_ = 51.7 Hz), 64.2 (d, *J*_*CP*_ = 160 Hz), 63.7 (d, *J*_*CP*_ = 6.0 Hz), 29.4, 29.3, 29.3, 29.3, 29.2, 29.1, 28.6, 28.4, 26.9 (app d, *J*_*CF*_ = 9.2 Hz), 25.8, 17.2 (app d, *J*_*CF*_ = 1.7 Hz), 15.5 (25 out of 27 carbon signals observed due to overlapping signals lacking distinct resonances). ^19^F NMR (565 MHz, CD_3_OD) δ -50.77 (t, *J* = 3.7 Hz). ^31^P NMR (243 MHz, CD_3_OD) δ 16.31. HRMS (ESI) *m/z* calculated for C_27_H_42_O_5_N_5_F_3_P^−^ [M–H]^-^, 604.28811 found, 604.28833. LC-MS (ESI, C8, 1.0 ml/min) 50%–95% MeCN in H2O (0.1% HCO2H), 6 min, RT = 1.85 min, *m/z* = 606.4 [M + H]^+^; (ESI, C8, 0.5 ml/min) 25%–95% MeCN in H2O (0.1% HCO2H), 6 min, RT = 5.05 min, *m/z* = 606.3 [M + H]^+^.

##### 2.1.8.9 Ammonium 2-((17,17,17-trifluoroheptadec-15-yn-1-yl)oxy)ethyl (R)-(((1-(6-amino-9h-purin-9-yl)propan-2-yl)oxy)methyl)phosphonate (33)

Synthesis was carried out according to general TFV coupling procedure A using 2-((17,17,17-trifluoroheptadec-15-yneoxy)ethanol (31, 147 mg, 0.417 mmol) and purified by column chromatography eluting along a gradient of 0%–100% 80:20:3 DCM:MeOH:NH4OH (solvent B) in DCM (solvent A). Fractions containing the desired product were collected, concentrated under reduced pressure, and then purified by reverse phase (C18) column chromatography eluting along a gradient of 10%–100% MeOH in H2O. Fractions containing the desired product were collected, concentrated under reduced pressure, stirred with 7 N ammonia in MeOH for 10 min at rt, and dried under vacuum to yield an off-white solid (140 mg, 0.220 mmol, 63% yield). ^1^H NMR 400 MHz, CD_3_OD) δ 8.31 (s, 1H), 8.18 (s, 1H), 4.36 (dd, *J* = 14.4, 3.2 Hz, 1H), 4.21 (dd, *J* = 14.4, 6.6 Hz, 1H), 3.94–3.80 (m, 3H), 3.71 (dd, *J* = 12.7, 9.4 Hz, 1H), 3.53–3.42 (m, 3H), 3.35 (m, 2H), 2.37 (app oct, *J* = 3.9 Hz, 2H), 1.56 (p, *J* = 7.2 Hz, 2H), 1.45 (app t, *J* = 6.8 Hz, 2H), 1.38 (app q, *J* = 7.1 Hz, 2H), 1.34–1.18 (m, 18H), 1.14 (d, *J* = 6.2 Hz, 3H). ^13^C NMR (151 MHz, CD_3_OD) δ 155.4, 151.5, 149.5, 143.1, 118.1, 114.3 (q, *J*_*CF*_ = 254.7 Hz), 90.1 (q, *J*_*CF*_ = 6.3 Hz), 75.6 (d, *J*_*CP*_ = 12.7 Hz), 70.9, 70.3 (d, *J*_*CP*_ = 6.5 Hz), 67.3 (q, *J*_*CF*_ = 51.7 Hz), 64.2 (d, *J*_*CP*_ = 160 Hz), 63.7 (d, *J*_*CP*_ = 5.8 Hz), 29.4, 29.4, 29.3, 29.3, 29.3, 29.3, 29.3, 29.2, 29.1, 28.6, 28.4, 26.9 (app d, *J*_*CF*_ = 1.6 Hz), 25.8, 17.2 (q, *J*_*CF*_ = 1.7 Hz) 15.5. ^19^F NMR (565 MHz, CD_3_OD) δ -50.78 (t, *J* = 3.8 Hz). ^31^P NMR (243 MHz, CD_3_OD) δ 16.31. HRMS (ESI) *m/z* calculated for C_28_H_44_O_5_N_5_F_3_P^−^ [M–H]^-^, 618.30376 found, 618.30333. LC-MS (ESI, C8, 1.0 ml/min) 50%–95% MeCN in H2O (0.1% HCO2H), 6 min, RT = 2.33 min, *m/z* = 620.2 [M + H]^+^; (ESI, C8, 0.5 ml/min) 25%–95% MeCN in H2O (0.1% HCO2H), 6 min, RT = 5.40 min, *m/z* = 620.3 [M + H]^+^.

##### 2.1.8.10 2-(16,16,16-Trifluorohexadecoxy)ethanol (34)

1-Methoxy-4-[2-(16,16,16-trifluorohexadec-14-ynoxy)ethoxymethyl]benzene (30, 900 mg, 1.0 equiv), EtOAc (30 ml), palladium on carbon (10% wt, 500 mg, 3.56 mmol, 1.9 equiv) were added to a Parr shaker and hydrogenated at 15psi/1bar for 2–3 h. After this time, the heterogeneous reaction mixture was filtered over a bed of celite, and the filtrate was collected and concentrated under reduced pressure. The resulting crude product was purified by column chromatography eluting along a gradient of 5%–20% EtOAc in hexanes to afford a white solid (550 mg, 1.62 mmol, 82% yield over 2 steps). ^1^H NMR (600 MHz, CDCl_3_) δ 3.74 (dd, *J* = 5.3, 4.0 Hz, 2H), 3.56–3.53 (m, 2H), 3.48 (t, *J* = 6.7 Hz, 2H), 2.12–2.01 (m, 3H), 1.64–1.52 (m, 4H), 1.42–1.22 (m, 22H). ^13^C NMR (151 MHz, CDCl_3_) δ 127.3 (q, *J*_*CF*_ = 276.3 Hz), 71.7, 71.4, 61.9, 33.7 (q, *J*_*CF*_ = 28.2 Hz), 29.6, 29.6, 29.6, 29.5, 29.5, 29.4, 29.2, 28.7, 26.1, 21.8 (q, *J*_*CF*_ = 2.9 Hz), (15 out of 18 carbon signals observed due to overlapping signals lacking distinct resonances). ^19^F NMR (565 MHz, CDCl_3_) δ -66.44 (t, *J* = 11.0 Hz). HRMS (APCI) *m/z* calculated for C_18_H_36_O_2_F_3_^+^ [M + H]^+^, 341.26619 found, 341.26654.

##### 2.1.8.11 2-(17,17,17-Trifluoroheptadecoxy)ethanol (35)

1-Methoxy-4-[2-(16,16,16-trifluoroheptadec-15-ynoxy)ethoxymethyl]benzene (31, 900 mg, 1.91 mmol, 1.0 equiv), EtOAc (30 ml), palladium on carbon (10% wt, 500 mg, 3.56 mmol, 1.9 equiv) were added to a Parr shaker and hydrogenated at 15psi/1bar for 2–3 h. After this time, the heterogeneous reaction mixture was filtered over a bed of celite, and the filtrate was collected and concentrated under reduced pressure. The resulting crude product was purified by column chromatography eluting along a gradient of 5%–20% EtOAc in hexanes to afford a white solid (600 mg, 1.69 mmol, 88% yield). ^1^H NMR (600 MHz, CDCl_3_) δ 3.74 (t, *J* = 4.6 Hz, 2H), 3.54 (t, *J* = 4.6 Hz, 2H), 3.48 (t, *J* = 6.7 Hz, 2H), 2.07 (m, 3H), 1.64–1.52 (m, 4H), 1.41–1.21 (m, 24H). ^13^C NMR (151 MHz, CDCl_3_) δ 127.3 (q, *J*_*CF*_ = 276.4 Hz), 71.7, 71.4, 61.9, 33.7 (q, *J*_*CF*_ = 28.2 Hz), 29.7, 29.6, 29.5, 29.5, 29.4, 29.2, 28.7, 26.1, 21.8 (q, *J*_*CF*_ = 2.8 Hz) (14 out of 19 carbon signals observed due to overlapping signals lacking distinct resonances). ^19^F NMR (565 MHz, CDCl_3_) δ -66.45 (t, *J* = 11.1 Hz). HRMS (APCI) *m/z* calculated for C_19_H_38_O_2_F_3_^+^ [M + H]^+^, 355.28184 found, 355.28164.

##### 2.1.8.12 Ammonium 2-((16,16,16-trifluorohexadecyl)oxy)ethyl (R)-(((1-(6-amino-9H-purin-9-yl)propan-2-yl)oxy)methyl)phosphonate (36)

Synthesis was carried out according to general TFV coupling procedure A using 2-((17,17,17-trifluoroheptadec-15-yneoxy)ethanol (34, 100 mg, 0.293 mmol) and purified by column chromatography eluting along a gradient of 0%–100% 80:20:3 DCM:MeOH:NH4OH (solvent B) in DCM (solvent A). Fractions containing the desired product were collected, concentrated under reduced pressure, and then purified by reverse phase (C18) column chromatography eluting along a gradient of 10%–100% MeOH in H2O. Fractions containing the desired product were collected, concentrated under reduced pressure, stirred with 7 N ammonia in MeOH for 10 min at rt, and dried under vacuum to yield an off-white solid (57 mg, 0.094 mmol, 38% yield). ^1^H NMR (600 MHz, CD_3_OD) δ 8.35 (s, 1H), 8.22 (s, 1H), 4.40 (dd, *J* = 14.4, 3.2 Hz, 1H), 4.25 (dd, *J* = 14.4, 6.6 Hz, 1H), 3.98–3.87 (m, 3H), 3.75 (dd, *J* = 12.8, 9.3 Hz, 1H), 3.56–3.45 (m, 3H), 3.44–3.35 (m, 2H), 2.19–2.09 (m, 2H), 1.59–1.52 (m, 2H), 1.49 (p, *J* = 6.9 Hz, 2H), 1.40 (app t, *J* = 7.1 Hz, 2H), 1.37–1.22 (m, 20H), 1.17 (d, *J* = 6.2 Hz, 3H). ^13^C NMR (151 MHz, CD_3_OD) δ 155.8, 152.1, 149.6, 142.9, 127.5 (q, *J*_*CF*_ = 275.4 Hz), 118.2, 75.5 (d, *J*_*CP*_ = 12.7 Hz), 70.9, 70.3 (d, *J*_*CP*_ = 6.6 Hz), 64.2 (d, *J*_*CP*_ = 159.7 Hz), 63.7 (d, *J*_*CP*_ = 5.7 Hz), 33.0 (q, *J*_*CF*_ = 28.2 Hz), 29.4, 29.4, 29.3, 29.3, 29.3, 29.2, 29.2, 29.1, 29.1, 28.9, 28.4, 25.8, 21.6 (q, *J*_*CF*_ = 2.9 Hz), 15.4 (26 out of 27 carbon signals observed due to overlapping signals lacking distinct resonances). ^19^F NMR (565 MHz, CD_3_OD) δ -68.02 (t, *J* = 11.2 Hz). ^31^P NMR (243 MHz, CD_3_OD) δ 16.23. HRMS (ESI) *m/z* calculated for C_27_H_46_O_5_N_5_F_3_P^−^ [M–H]^-^, 608.31941 found, 608.31934. LC-MS (ESI, C8, 1.0 ml/min) 50%–95% MeCN in H2O (0.1% HCO2H), 6 min, RT = 2.06 min, *m/z* = 610.5 [M + H]^+^; (ESI, C8, 0.5 ml/min) 25%–95% MeCN in H2O (0.1% HCO2H), 6 min, RT = 5.24 min, m/z = 610.3 [M + H]^+^.

##### 2.1.8.13 Ammonium 2-((17,17,17-trifluoroheptadecyl)oxy)ethyl (R)-(((1-(6-amino-9h-purin-9-yl)propan-2-yl)oxy)methyl)phosphonate (37)

Synthesis was carried out according to general TFV coupling procedure A using 2-((17,17,17-trifluoroheptadec-15-yneoxy)ethanol (35, 148 mg, 0.418 mmol) and purified by column chromatography eluting along a gradient of 0%–100% 80:20:3 DCM:MeOH:NH4OH (solvent B) in DCM (solvent A). Fractions containing the desired product were collected, concentrated under reduced pressure, and then purified by reverse phase (C18) column chromatography eluting along a gradient of 10%–100% MeOH in H2O. Fractions containing the desired product were collected, concentrated under reduced pressure, stirred with 7 N ammonia in MeOH for 10 min at rt, and dried under vacuum to yield an off-white solid (160 mg, 0.250 mmol, 71% yield). ^1^H NMR (600 MHz, CD_3_OD) δ 8.35 (s, 1H), 8.22 (s, 1H), 4.40 (dd, *J* = 14.4, 3.2 Hz, 1H), 4.25 (dd, *J* = 14.4, 6.7 Hz, 1H), 3.98–3.87 (m, 3H), 3.75 (dd, *J* = 12.8, 9.4 Hz, 1H), 3.55–3.45 (m, 3H), 3.43–3.38 (m, 2H), 2.20–2.08 (m, 2H), 1.59–1.52 (m, 2H), 1.49 (app q, *J* = 6.8 Hz, 2H), 1.40 (app h, *J* = 7.1 Hz, 2H), 1.29 (d, *J* = 13.3 Hz, 22H), 1.18 (d, *J* = 6.2 Hz, 3H). ^13^C NMR (151 MHz, CD_3_OD) δ 155.5, 151.6, 149.5, 143.0, 127.5 (q, *J*_*CF*_ = 275.4 Hz), 118.1, 75.5 (d, *J*_*CP*_ = 12.7 Hz), 70.9, 70.3 (d, *J*_*CP*_ = 6.6 Hz), 64.3 (d, *J*_*CP*_ = 159.4 Hz), 63.7 (d, *J*_*CP*_ = 5.4 Hz), 33.0 (q, *J*_*CF*_ = 28.2 Hz), 31.7, 29.4, 29.4, 29.4, 29.4, 29.3, 29.2, 29.2, 29.1, 28.9, 28.4, 25.8, 21.6 (q, *J*_*CF*_ = 3.2 Hz) 15.5 (26 out of 28 carbon signals observed due to overlapping signals lacking distinct resonances). ^19^F NMR (565 MHz, CD_3_OD) δ -67.99 (t, *J* = 11.2 Hz). ^31^P NMR (243 MHz, CD_3_OD) δ 16.25. HRMS (ESI) *m/z* calculated for C_28_H_48_O_5_N_5_F_3_P^−^ [M–H]^-^, 622.33506 found, 622.33557. LC-MS (ESI, C8, 1.0 ml/min) 50%–95% MeCN in H2O (0.1% HCO2H), 6 min, RT = 2.53 min, *m/z* = 624.1 [M + H]^+^; (ESI, C8, 0.5 ml/min) 25%–95% MeCN in H2O (0.1% HCO2H), 6 min, RT = 5.50 min, *m/z* = 624.3 [M + H]^+^.

##### 2.1.8.14 2-Dodec-11-ynoxyethanol (38)

Synthesis was carried out according to the general PMB deprotection procedure using 1-(2-dodec-11-ynoxyethoxymethyl)-4-methoxy-benzene (27, 370 mg, 1.07 mmol) and was purified by column chromatography eluting along a gradient of 5%–40% EtOAc in hexanes to afford a clear oil (243 mg, 1.07 mmol, >99% yield). ^1^H NMR (400 MHz, CDCl_3_) δ 3.74–3.65 (m, 2H), 3.54–3.47 (m, 2H), 3.43 (td, *J* = 6.9, 1.0 Hz, 2H), 2.14 (app td, *J* = 7.0, 2.7 Hz, 2H), 2.07 (s, 1H), 1.91 (app td, *J* = 2.7, 0.5 Hz, 1H), 1.62–1.42 (m, 4H), 1.42–1.17 (m, 12H). HRMS (APCI) *m/z* calculated for C_14_H_27_O_2_^+^ [M + H]^+^, 227.20056, found 227.20069.

##### 2.1.8.15 2-((12-(4-(Pentafluorosulfanyl)phenyl)dodec-11-yn-1-yl)oxy)ethan-1-ol (39)

2-Dodec-11-ynoxyethanol (38, 250 mg, 1.10 mmol, 1.0 equiv) was added to a flask equipped with a magnetic stir bar and diluted with THF (3 ml). Et_3_N (1.70 ml, 9.36 mmol, 8.5 equiv) and 4-iodophenylsulfur pentafluoride (401 mg, 1.21 mmol, 1.1 equiv) were subsequently added and the reaction flask was placed under vacuum and then purged with Ar. This cycle was repeated twice more before the addition of Pd(PPh_3_)_2_Cl_2_ (155 mg, 0.220 mmol, 0.2 equiv) and CuI (21 mg, 0.11 mmol, 0.1 equiv), after which the resulting reaction was heated to 55°C and vigorously stirred at this temperature for 2 h. The reaction was cooled to rt, concentrated under reduced pressure and then purified by column chromatography eluting along a gradient of 0%–25% EtOAc in hexanes to a brown wax (320 mg, 0.747 mmol, 68% yield). ^1^H NMR (400 MHz, CDCl_3_) δ 7.66–7.61 (m, 2H), 7.42 (d, *J* = 8.6 Hz, 2H), 3.74–3.67 (m, 2H), 3.54–3.49 (m, 2H), 3.45 (t, *J* = 6.7 Hz, 2H), 2.39 (t, *J* = 7.1 Hz, 2H), 1.96 (app d, *J* = 3.7 Hz, 2H), 1.64–1.51 (m, 5H), 1.46–1.36 (m, 2H), 1.36–1.21 (m, 8H). ^13^C NMR (151 MHz, CDCl_3_) δ 152.4 (p, *J*_*CF*_ = 17.6 Hz), 131.6, 128.0, 125.9 (p, *J*_*CF*_ = 4.5 Hz), 94.2, 79.0, 71.7, 71.4, 61.9, 29.7, 29.5, 29.5, 29.5, 29.1, 28.9, 28.5, 26.1, 19.4 (18 out of 20 carbon signals observed due to overlapping signals lacking distinct resonances). ^19^F NMR (377 MHz, CDCl_3_) δ 84.38 (p, *J* = 151.4 Hz, 1 F), 62.75 (d, *J* = 150.0 Hz, 4 F). HRMS (APCI) *m/z* calculated for C_20_H_30_F_5_O_2_S^+^ [M + H]^+^, 429.18812 found, 429.18926.

##### 2.1.8.16 Ammonium [(1R)-2-(6-aminopurin-9-yl)-1-methyl-ethoxy]methyl-[2-[12-[4-(pentafluorosulfanyl)phenyl]dodec-11-ynoxy]ethoxy]phosphinate (40)

To a stirring suspension of 2-[12-[4-(pentafluorosulfanyl)phenyl]dodec-11-ynoxy]ethanol (39, 50 mg, 0.12 mmol, 1.0 equiv), TFV (34 mg, 0.12 mmol, 1.0 equiv), and 1-methylimidazole (19 μL, 0.23 mmol, 2.0 equiv) in NMP (1 ml) at rt was added TCFH (66 mg, 0.23 mmol, 2.0 equiv) followed by triethylamine (42 μl, 0.23 mmol). The mixture was stirred at rt overnight. The following morning, the reaction was quenched with NH_4_OH, and then concentrated under reduced pressure. The resulting crude material was purified by column chromatography eluting along a gradient of 0%–100% 80:20:3 DCM:MeOH:NH4OH (solvent B) in DCM (solvent A). Fractions containing the desired product were collected, concentrated under reduced pressure, and then purified by reverse phase (C18) column chromatography eluting along a gradient of 10%–100% MeOH in H2O. Fractions containing the desired product were collected, concentrated under reduced pressure, stirred with 7 N ammonia in MeOH for 10 min at rt, and dried under vacuum to yield an off-white solid (30 mg, 0.042 mmol, 36% yield). ^1^H NMR (600 MHz, CD_3_OD) δ 8.34 (s, 1H), 8.22 (s, 1H), 7.76 (d, *J* = 8.9 Hz, 2H), 7.52 (d, *J* = 8.4 Hz, 2H), 4.40 (dd, *J* = 14.5, 3.2 Hz, 1H), 4.25 (dd, *J* = 14.4, 6.7 Hz, 1H), 3.97–3.86 (m, 3H), 3.75 (dd, *J* = 12.8, 9.4 Hz, 1H), 3.56–3.43 (m, 3H), 3.43–3.35 (m, 2H), 2.45 (t, *J* = 7.1 Hz, 2H), 1.61 (p, *J* = 7.2 Hz, 2H), 1.53–1.42 (m, 4H), 1.38–1.23 (m, 10H), 1.17 (d, *J* = 6.2 Hz, 3H). ^13^C NMR (151 MHz, CD_3_OD) δ 155.8, 152.2 (p, *J*_*CF*_ = 17.3 Hz), 152.1, 149.6, 142.9, 131.4, 128.2, 125.7 (q, *J*_*CF*_ = 4.6 Hz), 118.2, 93.7, 78.5, 75.6 (d, *J*_*CP*_ = 12.8 Hz), 70.9, 70.3 (d, *J*_*CP*_ = 6.7 Hz), 64.2 (d, *J*_*CP*_ = 160.2 Hz), 63.7 (d, *J*_*CP*_ = 3.1 Hz), 63.7, 29.4, 29.3, 29.2, 28.8, 28.6, 28.2, 25.8, 18.6, 15.5 (26 out of 29 carbon signals observed due to overlapping signals lacking distinct resonances). ^19^F NMR (565 MHz, CD_3_OD) δ 82.87 (p, *J =* 148.1 Hz, 1 F), 61.28 (d, *J =* 147.9 Hz, 4 F). ^31^P NMR (243 MHz, CD_3_OD) δ 16.24. HRMS (ESI) calculated for C_29_H_40_O_5_N_5_F_5_PS^−^ [M–H]^-^, 696.24134 found, 696.24206. LC-MS (ESI, C8, 0.5 ml/min) 50%–95% MeCN in H2O (0.1% HCO2H), 6 min, RT = 2.47 min, *m/z* = 698.2 [M + H]^+^; (ESI, C8, 0.5 ml/min) 20%–100% MeCN in H2O (0.1% HCO2H), 6 min, RT = 3.90 min, *m/z* = 698.4 [M + H]^+^.

##### 2.1.8.17 2-[12-[4-(Pentafluorosulfanyl)phenyl]dodecoxy]ethanol (41)

2-((12-(4-(Pentafluorosulfanyl)phenyl)dodec-11-yn-1-yl)oxy)ethan-1-ol (39, 80 mg, 0.19 mmol, 1.0 equiv) and EtOAc (3 ml) were added to an oven-dried flask equipped with a magnetic stir bar. The solution was subsequently degassed under gentle vacuum for approximately 10 min, and then the reaction flask was purged with Ar. This cycle was repeated twice more before the addition of palladium on carbon (10% wt, 40 mg, 0.038 mmol, 0.20 equiv). Once more, the reaction flask was placed under vacuum before a final purge using a H_2_ balloon. The resulting reaction mixture was subsequently stirred vigorously under an atmosphere of H_2_ at rt overnight. After this time, the heterogeneous reaction mixture was filtered over a bed of celite, and the mother liquor was concentrated under reduced pressure. The resulting crude product was purified by column chromatography eluting along a gradient of 0–25% EtOAc in hexanes to yield a brown wax (45 mg, 0.10 mmol, 56% yield). ^1^H NMR (600 MHz, CDCl_3_) δ 7.68–7.64 (m, 2H), 7.27 (d, *J* = 8.3 Hz, 2H), 3.77–3.72 (m, 2H), 3.58–3.53 (m, 2H), 3.49 (t, *J* = 6.7 Hz, 2H), 2.66 (t, *J* = 7.7 Hz, 2H), 1.84 (s, 1H), 1.66–1.57 (m, 4H), 1.40–1.26 (m, 16H). ^13^C NMR (151 MHz, CDCl_3_) δ 151.7 (p, *J*_*CF*_ = 17.0 Hz), 147.0, 128.6, 125.8 (p, *J*_*CF*_ = 4.1 Hz), 71.7, 71.4, 61.9, 35.5, 31.1, 29.7, 29.6, 29.6, 29.5, 29.5, 29.4, 29.2, 26.1 (17 out of 20 carbon signals observed due to overlapping signals lacking distinct resonances). ^19^F NMR (377 MHz, CDCl_3_) δ 85.45 (p, *J* = 150.6 Hz, 1 F), 63.21 (d, *J* = 149.9 Hz, 4 F).

##### 2.1.8.18 Ammonium [(1R)-2-(6-aminopurin-9-yl)-1-methyl-ethoxy]methyl-[2-[12-[4-(pentafluorosulfanyl)phenyl]dodecoxy]ethoxy]phosphinate (42)

2-[12-[4-(pentafluorosulfanyl)phenyl]dodecoxy]ethanol (41, 45 mg, 0.10 mmol, 1.0 equiv) and DMF (0.21 M) were added to an oven-dried flask equipped with a magnetic stir bar under an atmosphere of Ar. TFV (30 mg, 0.10 mmol, 1.0 equiv) and 1-methylimidazole (42 μl, 0.52 mmol, 5.0 equiv) was subsequently added, followed by trisyl chloride (63 mg, 0.21 mmol, 2.0 equiv), and the resulting reaction mixture was stirred vigorously at rt for 48 h under Ar. After this time, the reaction mixture was heated to 100°C and stirred at this temperature overnight. The following day, the reaction was cooled to rt, quenched with NH_4_OH and then concentrated under reduced pressure. The resulting crude material was purified by column chromatography eluting along a gradient of 0%–100% 80:20:3 DCM:MeOH:NH4OH (solvent B) in DCM (solvent A). Fractions containing the desired product were collected, concentrated under reduced pressure, and then purified by reverse phase (C18) column chromatography eluting along a gradient of 10%–100% MeOH in H2O. Fractions containing the desired product were collected, concentrated under reduced pressure, stirred with 7 N ammonia in MeOH for 10 min at rt, and dried under vacuum to yield a brown solid (18 mg, 0.025 mmol, 24% yield). ^1^H NMR (600 MHz, CD_3_OD) δ 8.35 (s, 1H), 8.22 (s, 1H), 7.74–7.68 (m, 2H), 7.37 (d, *J =* 8.3 Hz, 2H), 4.40 (dd, *J =* 14.4, 3.2 Hz, 1H), 4.25 (dd, *J* = 14.4, 6.6 Hz, 1H), 3.98–3.87 (m, 3H), 3.75 (dd, *J* = 12.8, 9.3 Hz, 1H), 3.55–3.45 (m, 3H), 3.44–3.35 (m, 2H), 2.70 (dd, *J* = 8.7, 6.7 Hz, 2H), 1.69–1.61 (m, 2H), 1.49 (p, *J* = 6.9 Hz, 2H), 1.40–1.22 (m, 16H), 1.17 (d, *J* = 6.2 Hz, 3H). ^13^C NMR (151 MHz, CD_3_OD) δ 155.7, 152.0, 151.5 (p, *J*_*CF*_ = 16.6 Hz), 149.5, 147.4, 142.9, 128.5, 125.5 (p, *J*_*CF*_ = 4.6 Hz), 118.2, 75.5 (d, *J*_*CP*_ = 12.7 Hz), 70.9, 70.3 (d, *J*_*CP*_ = 6.6 Hz), 64.2 (d, *J*_*CP*_ = 160.9 Hz), 63.7 (d, *J*_*CP*_ = 5.7 Hz), 35.0, 30.8, 29.4, 29.3, 29.3, 29.3, 29.2, 29.2, 29.1, 28.9, 25.8, 15.4 (26 out of 29 carbon signals observed due to overlapping signals lacking distinct resonances). ^31^P NMR (243 MHz, CD_3_OD) δ 16.27. ^19^F NMR (565 MHz, CD_3_OD) δ 84.02 (p, *J* = 147.8 Hz, 1 F), 61.74 (d, *J* = 147.6 Hz, 4 F). HRMS (ESI) calculated for C_29_H_44_O_5_N_5_F_5_PS^−^ [M–H]^-^, 700.27264 found, 700.27310. LC-MS (ESI, C8, 1.0 ml/min) 60%–85% MeCN in H2O (0.1% HCO2H), 6 min, RT = 3.90 min, *m/z* = 702.4 [M + H]^+^; (ESI, C8, 0.5 ml/min) 50%–95% MeCN in H2O (0.1% HCO2H), 6 min, RT = 1.79 min, *m/z* = 702.2 [M + H]^+^.

### 2.2 Cellular toxicity and antiviral activity assays

HIV pseudoviral assays were performed as described previously (Pribut et al., 2021). Briefly, human embryonic kidney (HEK293T) cells were maintained in high glucose (25 mM), Dulbecco’s modified Eagle’s medium (DMEM) supplemented with fetal bovine serum (10%), sodium pyruvate (1 mM), l-glutamine (2 mM), HEPES (25 mM), and gentamicin (50 μg/ml). Cells were maintained in an incubator at 37°C under 5% CO_2_ under a humidified atmosphere. For both cytotoxicity and anti-pseudoviral assays, all final compounds were assessed in duplicate in ≥2 independent experiments. All prodrugs of TFV, unless otherwise specified, were formulated with human serum albumin (HSA, 5:1 compound to HSA molar ratio) and diluted with complete DMEM to 200 μM. Fifty μL of test compound 3-fold serial dilutions were prepared in 96-well culture plates, after which 50 μL of HEK293T cells (2 × 10^4^ cells/well) were added. Final prodrug concentrations for cytotoxicity determinations ranged from 1.7 nM–100 μM. A growth medium control without test compound was included as an indicator of 100% cell viability (no cytotoxicity). 96-Well plates were incubated for 48 h at 37°C under 5% CO_2_ under a humidified atmosphere. Cytotoxicity was assessed by quantifying cell viability using the CellTiter 96 Aqueous One Solution Cell Proliferation Assay (Promega, Madison, WI) or resazurin sodium salt (cat# R7017, Merck, Darmstadt, Germany) (Riss, 2013). The concentration of prodrugs that kills 50% of cultured cells (CC_50_) was calculated using Microsoft Excel.

Antiviral activity was subsequently assessed using a single-cycle, non-replicating, envelope-deleted HIV pseudoviral system (Parry et al., 2009; Gupta et al., 2010). The assay relies on expression of firefly luciferase by HEK293T cells through infection with VSV pseudotype HIV-like viral particles loaded with the firefly luciferase transcript. The expression of luciferase in HEK293T cells is directly proportional to the level of infection by the pseudoviral particles. To evaluate the anti-HIV activity, 3-fold serial dilutions of all final compounds were prepared in 50 μL over the non-cytotoxic concentration range (as determined from the cytotoxicity screens described above) in 96-well plates. HEK293T cells (2 × 10^4^ cells/well) and pseudoviral particles, standardized to produce a luminescence signal of 1 × 10^5^ relative light units in the growth medium-only control, were combined, and 50 μl of the resulting solution was added to the wells containing serially diluted prodrugs. A growth medium control without prodrug was included as a representation of 100% viral activity (no inhibition). 96-Well plates were incubated for 48 h at 37°C under 5% CO_2_ under a humidified atmosphere. Luciferase expression was subsequently quantified by adding 100 μL of the Bright-Glo Luciferase Assay substrate (Promega, Madison, WI) to each well. After incubation at rt for 3 min, luminescence was quantified on a GloMax Explorer Multimode Microplate Reader (Promega, Madison, WI). The concentration of each compound required to inhibit viral activity by 50% (IC_50_) was calculated using Microsoft Excel. Concentration-response curves are reported in Supplementary Figures S1–S6 in Supporting Information.

### 2.3 Metabolic stability assays

Metabolic stability assays using human liver microsomes (HLM) were performed as described previously (Pribut et al., 2021). Briefly, 20 mg/ml HLM (Xenotech), 10 mM stock solutions (DI H_2_O) of NADPH (Sigma-Aldrich), and 10 mM stock solutions (MeOH) of prodrug and positive control verapamil were utilized for metabolic stability experiments. Control and test compound stock solutions were further diluted to 500 μM working solutions with potassium phosphate buffer (100 mM, pH 7.4). Final MeOH concentrations were <0.2%. HLM were prepared in 1.5 ml of Eppendorf tubes with final volumes of 1.1 ml to accommodate for duplicate experiments. Final reaction mixtures contained phosphate buffer (928 μl), HLM (55 μl), and prodrug (6.6 μl of 500 μM working solutions) for a final concentration of 3 μM. Metabolic reactions were initiated with 10 mM NADPH (110 μl). Aliquots (100 μl) were removed from each reaction mixture in duplicate at several time points between 0 and 120 min, quenching with 100 μL of cold MeOH, containing 2 μM internal standard (ISTD) 7-ethoxy-*d*_*5*_-coumarin. Quenched aliquots were centrifuged at 12,000 g for 5 min, and supernatants were transferred to LC-MS vials. LC-MS/MS (Agilent G6460C QQQ MS coupled with an Infinity II 1260 HPLC) analysis provided an area under the m/z curve (AUC) for each compound at each time point. Precursor and product ion detection was performed with Agilent Jet Stream electrospray positive ionization (ESI+) in multiple reaction monitoring (MRM) mode. All MRM transitions, fragmentor voltages, and collision energies for individual compounds are provided in Supplementary Table S2. Other standard MS conditions include dwell time of 100 m, gas flow of 10 L/min, nebulizer pressure of 45 psi, and delta EMV of 200 V. Reverse-phase HPLC separation for each compound was achieved with an Agilent InfinityLab Poroshell EC-C8 (2.1 × 50 mm, 2.7 μm) column maintained at 40°C. The mobile phase used during analyses consisted of either MeOH-H_2_O (0.1% formic acid) or MeCN-H_2_O (0.1% formic acid) at a flow rate of 0.5 ml/min. The resulting data were then normalized with respect to ISTD AUCs and processed using Agilent 6,460 Quantitative Analysis software. Negative controls (no NADPH) were additionally included, using a final reaction volume of 150 μl and terminating the reaction at the final 120 min time point. Each data point was analyzed in duplicate, and the resulting means were normalized to the 0 min data, representing 100% test compound remaining (0% metabolism). T_1/2_ values were calculated using linear regression of ln (% remaining) versus time plots as previously described. Detailed results are reported in Supplementary Table S1.

## 3 Results and discussion

Since the common active metabolite of all TFV lipid prodrugs is TFV-DP, IC_50_ values in pseudoviral assays reflect the efficiency of cellular entry and prodrug processing to TFV-DP. Complementarily, CC50 values reflect the cytotoxicity of each prodrug and the corresponding ensemble of metabolites. Accordingly, low antiviral IC_50_ and high cytotoxic CC_50_ concentrations lead to large therapeutic indices indicative of safe and specific inhibition of HIV-RT. We previously demonstrated that lipid prodrugs of TFV featuring 18 and 20 atom lipid chain lengths were more potent in HIV pseudoviral assays than those with 15 atom chain lengths (Pribut et al., 2021). Prodrugs with 20 atom chain lengths additionally proved more stable in HLM than those with 15 and 18 atom chain lengths. Furthermore, ether-linked lipid prodrugs (e.g., TXL) exhibited better antiviral activity than methylene-linked congeners (e.g., arachidyl TFV). Finally -CF_3_, -C≡C-CF_3_, and -C≡C-Si(CH_3_)_3_ lipid termini demonstrated substantially improved metabolic stability in HLM relative to the unfunctionalized terminus of TXL. Although -CF_3_ and -C≡C-Si(CH_3_)_3_ TXL analogues showed significantly improved mouse plasma and liver PK properties *in vivo*, the -CF_3_ derivative persisted in plasma with a 3-fold longer t_1/2_ value than TXL, -C≡C-CF_3_, and -C≡C-Si(CH_3_)_3_ analogues.

With this preliminary data, we were eager to further optimize the lipid chain length, the oxyalkyl linker unit, and the metabolically stable terminal motif. Perhaps unsurprisingly, optimization of lipid prodrug antiviral activity relies on the delicate balance between cell membrane permeability and aqueous solubility. Prodrugs that are too hydrophobic are not soluble enough in physiological systems, whereas prodrugs that are too hydrophilic are not permeable enough to access intracellular environments. Accordingly, desired pharmacodynamic effects are constrained by these physicochemical limits. All previously synthesized lipid prodrugs required formulation with fatty acid-binding human serum albumin (HSA) to facilitate prodrug solubility in cell-based pseudoviral assays. This formulation mimics the physiological scenario where lipid prodrugs of TFV (1) are highly protein bound in the plasma (Pribut et al., 2021) and (2) require dissociation from HSA and subsequent association with plasma membranes of HIV-infected cells to exert antiviral activity. High affinity binding of these lipid prodrugs to plasma proteins like HSA, in principle, can create a circulating reservoir of TFV prodrugs that slowly equilibrate across membranes and distribute broadly around the body. However, we previously observed that ω-unfunctionalized lipid prodrugs of TFV featuring 22 and 24 atom chain lengths were not soluble in HSA formulations or in growth media (Miller, 2020). As a consequence, 22 atom lipid chain lengths were defined as the lipophilicity limit, but the hydrophilicity limit was yet to be defined.

**SCHEME 1:**
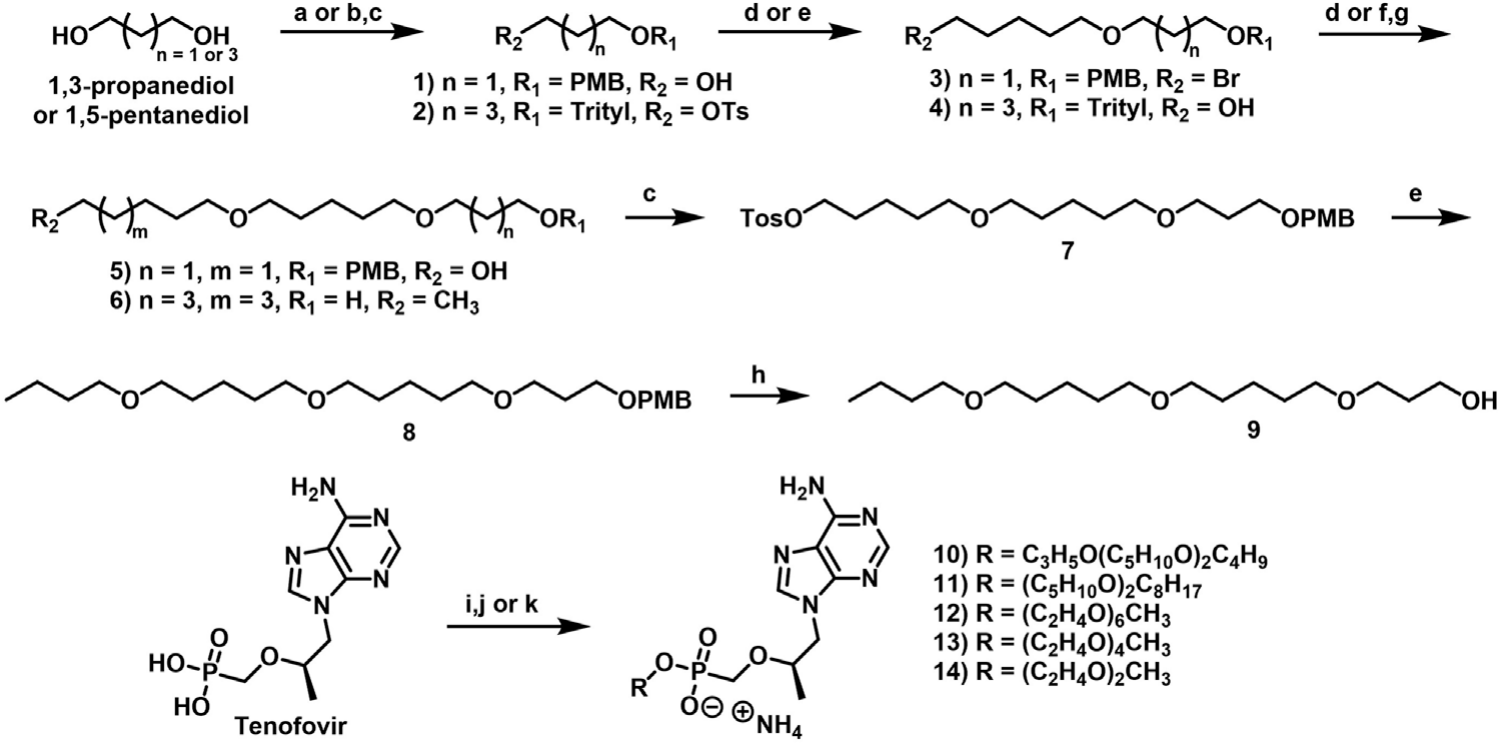
Reagents and conditions: **(A)** PMBCI, KOH, DMSO, 0°C to rt, overnight, 68%; **(B)** Ph3CCI, Et3N, DMAP, DCM, 50°C, 4.5 h, 92%; **(C)** p-TsCI, Et3N, DMAP, DCM, rt, overnight, 70%–82%; **(D)** 1,5-dibromopentane or 1,5-pentanediol, TBAB, NaOH (aq), THF, 75°C, overnight, 44%–50%; **(E)** 1,5-pentanediol or 1-butanol, NaH, DMF, 0°C to rt, overnight, 73%; **(F)** octylbromide, TBAHS, NaOH aq), toluene, 65°C, overnight, 60%; **(G)** 80% AcOH (aq), 60°C, 2 h, 67%; **(H)** CAN, Me0H/H_2_0, 0°C to rt, 3 h, 61%; **(I)** 9, DCC, Et3N, DMAP, NMP, 100°C, overnight, 25%; U) N,N-dimethylformamide dimethyl acetal, DMF, rt, 3 h; then 6, CCl3CN, pyridine, 80°C, µwave, 1.5 h, 38%; **(K)** 2-[2-[2-(2- methoxyethoxy)ethoxy]ethoxy]ethanol, 2-[2-[2-(2-methoxyethoxy)ethoxy]ethoxy]ethanol or diethylene glycol monomethyl ether, EDCHCI, Et3N, DMAP, DMF or MeCN, 90°C–105°C, overnight, 20%–33%.

To define this hydrophilicity limit, we designed a small series of TFV prodrugs featuring increasing oxygen content in the lipid chain. We hypothesized that we could identify a prodrug motif to improve aqueous solubility while maintaining antiviral potency. To test this hypothesis, we aimed to synthesize and evaluate TXL analogues featuring additional oxygen atoms spaced 5 methylene units apart, as well as a small series of polyethylene glycol (PEG)-type derivatives. While PEGylated lipids were commercially available, lipids featuring oxygen atoms 5 methylene units apart were synthesized through a series of protection, etherification and deprotection steps as illustrated in Scheme 1. First, 1,3-propanediol or 1,5-pentanediol were asymmetrically functionalized with a PMB- or trityl group respectively under basic conditions to afford intermediates 1 and 5-trityloxypentan-1-ol. The primary alcohol of 5-trityloxypentan-1-ol was then activated to the corresponding tosylate 2 before undergoing an etherification reaction with 1,5-pentanediol in the presence of NaH to give intermediate 4. Finally, a Williamson etherification reaction under phase transfer conditions between 4 and octylbromide, followed by AcOH-mediated removal of the trityl group afforded the desired synthon 6. On the other hand, two sequential Williamson etherification reactions were carried out between 1 and 1,5-dibromopentane, and then between resultant ether 3 and 1,5-pentanediol under phase transfer conditions to give 5 in 31% yield over two steps. Subsequent conversion of the terminal alcohol of 5 to the corresponding tosylate 7 was followed by a final etherification reaction between 7 and 1-butanol in the presence of NaH to afford 8, and then oxidative removal of the PMB group using CAN to furnish desired synthon 9. For the synthesis of TXL analogues featuring increasing oxygen content in the lipid chain, synthons 6 and 9, as well as three PEGylated lipids were coupled to TFV using one of three different coupling conditions, namely with DCC or EDC in the presence of Et_3_N and DMAP, or utilizing a µwave-assisted, CCl_3_CN-mediated acid coupling reaction in the presence of pyridine. These conditions afforded TXL analogues 10 and 11, which feature lipids with oxygen atoms spaced 5 methylene units apart, as well as PEGylated TXL analogues 12–14 of varying chain length.

As described previously (Pribut et al., 2021), preliminary pseudoviral assay results demonstrated a strong preference for ether-linked prodrugs (TXL, IC_50_ = 18 nM) compared to the methylene-linked congeners (arachidyl TFV, IC_50_ = 127 nM). This change in antiviral activity, coupled with differences in cytotoxicity and therapeutic index, focused our initial attention on prodrugs featuring ether linkages (Table 1). Quite interestingly, adding two additional oxygen atoms to the lipid chain of TXL (10) completely abrogated antiviral activity. Removing one of these oxygen atoms, albeit while shifting positions (11), led to comparatively improved potency, albeit in the low μM range. These results suggested that the number of the oxygen atoms in the lipid chain was an important determinant of antiviral activity. Compound 11 also proved less stable in HLM relative to TXL, but it is unclear whether the extra oxygen itself or the position of the oxygen atoms is the predominant driver of this result. Increasing the oxygen content even further to incorporate six (12), four (13), and two (14) PEG units observationally increased aqueous solubility. While this relative hydrophilicity obviated the need for HSA formulations, these prodrugs were evaluated for antiviral activity with and without HSA formulation to enable direct comparison to more hydrophobic prodrugs. Relative to TXL, increases in solubility were accompanied overall by dramatic reductions in antiviral activity. These results establish a single oxygen atom in the lipid chain as the hydrophilicity limit of antiviral activity. It is further notable that this class of lipid prodrugs demonstrates robust selectivity over cytochrome P450 (CYP) isoforms 2D6 and 3A4, mitigating potential concerns for drug-drug interactions in the clinic (Obach et al., 2006).

**TABLE 1:** *In vitro* activity profiles of lipid prodrugs of TFV containing increasing oxygen content. HIV and cytotoxicity results represent data generated (a) with compounds formulated as HSA complexes in 5:1 M ratios or (b) using DMSO stock solutions. Nd, not determined.

Compound ID	Lipid motif (R)	Linear atom #	HLM t112 (min)	HIV IC50 (µM)	St. Dev. (µM)	CCso (µM)	St. Dev (µM)	Therapeutic index	
TXL	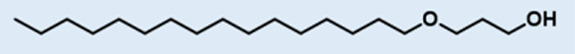	20	42	0.018a	o.01oa	97.6a	2.68a	5,420a	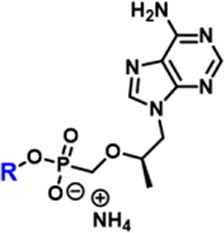
10	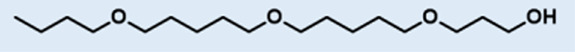	20	nd	>32a	—	>1ooa	—	—	
11	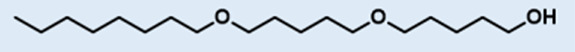	20	12	4.osa	0.597a	>1ooa	—	>25a	
12	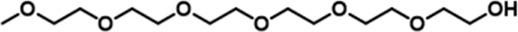	19	nd	31.2b	6.94b	>100b	—	>3b	
				34.6a	8.75a	>1ooa		>3a	
13	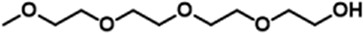	13	nd	26.7b	0.738b	>100b	—	>4b	
				23.4a	6.23a	>1ooa		>4a	
14	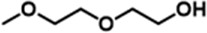	7	nd	21.4b	5.90b	>100b	—	>5b	
				19.4a	3.26a	>1ooa		>5a	

**SCHEME 2:**
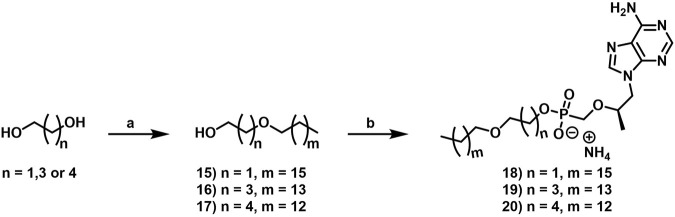
Reagents and conditions: **(A)** 1-bromoheptadecane, 1- bromopentadecane or 1-bromotetradecane, NaH, DMF, 0°C to rt, overnight, 25%–79%; **(B)** TFV, trisyl chloride, pyridine, rt, 48 h, 24%–54%.

Based on these results and the structural constraints described above, the upper limit of lipophilicity was defined as lipid chains featuring <22 atoms with one heteroatom, whereas the upper limit of hydrophilicity was defined as lipid chains with >18 atoms and a single oxygen atom. Although this is a tight window, the pseudoviral assay results outlined in Table 1 demonstrate that antiviral activity is somewhat dependent on the number and/or the position of the oxygen atom(s) in the lipid chain. Accordingly, we further explored this structure-activity relationship by varying the position of a single oxygen atom in the lipid chain. Specifically, we synthesized and evaluated a small “oxygen walk” series of lipid prodrugs featuring oxyethyl, oxybutyl, and oxypentyl linkers, as illustrated in Scheme 2. Synthesis was initiated through a Williamson etherification reaction, in the presence of NaH, between commercially available ethylene glycol, 1,4-butanediol or 1,5-pentanediol and an appropriate alkyl bromide to afford synthons 15–17. Once in hand, subsequent coupling of these ether-containing lipids to TFV was carried out using the condensing reagent trisyl chloride with pyridine as the solvent to furnish TXL analogues 18–20.

As the preliminary data reported in Table 1 suggested, antiviral activity was indeed dependent upon the position of the single oxygen atom in the lipid chain (Table 2). Interestingly, antiviral activity improved with decreasing distance between the phosphorus atom of TFV and the lipid oxygen atom. The pseudoviral assay results described in Table 2 point unequivocally to the oxyethyl linker as optimal for antiviral activity, with TXL analogue 18 demonstrating a 17-fold boost in potency relative to analogue 20 featuring the oxypentyl linker. Although HLM stability appears to peak for oxybutyl-containing analogue 19, the potency boost associated with oxyethyl-linked compound 18 outweighs the potentially insignificant modest drop in HLM stability. It is additionally notable that TXL analogue 20 is a matched molecular pair of TXL analogue 11 (Table 1). These prodrugs are differentiated only by the presence or absence of an additional oxygen atom in the oxypentyl-linked lipid chain, and yet, a substantial decay (78-fold) of antiviral activity with inclusion of the second oxygen atom was observed. This aligns well with the comparison between oxypropyl-linked matched molecular pairs TXL and TXL analogue 10, where the incorporation of two additional oxygen atoms results in >1,800-fold loss of antiviral activity. Although we can conclude that the number of oxygen atoms in the lipid chain, as well as the relative positions of these oxygen atoms, are both important determinants of antiviral activity, potency is most dramatically affected by incorporation of additional oxygen atoms, according to the data reported in Table 1 and Table 2.

**TABLE 2:** *In vitro* activity profiles of lipid prodrugs of TFV featuring increasing distances between phosphorus and oxygen. All HIV and cytotoxicity results compounds formulated as HSA complexes in 5: 1 M ratios. Nd = not determined.

Compound ID	Lipid motif (R)	Linear atom #	HLM t_1/2_ (min)	HIV IC_50_ (µM)	St. Dev. (µM)	CC_50_(µM)	St. Dev. (µM)	Therapeutic index	
18	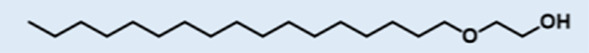	20	35	0.003	0.001	69.6	1.31	23,200	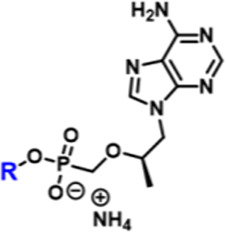
TXL	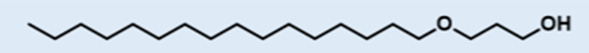	20	42	0.018	0.010	97.6^a^	2.68	5,420	
19	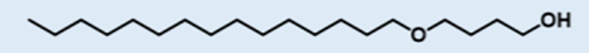	20	48	0.030	0.014	>100^a^	—	>3,330	
20	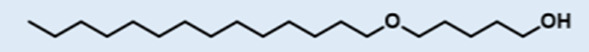	20	31	0.052	0.03	>100^b^	—	>1,920	

**SCHEME 3:**
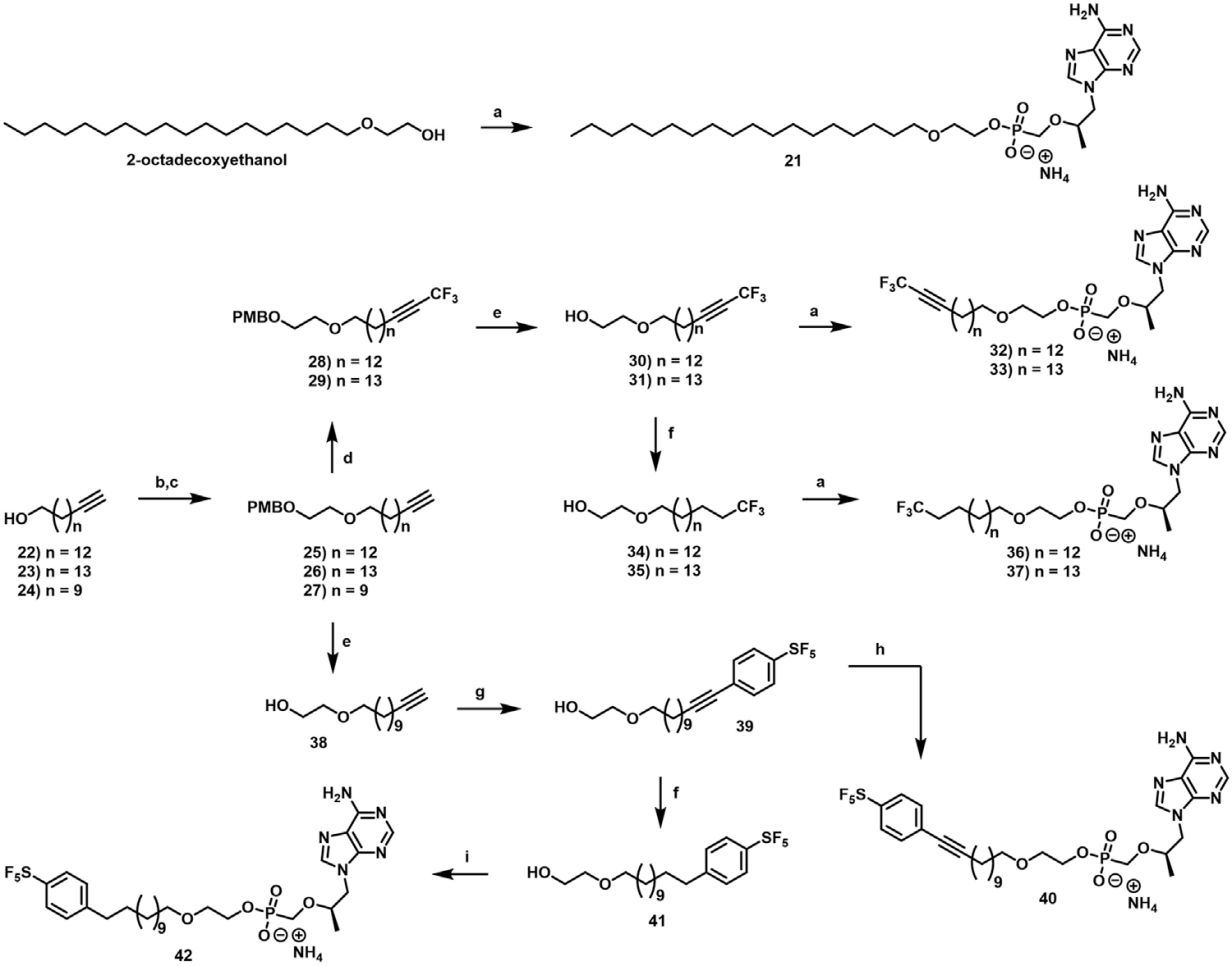
Reagents and conditions: **(A)** TFV, DCC, Et3N or DMAP, NMP, 100°C, overnight, 38%–71%; **(B)** p-TsCI, pyridine, DCM, rt, overnight, 84%–91%; **(C)** 2-[(4-methoxyphenyl)methoxy]ethanol, NaH, DMF, 0°C to rt, overnight, 68%–89%; **(D)** CF_3_Si(CH3h, Cul, K2C03, TMEDA, DMF, rt, 48 h, 70%–85%; **(E)** CAN, MeOH/H_2_0, 0°C to rt, 3 h, 96%; **(F)** 10% Pd/C, H2, EtOAc, rt, 2–3 h, 82%–88%; **(G)** 4-iodophenylsulfurpentafluoride, Pd(PPh3hC12, Cul, Et3N, THF, 55°C, 2 h, 68%; **(H)** TFV, TCFH, 1-methylimidazole, Et3N, NMP, rt, overnight, 36%; **(I)** TFV, trisyl chloride, 1-methylimidazole, DMF, rt to 100°C, 48 h, 24%.

As a consequence of the superior antiviral activity and similar HLM stability of oxyethyl-linked TXL analogue 18 compared to oxypropyl-linked TXL, metabolically stable lipid termini from previous work identified to resist CYP-mediated ω-oxidation *in vitro* and *in vivo*, as well as some novel unexplored lipid termini, were installed onto oxyethyl-linked lipid prodrug scaffolds. Due to high C-F bond dissociation energy and significant potential to electronically disfavor undesired metabolism by CYP ω-oxidases, fluorine atom installation proved to be an effective strategy to limit ω-oxidation and maximize HLM stability (Pribut et al., 2021). To complement the -CF_3_-containing lipid termini, we hypothesized that the pentafluorosulfanyl (SF_5_) motif could confer similar pharmacological profiles. The SF_5_ group has found increasing utility in medicinal chemistry campaigns (Jose et al., 2022) and is largely accepted as a bioisostere of halogens, CF_3_, and *t*-Bu groups (Sowaileh et al., 2017). SF_5_ groups impart distinct properties onto small molecules as a consequence of their octahedral geometries, unique sizes (*t*-Bu > SF_5_>CF_3_), lipophilicity, and metabolic stability. While it was not our intention to exhaustively explore these terminal groups in the context of refined lipid chain lengths (i.e., 19–21 atoms), we intentionally designed in matched molecular pairs to efficiently explore space between 19 and 21 atom lipid motifs. For the synthesis of TXL analogue 21 (Scheme 3), commercially available 2-octadecoxyethnaol was coupled to TFV in the presence of DCC and DMAP to afford 21 in a moderate yield of 50%. Synthesis of TFV lipid prodrugs featuring the CF_3_-lipid terminus was initiated using alkyne intermediates 22–24, which were either commercially available or prepared according to previously reported procedures (Pribut et al., 2021). The terminal hydroxyl groups of 22–24 were first converted to good leaving groups by way of tosylation and then reacted with mono-PMB protected ethylene glycol to furnish ethers 25–27. Synthons 25 and 26 then underwent a copper catalyzed oxidative trifluoromethylation reaction (Chu and Qing, 2010) of the terminal alkyne to afford CF_3_ acetylenyl precursors 28 and 29, which were then subjected to oxidative removal of the PMB group using CAN to give intermediates 30 and 31. Synthons 30 and 31 were subsequently coupled to TFV using previously described DCC-mediated acid coupling conditions furnishing TXL analogues 32 and 33, or were hydrogenated in a Parr shaker in the presence of palladium on carbon to afford 34 and 35 before coupling to TFV to give TXL analogues 36 and 37. For the synthesis of TXL analogues featuring the SF_5_-lipid terminus, following CAN-mediated oxidative removal of the PMB group of 27, synthon 38 was subjected to Sonogashira coupling conditions with *p*-iodophenylsulfurpentafluoride in the presence of Pd(PPh_3_)_2_Cl_2_, CuI and Et_3_N to give 39 in 68% yield. As before, precursor 39 was either coupled directly to TFV in the presence of TCFH-NMI (generated *in situ*) to furnish TXL analogue 40, or underwent hydrogenation with palladium on carbon to give the corresponding saturated intermediate 41 which was coupled to TFV using trisyl chloride to afford TXL analogue 42.

As outlined in Table 3, all prodrugs in this series demonstrated excellent antiviral activity and therapeutic indices. In addition, most of these compounds exhibited robust stability in HLM, with the notable exception of heptadecyloxyethyl derivative TXL analogue 18. Interestingly octadecyloxyethyl analogue 21 exhibited improved stability in HLM despite the lack of ω-oxidation-resistant terminal groups. In contrast to this interesting difference between the metabolic stability of 20 atom versus 21 atom lipid chain lengths, HLM stabilities of ω-functionalized lipid prodrugs featuring 19 (32 and 36) and 20 (33 and 37) atom chain lengths were indistinguishable in this experimental system. Comparison of the metabolic stability of matched molecular pairs containing lipid prodrug motifs of 19, 20, and 21 atoms will be the subject of future investigation. Notably, the small intestine and the gut-associated lymphoid tissue (GALT) are additional potential sites of lipid prodrug ω-oxidation. Accordingly, future experiments will involve both human hepatocytes, as well as caco-2 cells. Finally, the small differences in antiviral activity between TXL analogue 18 and the terminally functionalized lipid prodrugs in Table 3 demonstrates that structurally diverse metabolically stable lipid termini are well-tolerated in this HIV pseudoviral system. The extent to which this structural diversity can be pushed is an important consideration for further investigation.

**TABLE 3:** *In vitro* activity profiles of oxyethyl- linked lipid prodrugs of TFV featuring chain length tweaks and metabolically stable lipid termini. All HIV and cytotoxicity results represent data generated with compounds formulated as HSA complexes in 5:1 M ratios. Nd = not determined.

Compound ID	Lipid motif (R)	Linear atom #	HLM t112 (min)	HIV IC50 (µM)	St. Dev. (µM)	CCso (µM)	St. Dev (µM)	Therapeutic index	
18	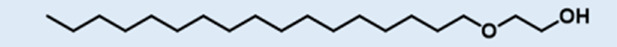	20	35	0.003	0.001	69.6	1.31	23,200	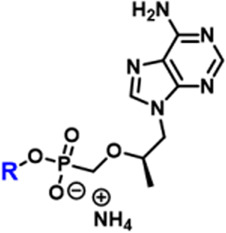
21	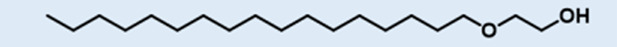	21	105	0.003	0.0006	53.8	2.83	17,900	
32	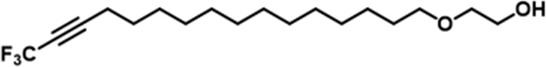	19	>120	0.016	0.0085	>100	—	>6,250	
33	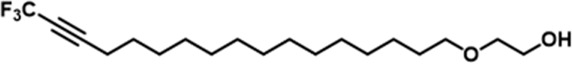	20	>120	0.0106	0.0016	>100	—	>9,430	
36	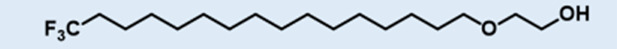	19	>120	0.0088	0.0008	>100	—	>11,400	
37	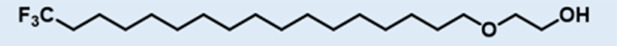	20	>120	0.0126	0.0017	85.1	13.3	6,750	
40	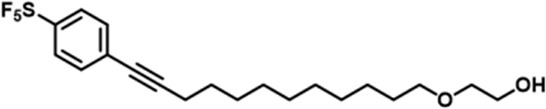	20	>120	0.0146	0.0023	>100	—	>6, 850	
42	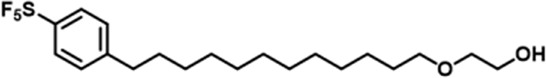	20	>120	0.0254	0.0146	>100	—	>3,940	

In summary, we designed and synthesized three series of novel lipid prodrugs of TFV featuring additional oxygen atoms in the lipid chain (Table 1), a single oxygen atom positional walk (Table 2), and new metabolically stable terminal groups with oxyethyl linkers and refined lipid chain length (Table 3). Each of these novel prodrugs were evaluated for antiviral activity in HIV pseudoviral assays and for metabolic stability in HLM. Experiments with results highlighted in Table 1 demonstrated that a single oxygen atom in the lipid chain was optimal for antiviral activity. Results outlined in Table 2 highlight the optimal antiviral activity and relatively uncompromised HLM stability of oxyethyl-linked TFV prodrugs. Furthermore, results reported in Table 3 describe that 19, 20, and 21 atom lipid chain lengths define the limit of optimal antiviral activity and metabolic stability within this series. While detailed intracellular metabolism experiments and in vivo pharmacokinetic studies are critical components of our future plans, these results also highlight SF_5_ as a complementary tool to CF_3_ and Si(CH_3_)_3_ in the growing repertoire of metabolically inert lipid termini. In conclusion, small modifications to alter the metabolic soft spots of nucleoside and nucleotide lipid prodrugs can drive dramatic improvements in PK profiles and enable compounds that are otherwise undevelopable as potential therapeutic agents to treat human disease.

## Data Availability

The original contributions presented in the study are included in the article/Supplementary Materials, further inquiries can be directed to the corresponding author.
