# Supplementary material for: Expanding the toolbox of metabolically stable lipid prodrug strategies
Source: Front Pharmacol. 2023 Jan 6;13:1083284. doi: 10.3389/fphar.2022.1083284 (PMC9852841; doi:10.3389/fphar.2022.1083284)
Supplement: Supplementary file 1 [file Presentation1.PPTX]

## Slide 1
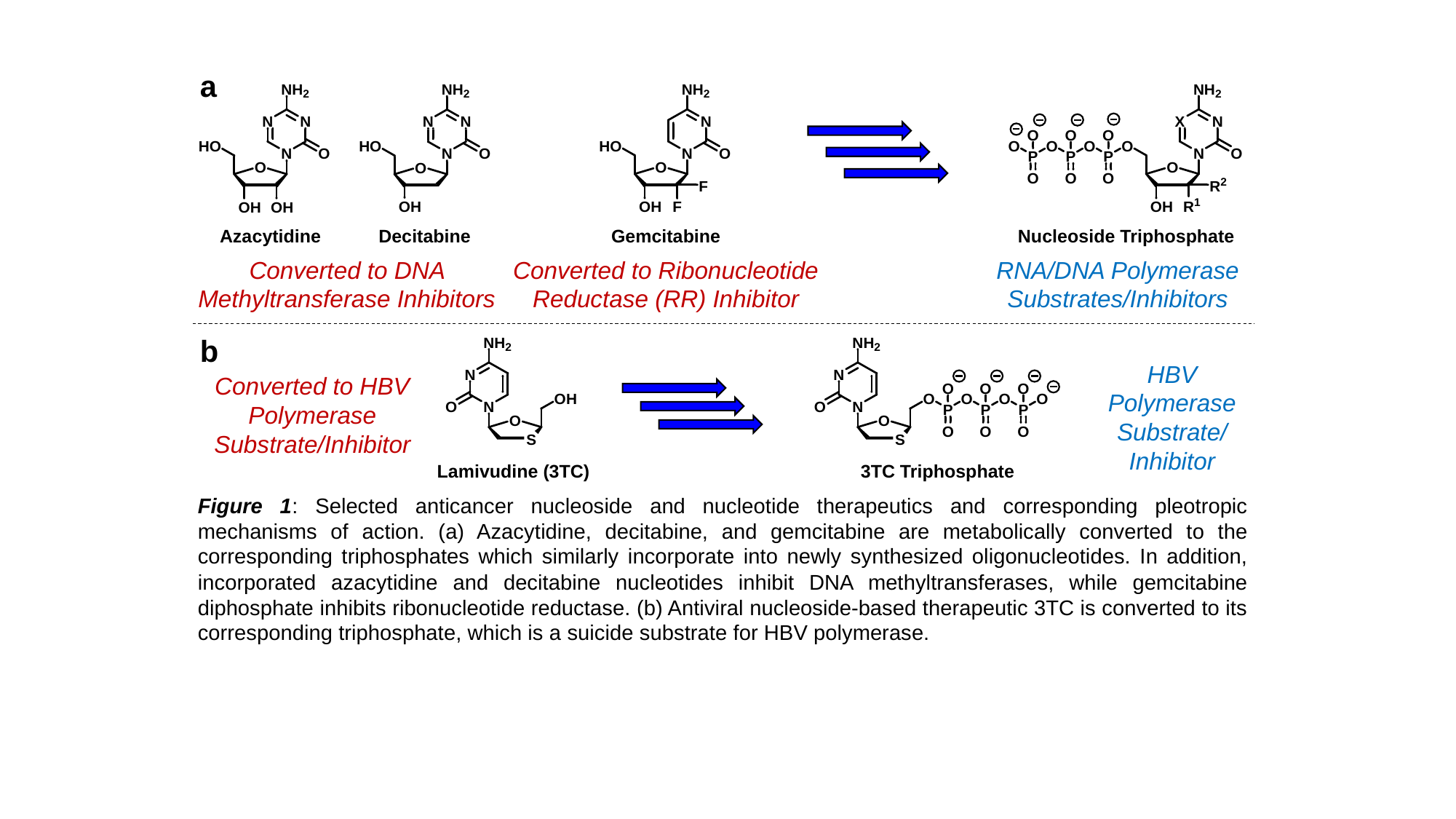

a
Azacytidine
Decitabine
Gemcitabine
Nucleoside Triphosphate
Converted to DNA Methyltransferase Inhibitors
Converted to Ribonucleotide Reductase (RR) Inhibitor
RNA/DNA Polymerase Substrates/Inhibitors
b
Lamivudine (3TC)
3TC Triphosphate
HBV Polymerase Substrate/
Inhibitor
Converted to HBV Polymerase Substrate/Inhibitor
Figure 1: Selected anticancer nucleoside and nucleotide therapeutics and corresponding pleotropic mechanisms of action. (a) Azacytidine, decitabine, and gemcitabine are metabolically converted to the corresponding triphosphates which similarly incorporate into newly synthesized oligonucleotides. In addition, incorporated azacytidine and decitabine nucleotides inhibit DNA methyltransferases, while gemcitabine diphosphate inhibits ribonucleotide reductase. (b) Antiviral nucleoside-based therapeutic 3TC is converted to its corresponding triphosphate, which is a suicide substrate for HBV polymerase.

## Slide 2
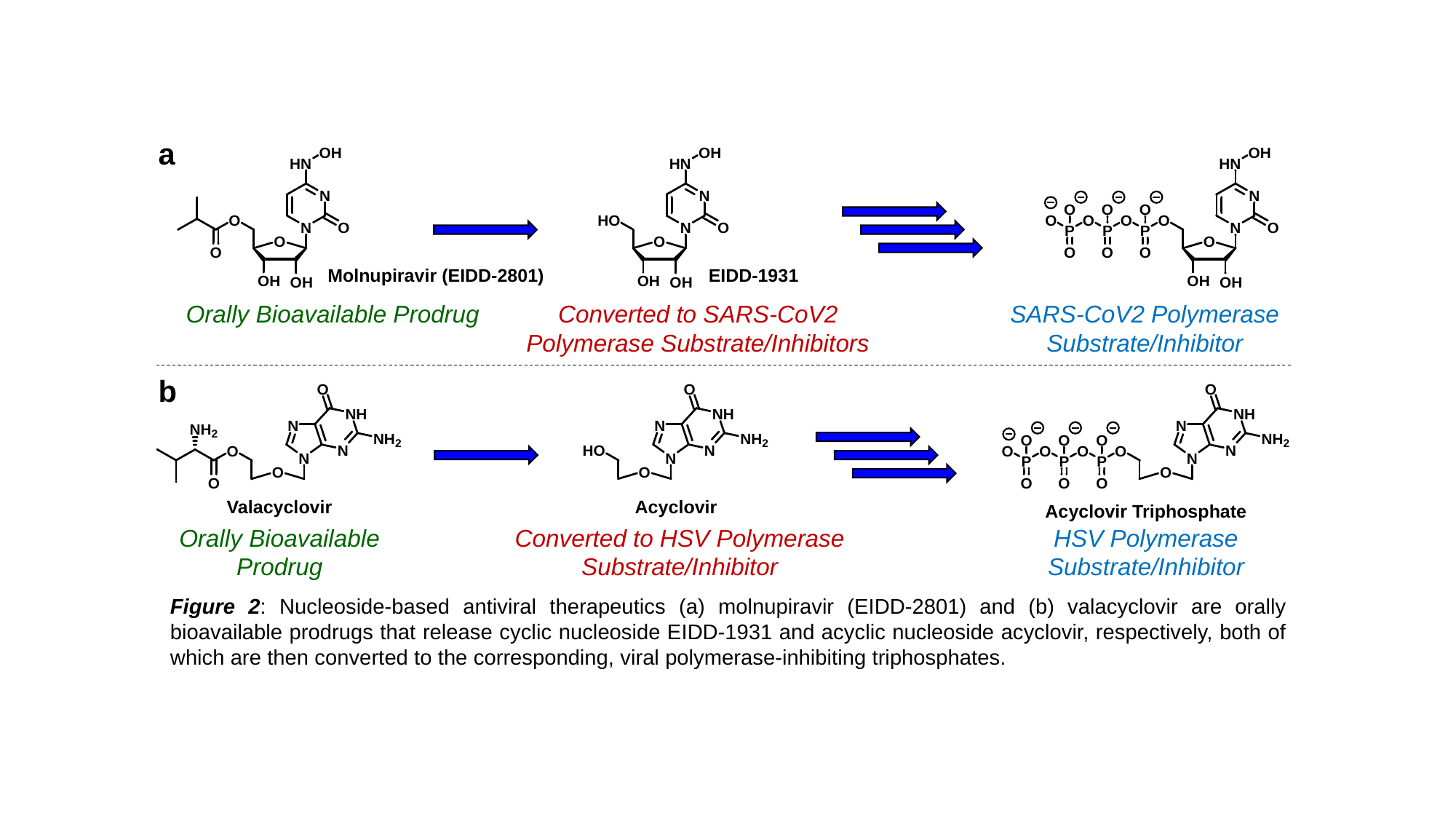

a
Molnupiravir (EIDD-2801)
EIDD-1931
Orally Bioavailable Prodrug
Converted to SARS-CoV2 Polymerase Substrate/Inhibitors
SARS-CoV2 Polymerase Substrate/Inhibitor
b
Valacyclovir
Acyclovir
Acyclovir Triphosphate
Orally Bioavailable Prodrug
Converted to HSV Polymerase Substrate/Inhibitor
HSV Polymerase Substrate/Inhibitor
Figure 2: Nucleoside-based antiviral therapeutics (a) molnupiravir (EIDD-2801) and (b) valacyclovir are orally bioavailable prodrugs that release cyclic nucleoside EIDD-1931 and acyclic nucleoside acyclovir, respectively, both of which are then converted to the corresponding, viral polymerase-inhibiting triphosphates.

## Slide 3
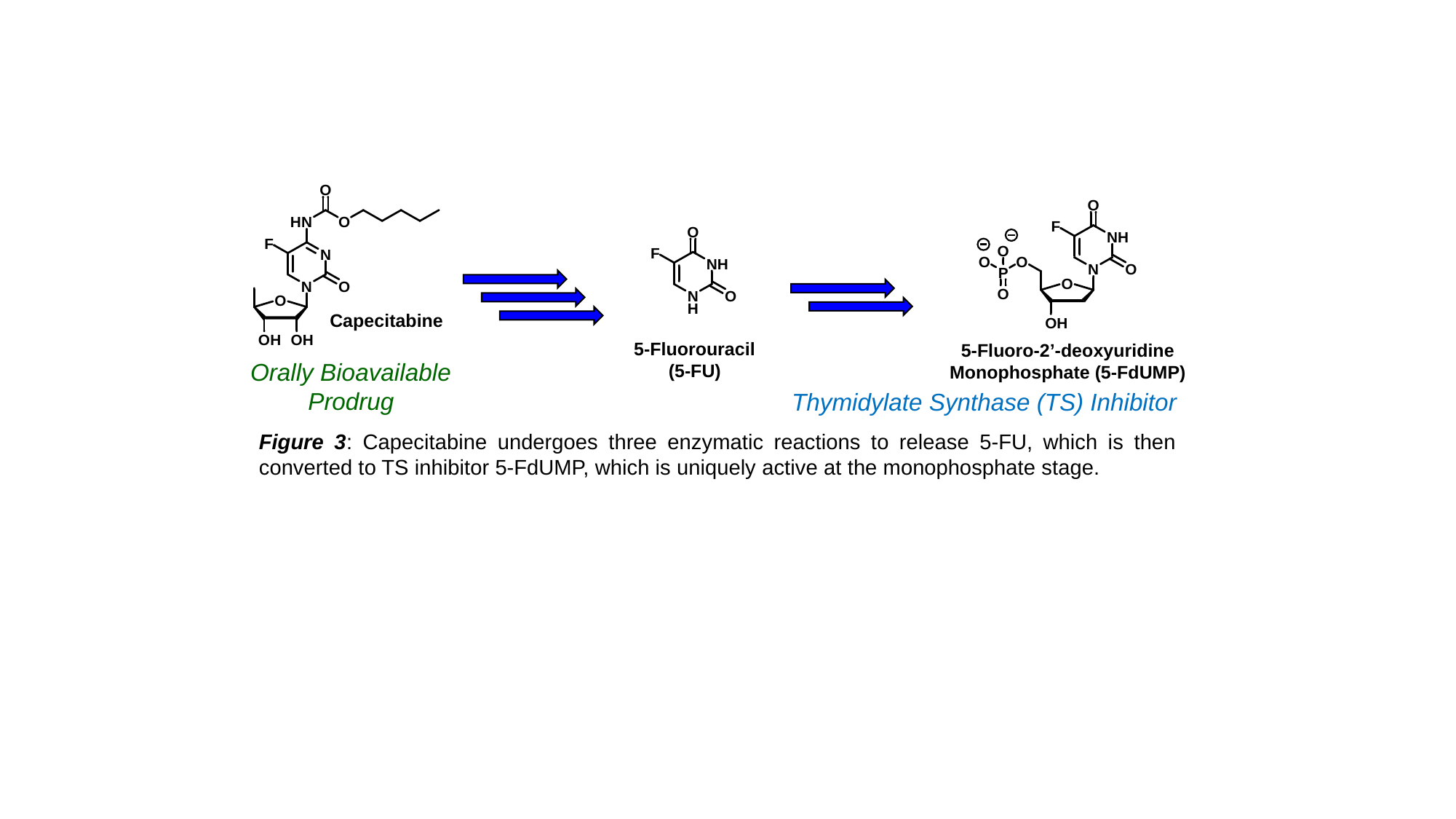

Capecitabine
5-Fluoro-2’-deoxyuridine Monophosphate (5-FdUMP)
5-Fluorouracil (5-FU)
Orally Bioavailable Prodrug
Thymidylate Synthase (TS) Inhibitor
Figure 3: Capecitabine undergoes three enzymatic reactions to release 5-FU, which is then converted to TS inhibitor 5-FdUMP, which is uniquely active at the monophosphate stage.

## Slide 4
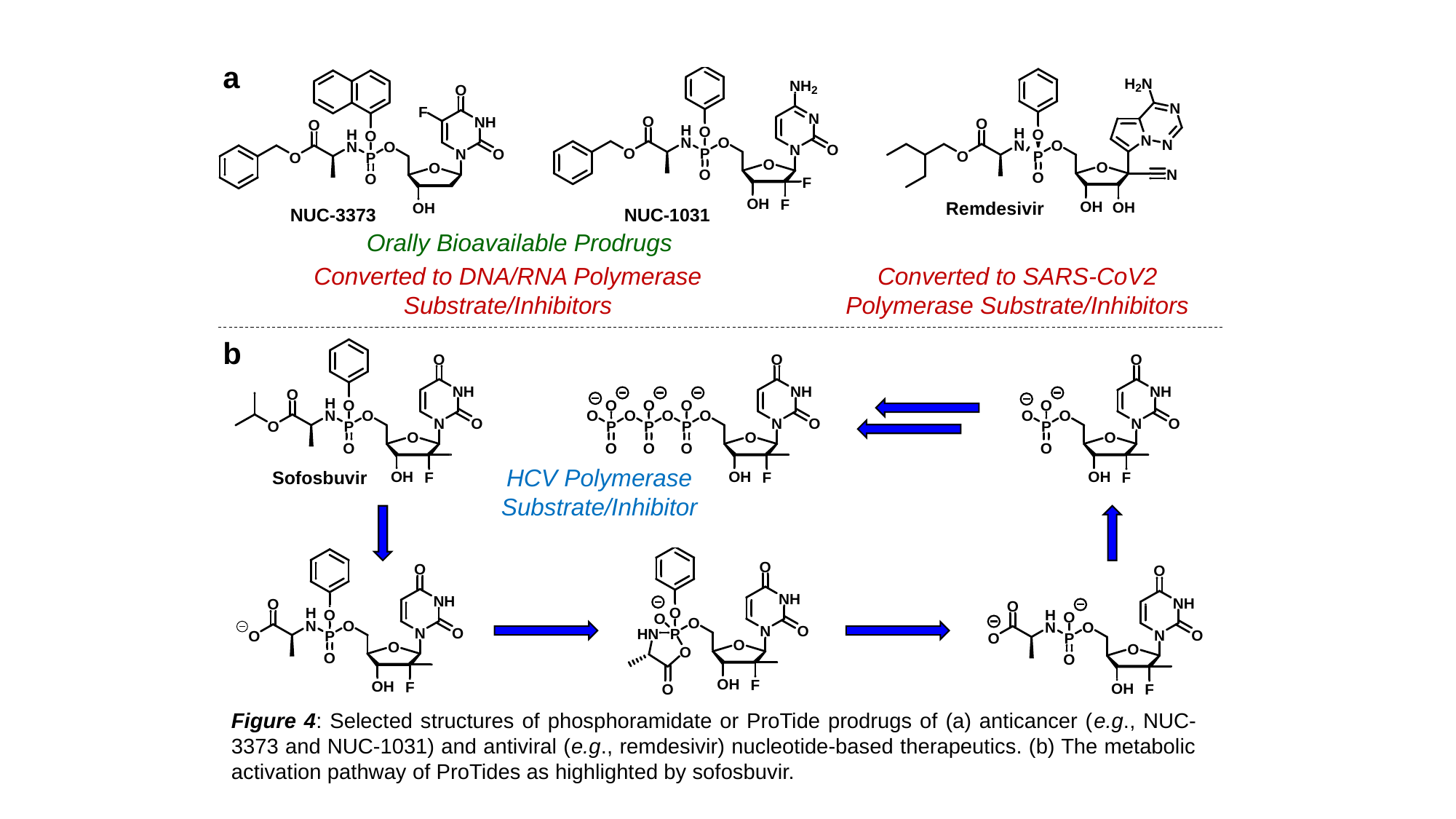

a
NUC-3373
NUC-1031
Remdesivir
Orally Bioavailable Prodrugs
Converted to DNA/RNA Polymerase Substrate/Inhibitors
Converted to SARS-CoV2 Polymerase Substrate/Inhibitors
b
Sofosbuvir
HCV Polymerase Substrate/Inhibitor
Figure 4: Selected structures of phosphoramidate or ProTide prodrugs of (a) anticancer (e.g., NUC-3373 and NUC-1031) and antiviral (e.g., remdesivir) nucleotide-based therapeutics. (b) The metabolic activation pathway of ProTides as highlighted by sofosbuvir.

## Slide 5
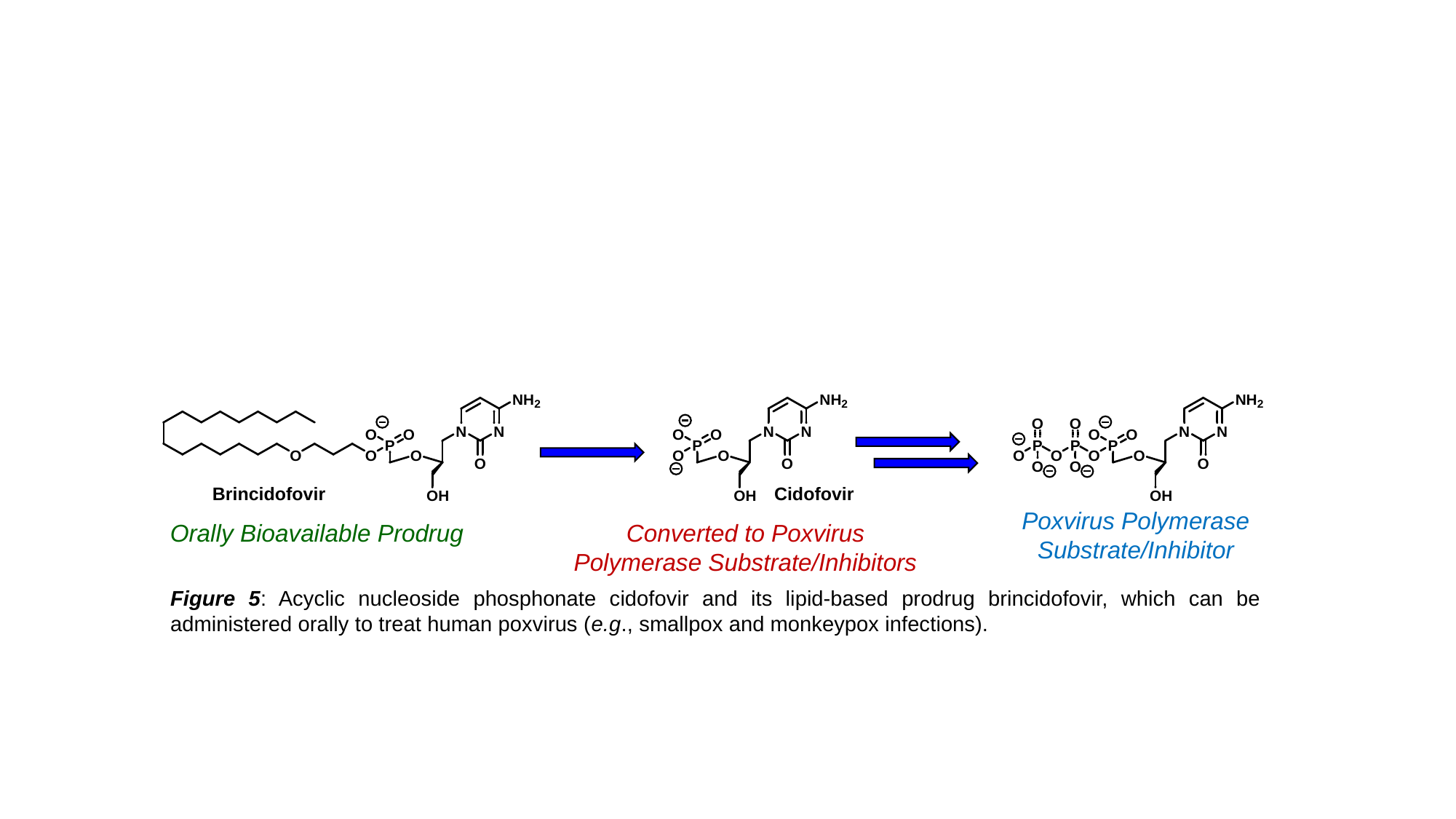

Brincidofovir
Cidofovir
Poxvirus Polymerase Substrate/Inhibitor
Orally Bioavailable Prodrug
Converted to Poxvirus Polymerase Substrate/Inhibitors
Figure 5: Acyclic nucleoside phosphonate cidofovir and its lipid-based prodrug brincidofovir, which can be administered orally to treat human poxvirus (e.g., smallpox and monkeypox infections).

## Slide 6
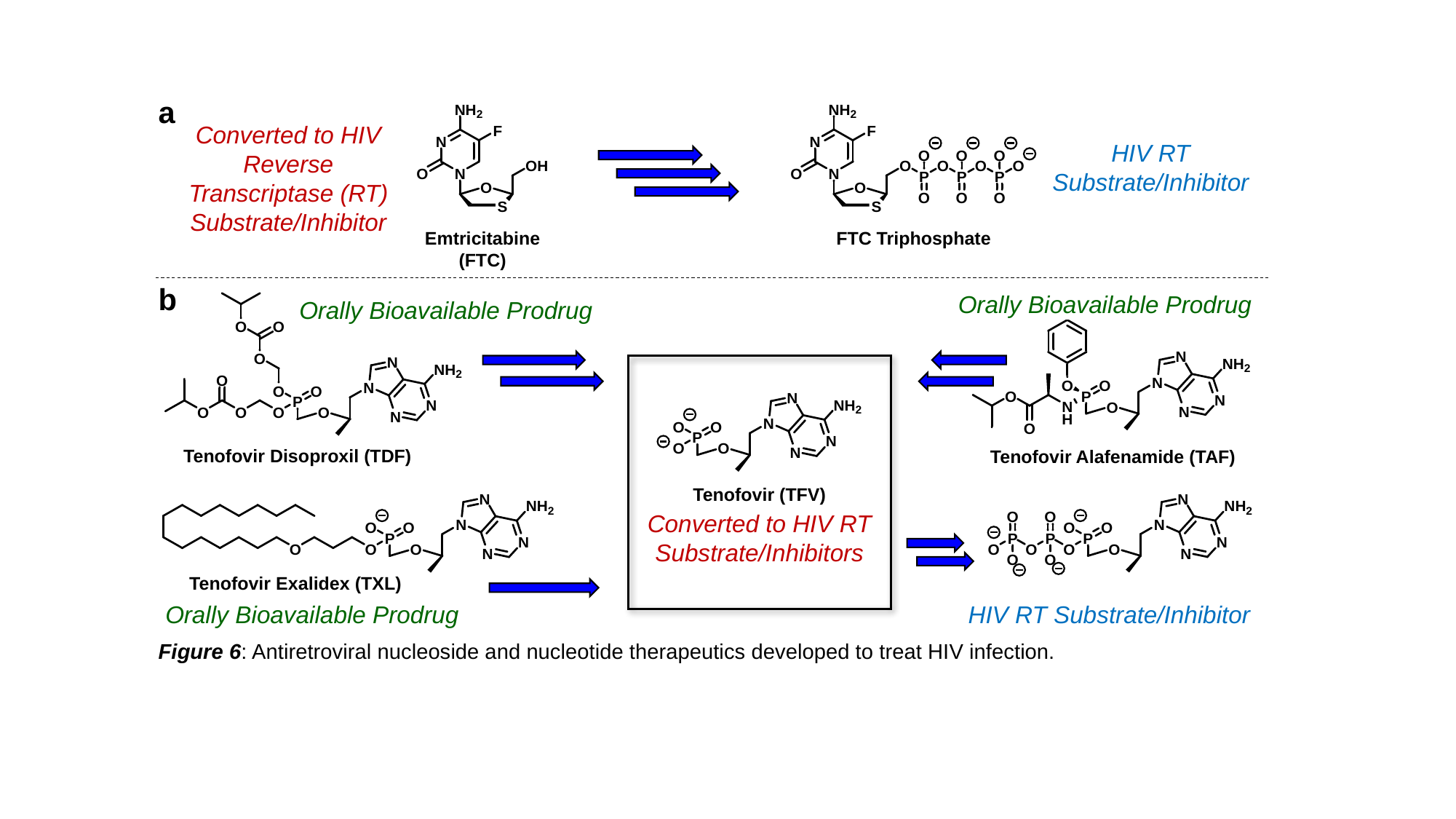

a
Emtricitabine (FTC)
FTC Triphosphate
Converted to HIV Reverse Transcriptase (RT) Substrate/Inhibitor
HIV RT Substrate/Inhibitor
b
Orally Bioavailable Prodrug
Orally Bioavailable Prodrug
Tenofovir Disoproxil (TDF)
Tenofovir Alafenamide (TAF)
Tenofovir (TFV)
Converted to HIV RT Substrate/Inhibitors
Tenofovir Exalidex (TXL)
Orally Bioavailable Prodrug
HIV RT Substrate/Inhibitor
Figure 6: Antiretroviral nucleoside and nucleotide therapeutics developed to treat HIV infection.

## Slide 7
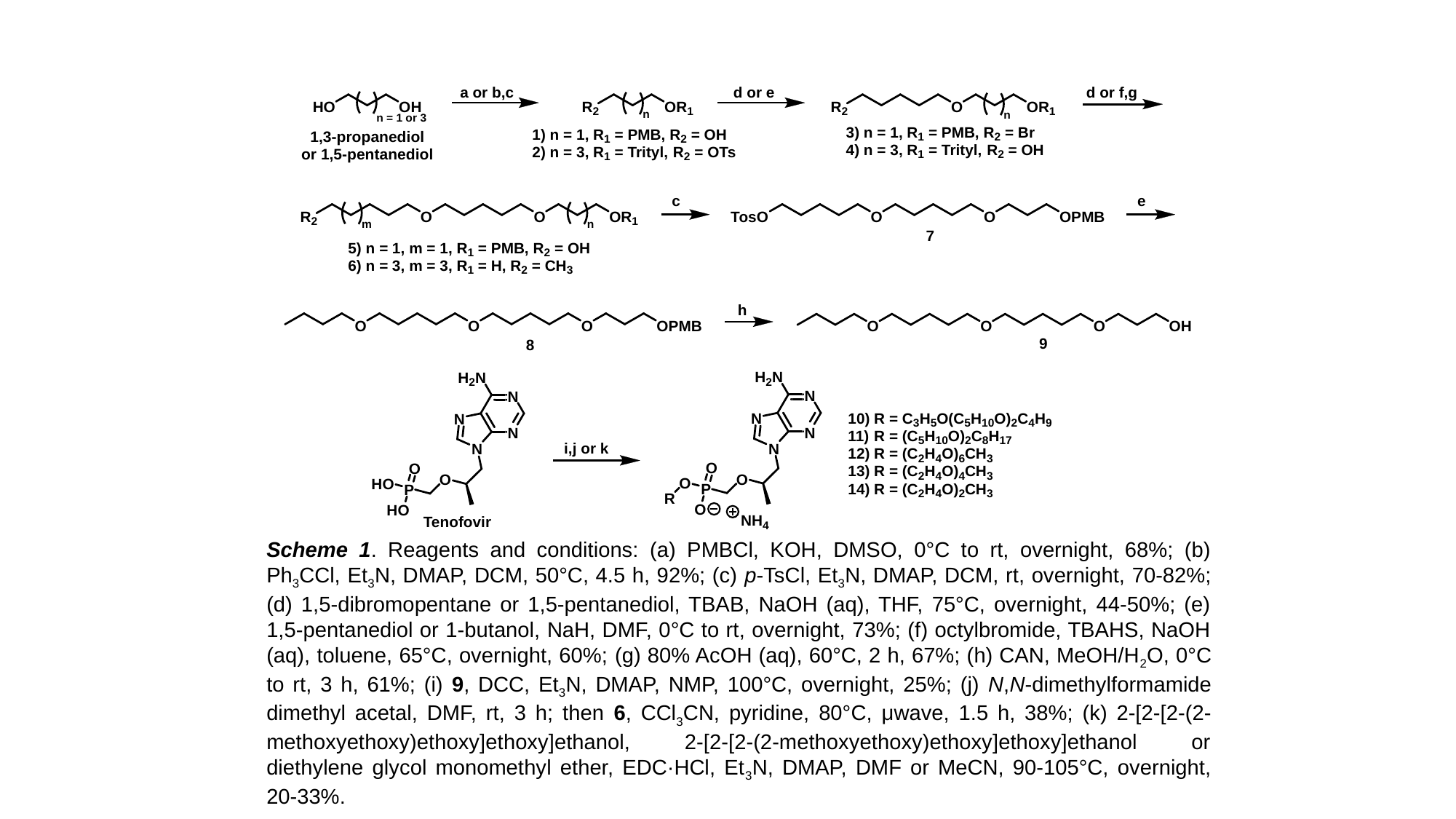

Scheme 1. Reagents and conditions: (a) PMBCl, KOH, DMSO, 0°C to rt, overnight, 68%; (b) Ph3CCl, Et3N, DMAP, DCM, 50°C, 4.5 h, 92%; (c) p-TsCl, Et3N, DMAP, DCM, rt, overnight, 70-82%; (d) 1,5-dibromopentane or 1,5-pentanediol, TBAB, NaOH (aq), THF, 75°C, overnight, 44-50%; (e) 1,5-pentanediol or 1-butanol, NaH, DMF, 0°C to rt, overnight, 73%; (f) octylbromide, TBAHS, NaOH (aq), toluene, 65°C, overnight, 60%; (g) 80% AcOH (aq), 60°C, 2 h, 67%; (h) CAN, MeOH/H2O, 0°C to rt, 3 h, 61%; (i) 9, DCC, Et3N, DMAP, NMP, 100°C, overnight, 25%; (j) N,N-dimethylformamide dimethyl acetal, DMF, rt, 3 h; then 6, CCl3CN, pyridine, 80°C, μwave, 1.5 h, 38%; (k) 2-[2-[2-(2-methoxyethoxy)ethoxy]ethoxy]ethanol, 2-[2-[2-(2-methoxyethoxy)ethoxy]ethoxy]ethanol or diethylene glycol monomethyl ether, EDC·HCl, Et3N, DMAP, DMF or MeCN, 90-105°C, overnight, 20-33%.

## Slide 8
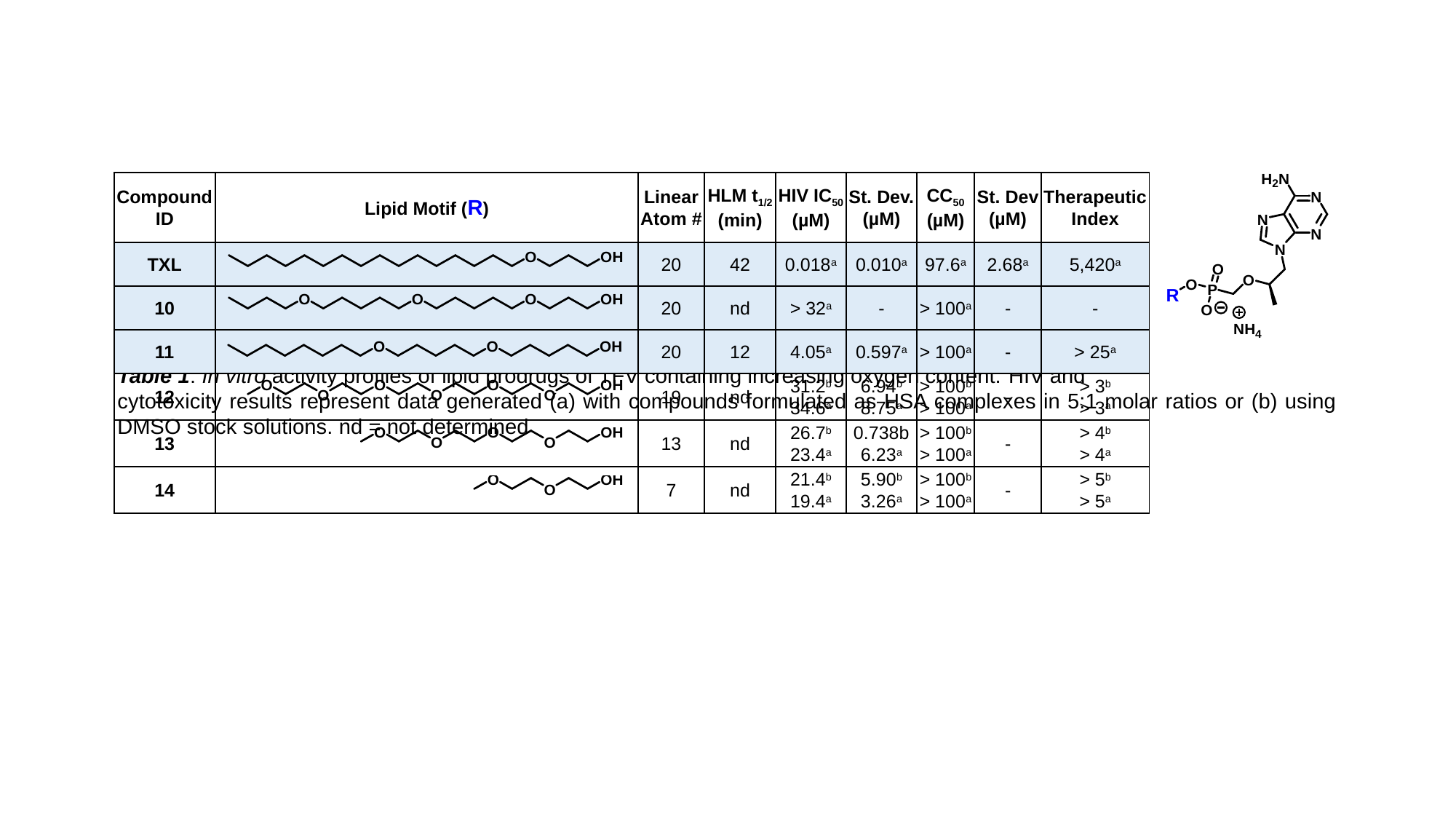

| Compound ID | Lipid Motif (R) | Linear Atom # | HLM t1/2 (min) | HIV IC50 (µM) | St. Dev. (µM) | CC50 (µM) | St. Dev (µM) | Therapeutic Index |
| --- | --- | --- | --- | --- | --- | --- | --- | --- |
| TXL | | 20 | 42 | 0.018a | 0.010a | 97.6a | 2.68a | 5,420a |
| 10 | | 20 | nd | > 32a | - | > 100a | - | - |
| 11 | | 20 | 12 | 4.05a | 0.597a | > 100a | - | > 25a |
| 12 | | 19 | nd | 31.2b 34.6a | 6.94b 8.75a | > 100b > 100a | - | > 3b > 3a |
| 13 | | 13 | nd | 26.7b 23.4a | 0.738b 6.23a | > 100b > 100a | - | > 4b > 4a |
| 14 | | 7 | nd | 21.4b 19.4a | 5.90b 3.26a | > 100b > 100a | - | > 5b > 5a |
Table 1: In vitro activity profiles of lipid prodrugs of TFV containing increasing oxygen content. HIV and
cytotoxicity results represent data generated (a) with compounds formulated as HSA complexes in 5:1 molar ratios or (b) using DMSO stock solutions. nd = not determined

## Slide 9
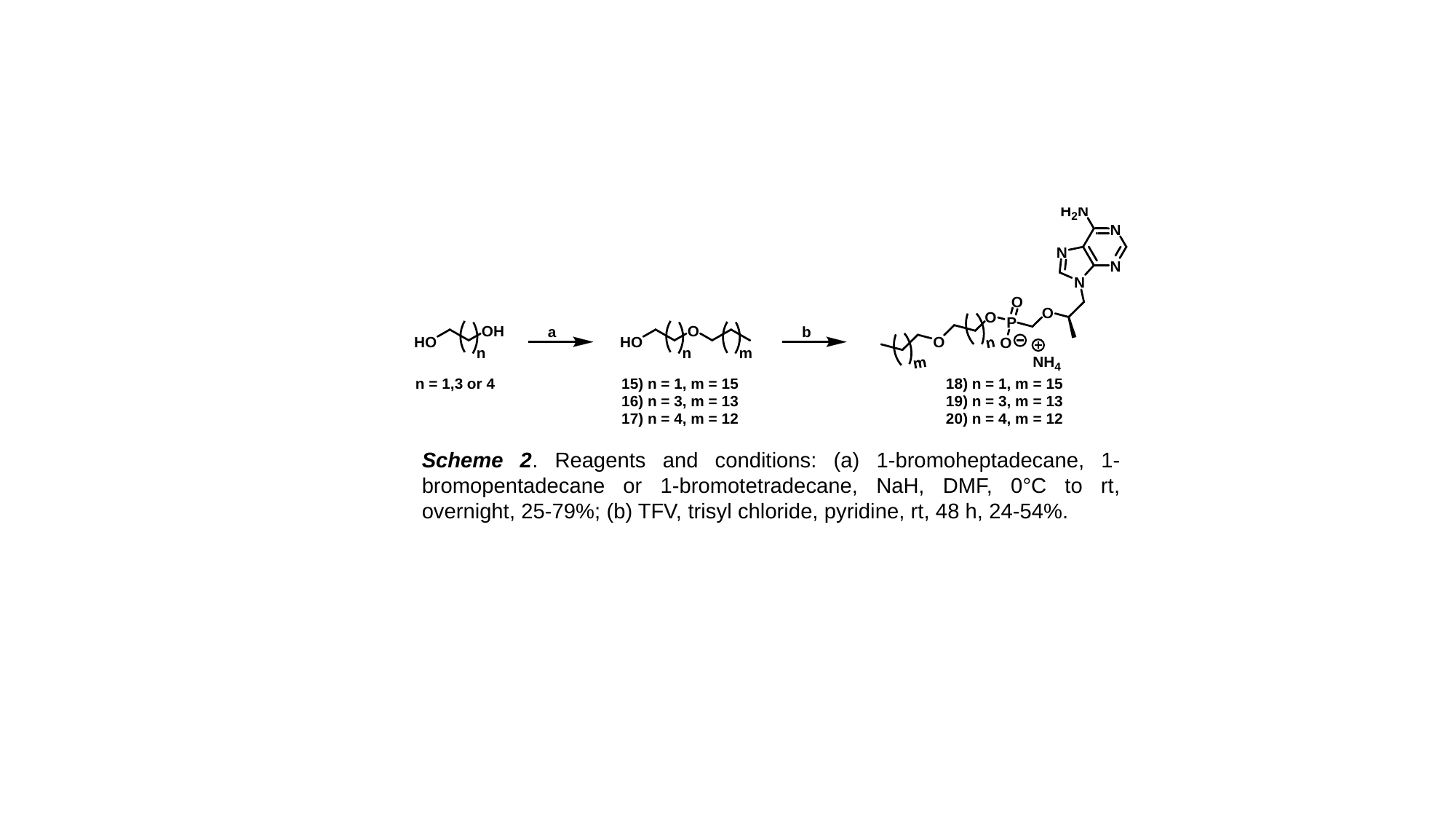

Scheme 2. Reagents and conditions: (a) 1-bromoheptadecane, 1-bromopentadecane or 1-bromotetradecane, NaH, DMF, 0°C to rt, overnight, 25-79%; (b) TFV, trisyl chloride, pyridine, rt, 48 h, 24-54%.

## Slide 10
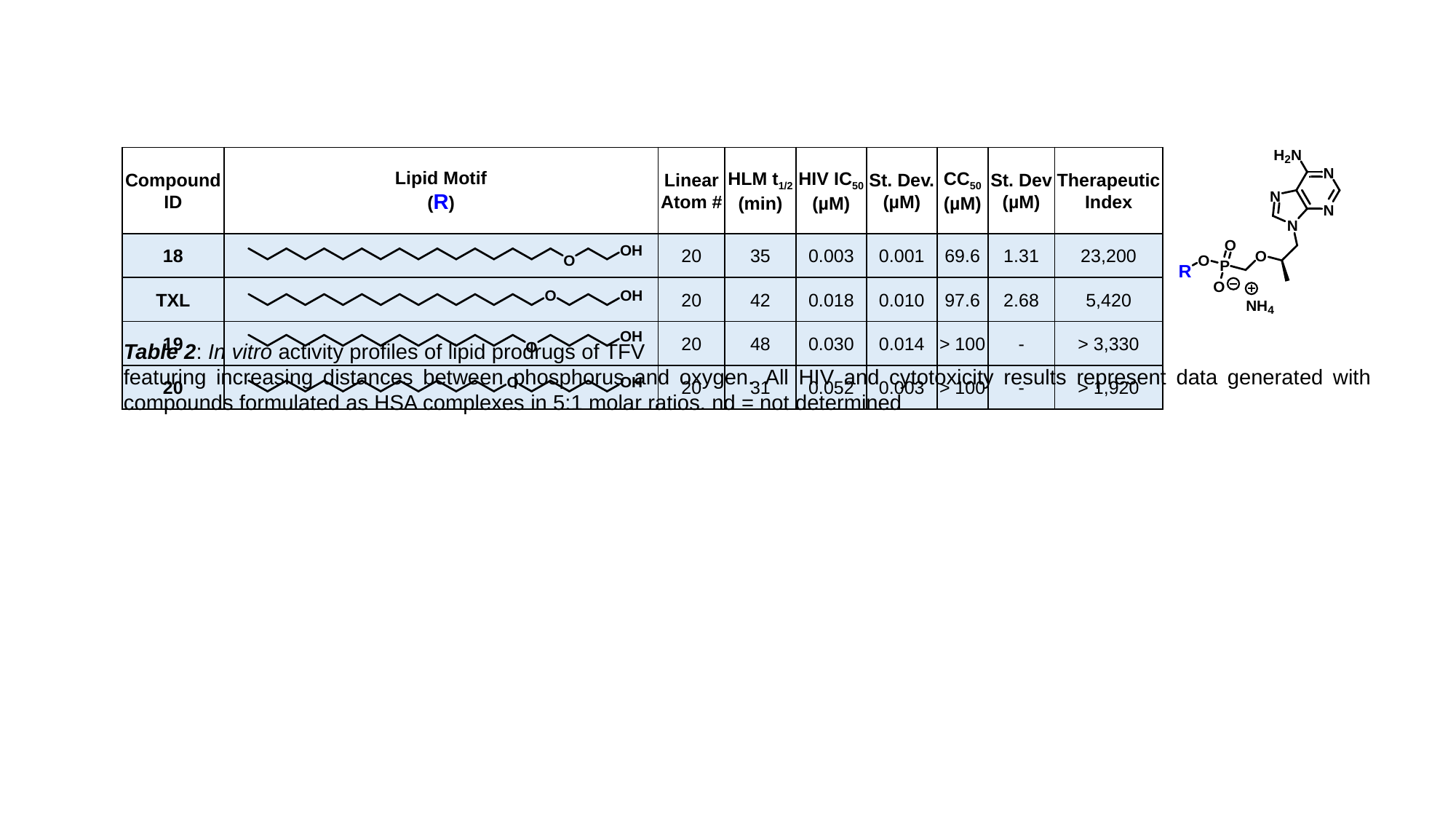

| Compound ID | Lipid Motif (R) | Linear Atom # | HLM t1/2 (min) | HIV IC50 (µM) | St. Dev. (µM) | CC50 (µM) | St. Dev (µM) | Therapeutic Index |
| --- | --- | --- | --- | --- | --- | --- | --- | --- |
| 18 | | 20 | 35 | 0.003 | 0.001 | 69.6 | 1.31 | 23,200 |
| TXL | | 20 | 42 | 0.018 | 0.010 | 97.6 | 2.68 | 5,420 |
| 19 | | 20 | 48 | 0.030 | 0.014 | > 100 | - | > 3,330 |
| 20 | | 20 | 31 | 0.052 | 0.003 | > 100 | - | > 1,920 |
Table 2: In vitro activity profiles of lipid prodrugs of TFV
featuring increasing distances between phosphorus and oxygen. All HIV and cytotoxicity results represent data generated with compounds formulated as HSA complexes in 5:1 molar ratios. nd = not determined

## Slide 11
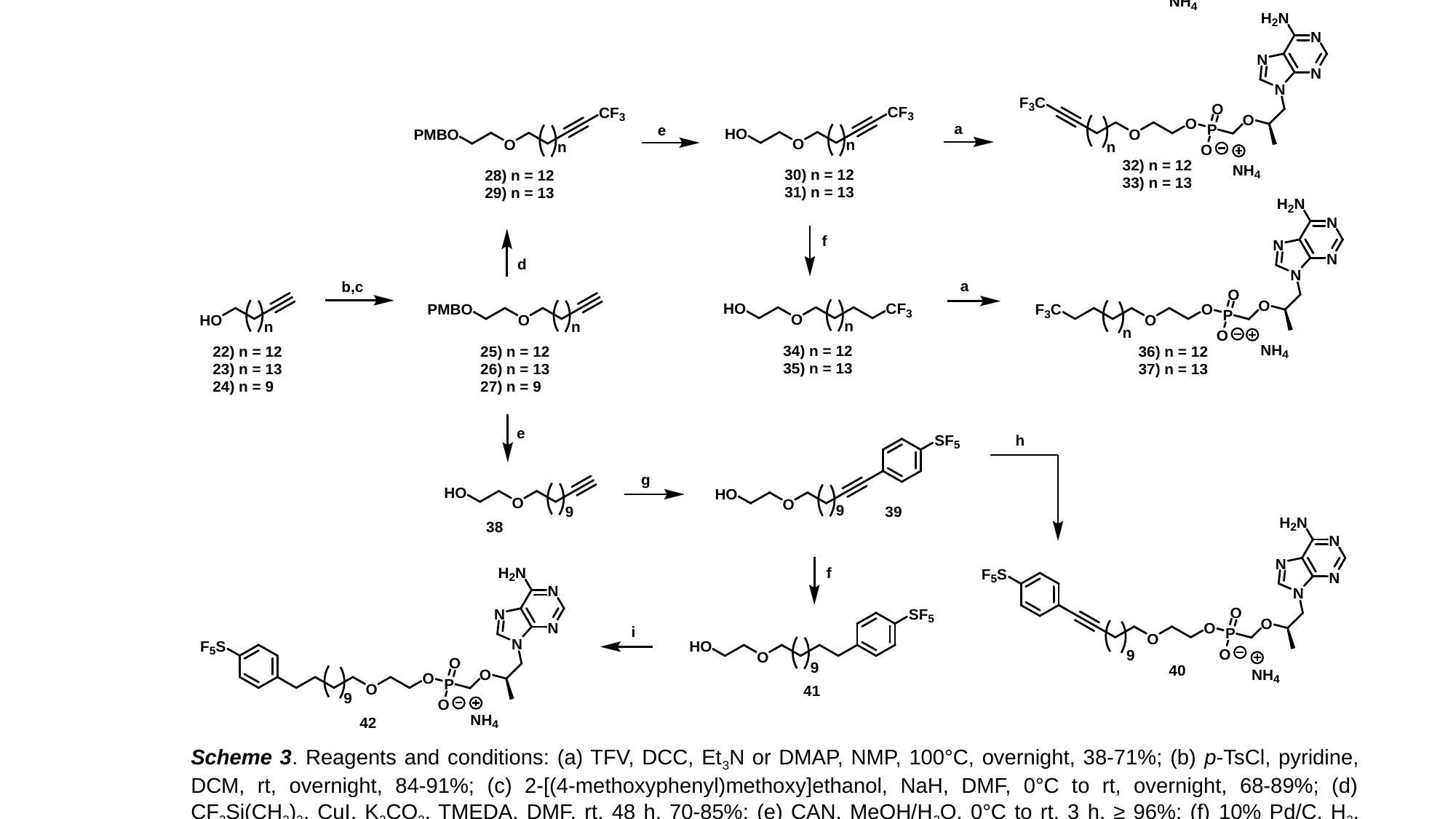

Scheme 3. Reagents and conditions: (a) TFV, DCC, Et3N or DMAP, NMP, 100°C, overnight, 38-71%; (b) p-TsCl, pyridine, DCM, rt, overnight, 84-91%; (c) 2-[(4-methoxyphenyl)methoxy]ethanol, NaH, DMF, 0°C to rt, overnight, 68-89%; (d) CF3Si(CH3)3, CuI, K2CO3, TMEDA, DMF, rt, 48 h, 70-85%; (e) CAN, MeOH/H2O, 0°C to rt, 3 h, ≥ 96%; (f) 10% Pd/C, H2, EtOAc, rt, 2-3 h, 82-88%; (g) 4-iodophenylsulfurpentafluoride, Pd(PPh3)2Cl2, CuI, Et3N, THF, 55°C, 2 h, 68%; (h) TFV, TCFH, 1-methylimidazole, Et3N, NMP, rt, overnight, 36%; (i) TFV, trisyl chloride, 1-methylimidazole, DMF, rt to 100°C, 48 h, 24%.

## Slide 12
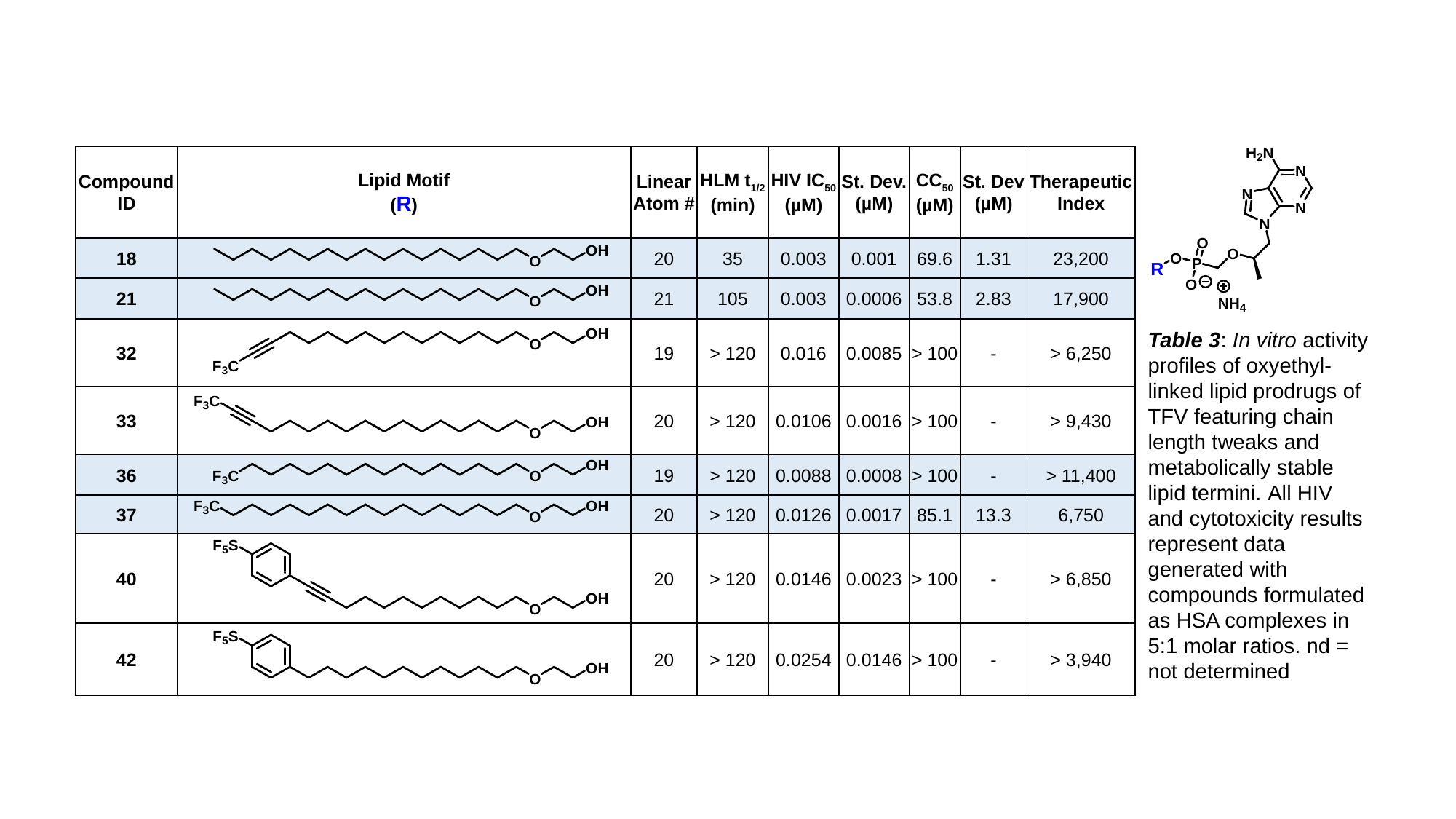

| Compound ID | Lipid Motif (R) | Linear Atom # | HLM t1/2 (min) | HIV IC50 (µM) | St. Dev. (µM) | CC50 (µM) | St. Dev (µM) | Therapeutic Index |
| --- | --- | --- | --- | --- | --- | --- | --- | --- |
| 18 | | 20 | 35 | 0.003 | 0.001 | 69.6 | 1.31 | 23,200 |
| 21 | | 21 | 105 | 0.003 | 0.0006 | 53.8 | 2.83 | 17,900 |
| 32 | | 19 | > 120 | 0.016 | 0.0085 | > 100 | - | > 6,250 |
| 33 | | 20 | > 120 | 0.0106 | 0.0016 | > 100 | - | > 9,430 |
| 36 | | 19 | > 120 | 0.0088 | 0.0008 | > 100 | - | > 11,400 |
| 37 | | 20 | > 120 | 0.0126 | 0.0017 | 85.1 | 13.3 | 6,750 |
| 40 | | 20 | > 120 | 0.0146 | 0.0023 | > 100 | - | > 6,850 |
| 42 | | 20 | > 120 | 0.0254 | 0.0146 | > 100 | - | > 3,940 |
Table 3: In vitro activity profiles of oxyethyl-linked lipid prodrugs of TFV featuring chain length tweaks and metabolically stable lipid termini. All HIV and cytotoxicity results represent data generated with compounds formulated as HSA complexes in 5:1 molar ratios. nd = not determined

## Slide 13
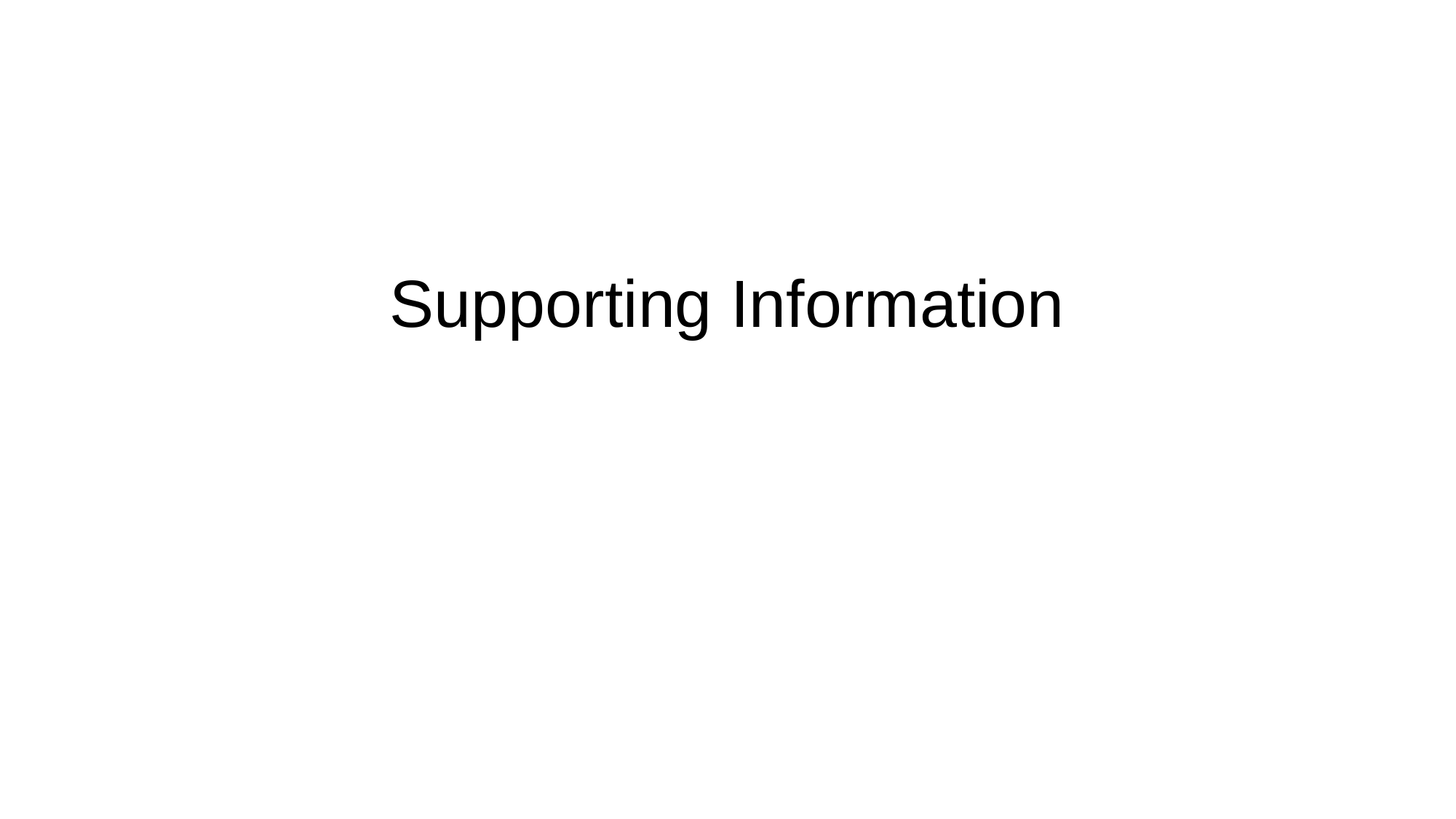

Supporting Information

## Slide 14
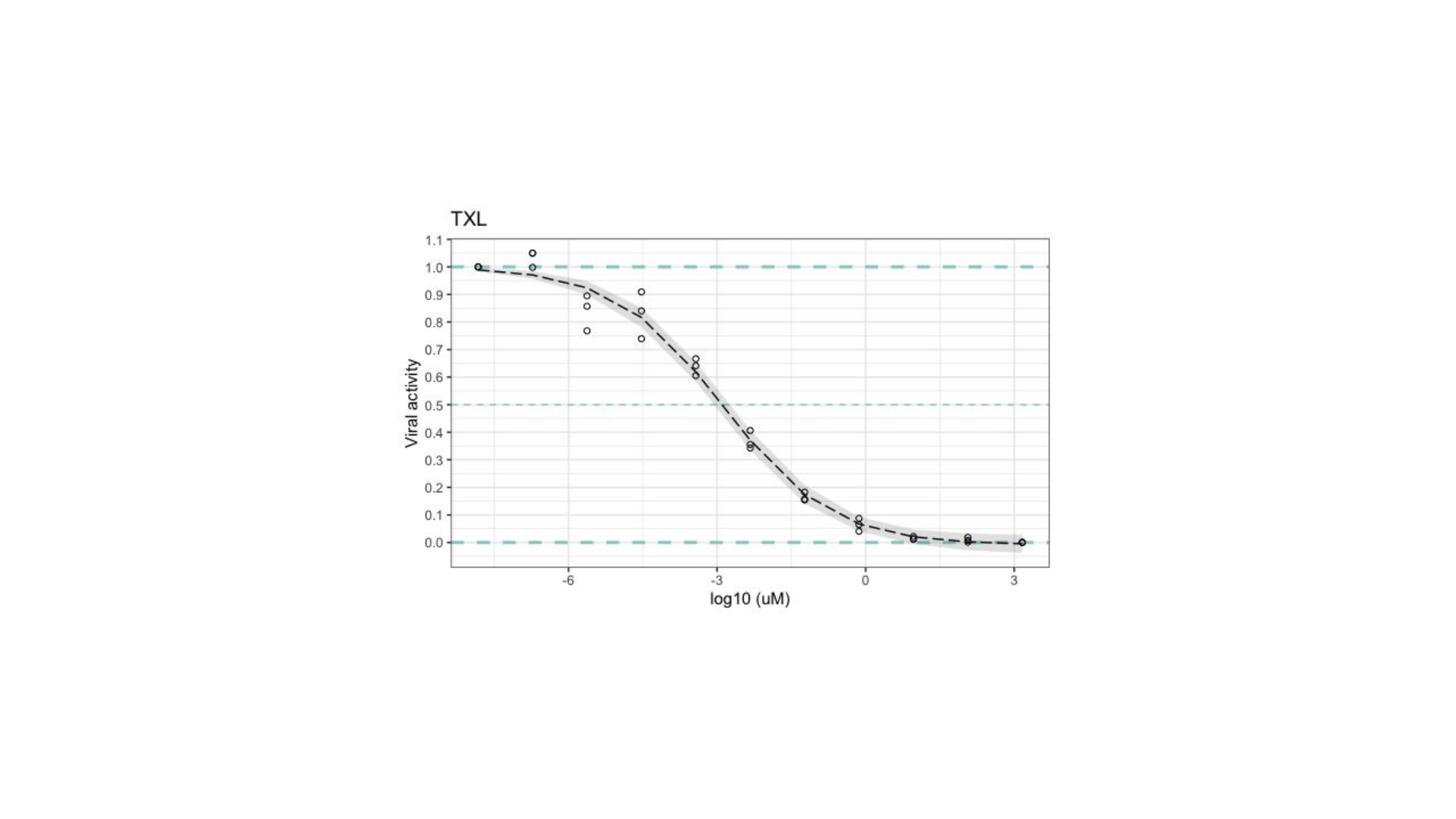

## Slide 15
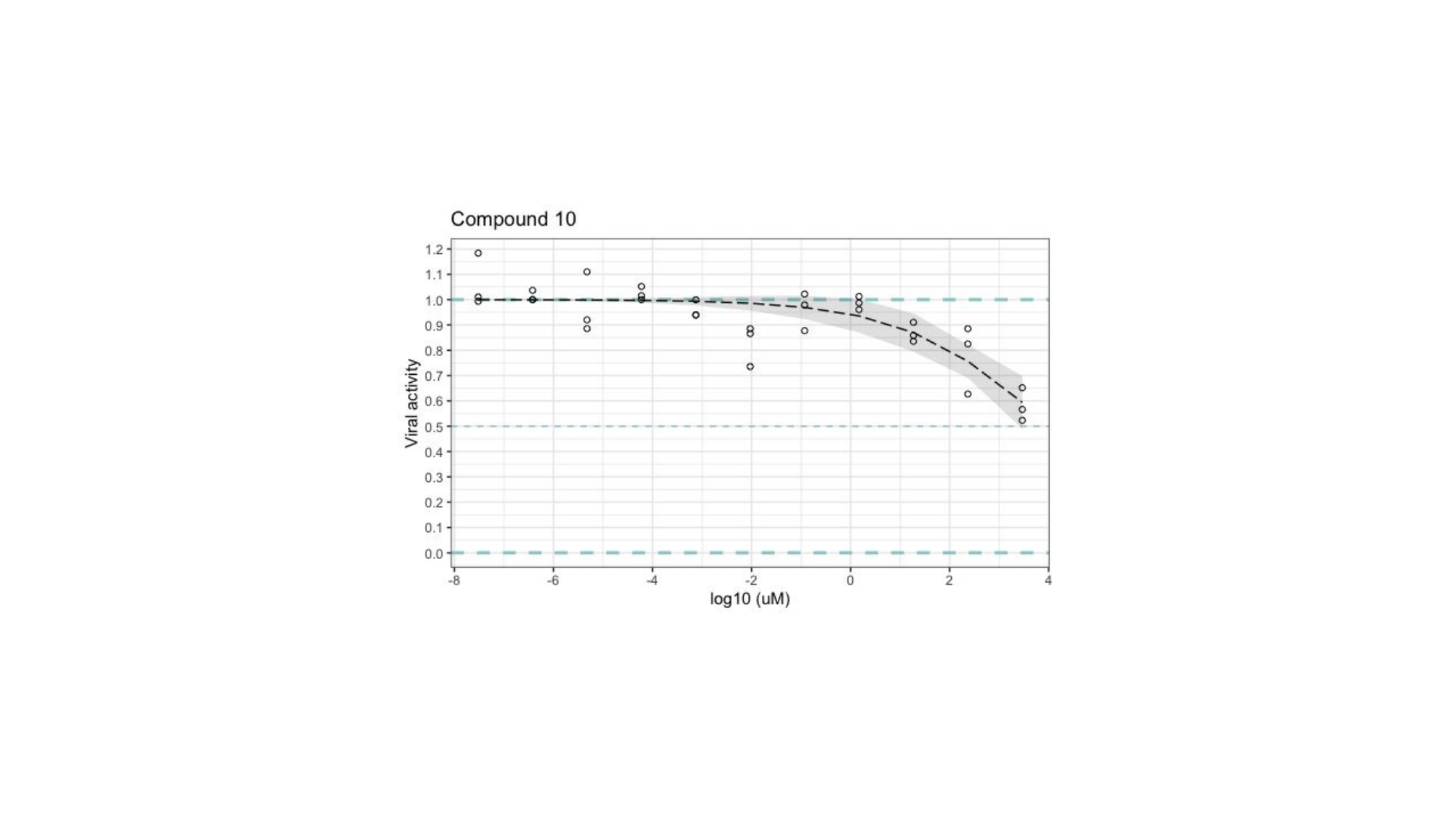

## Slide 16
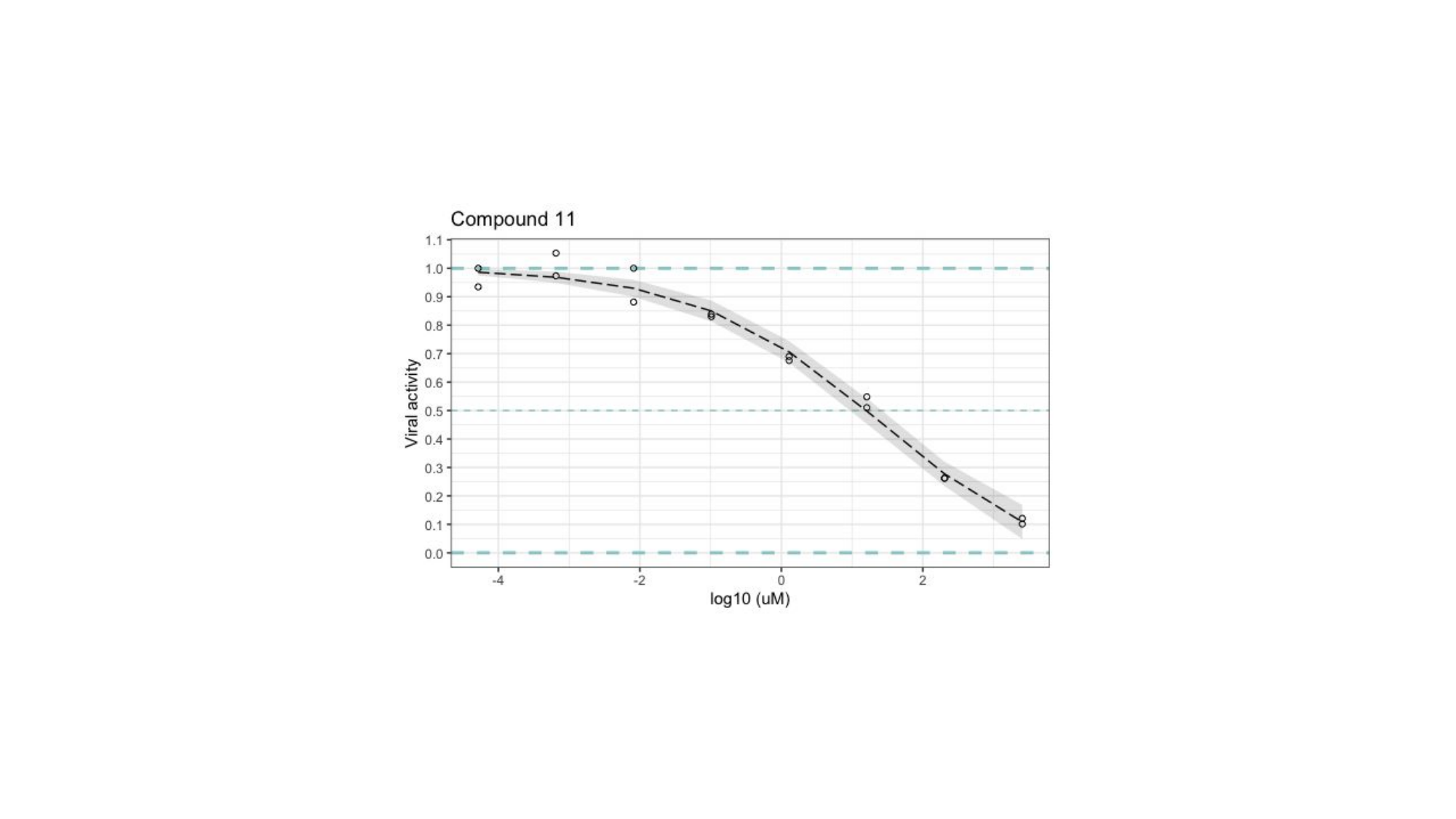

## Slide 17
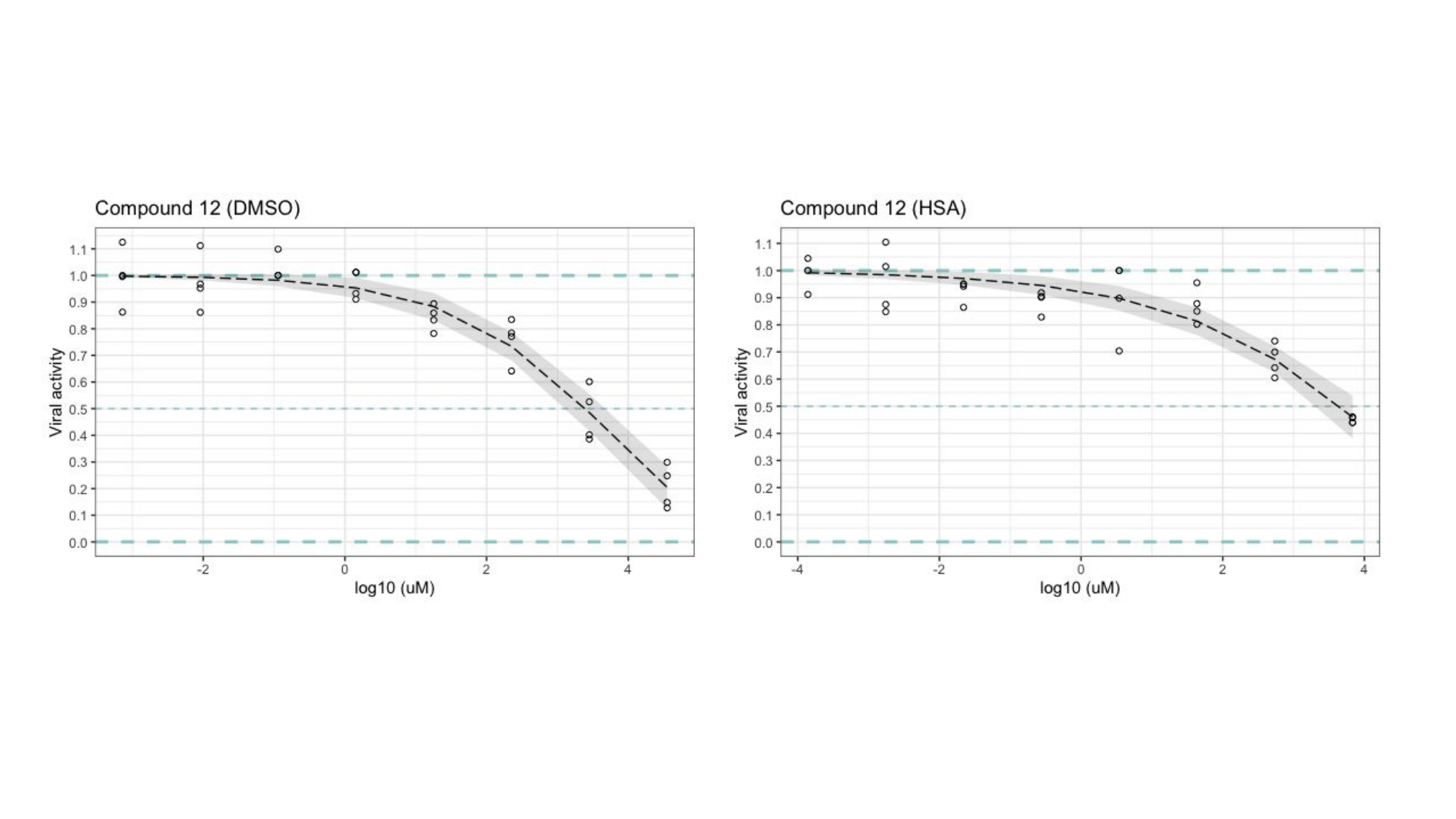

## Slide 18
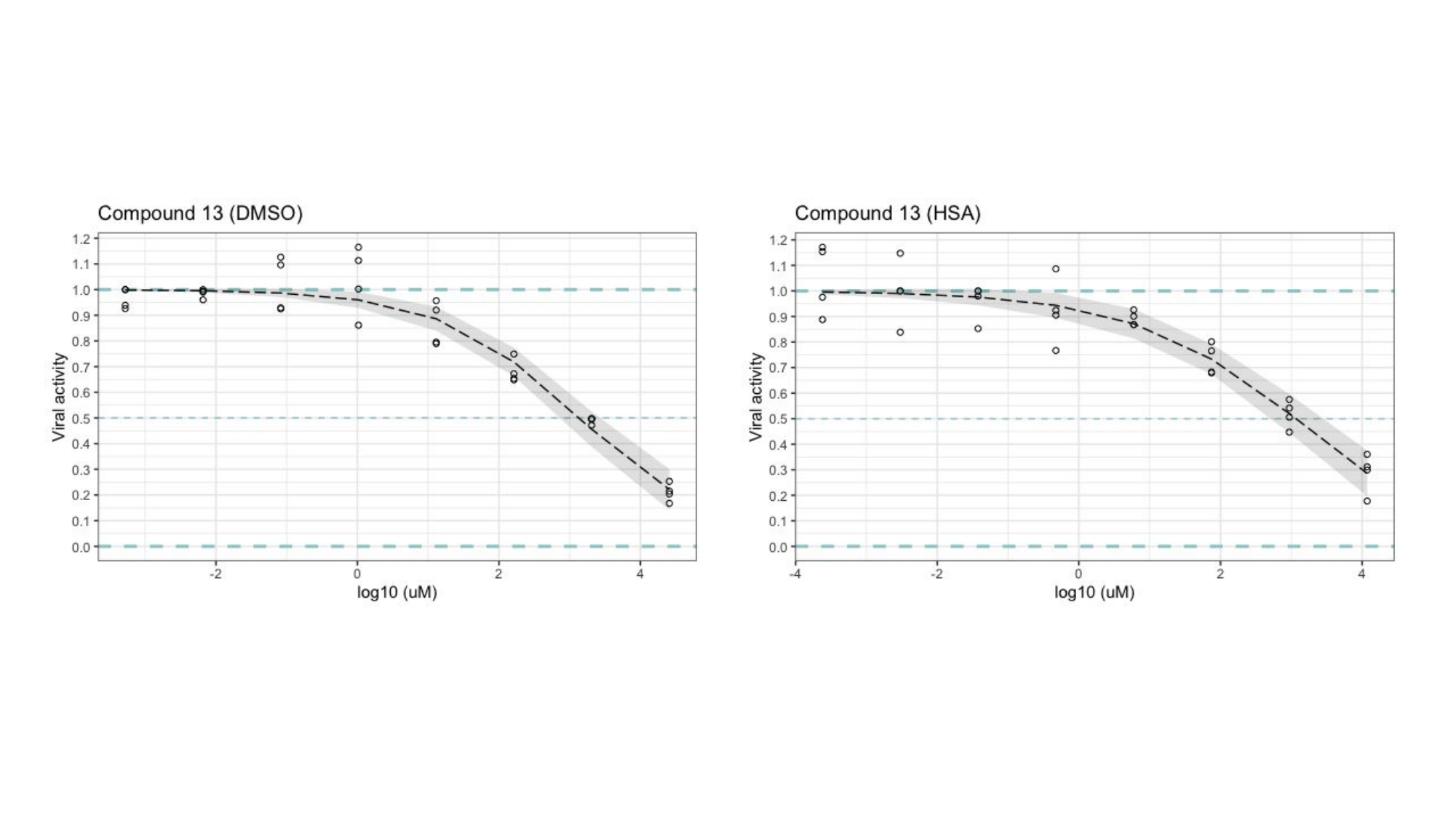

## Slide 19
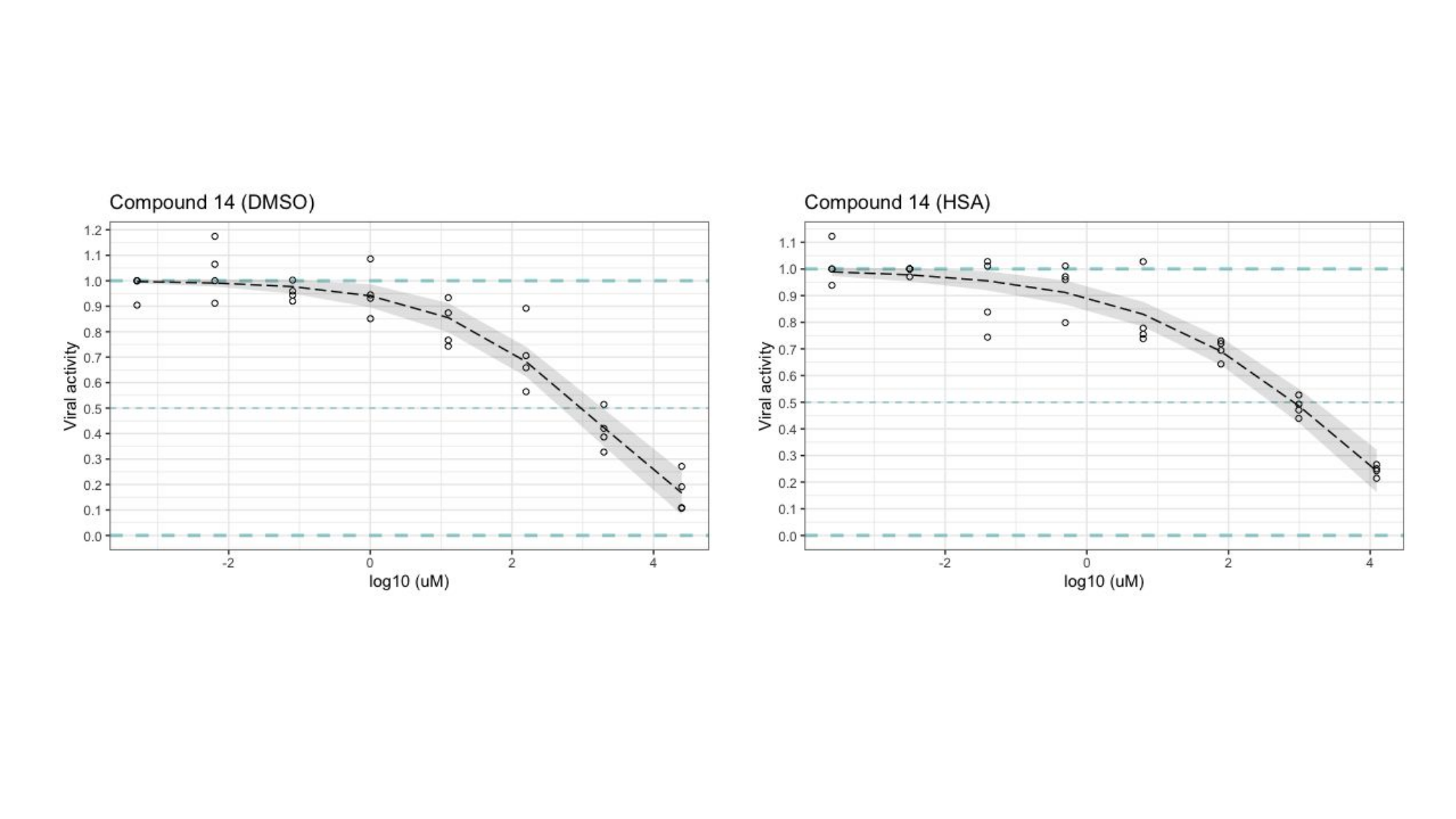

## Slide 20
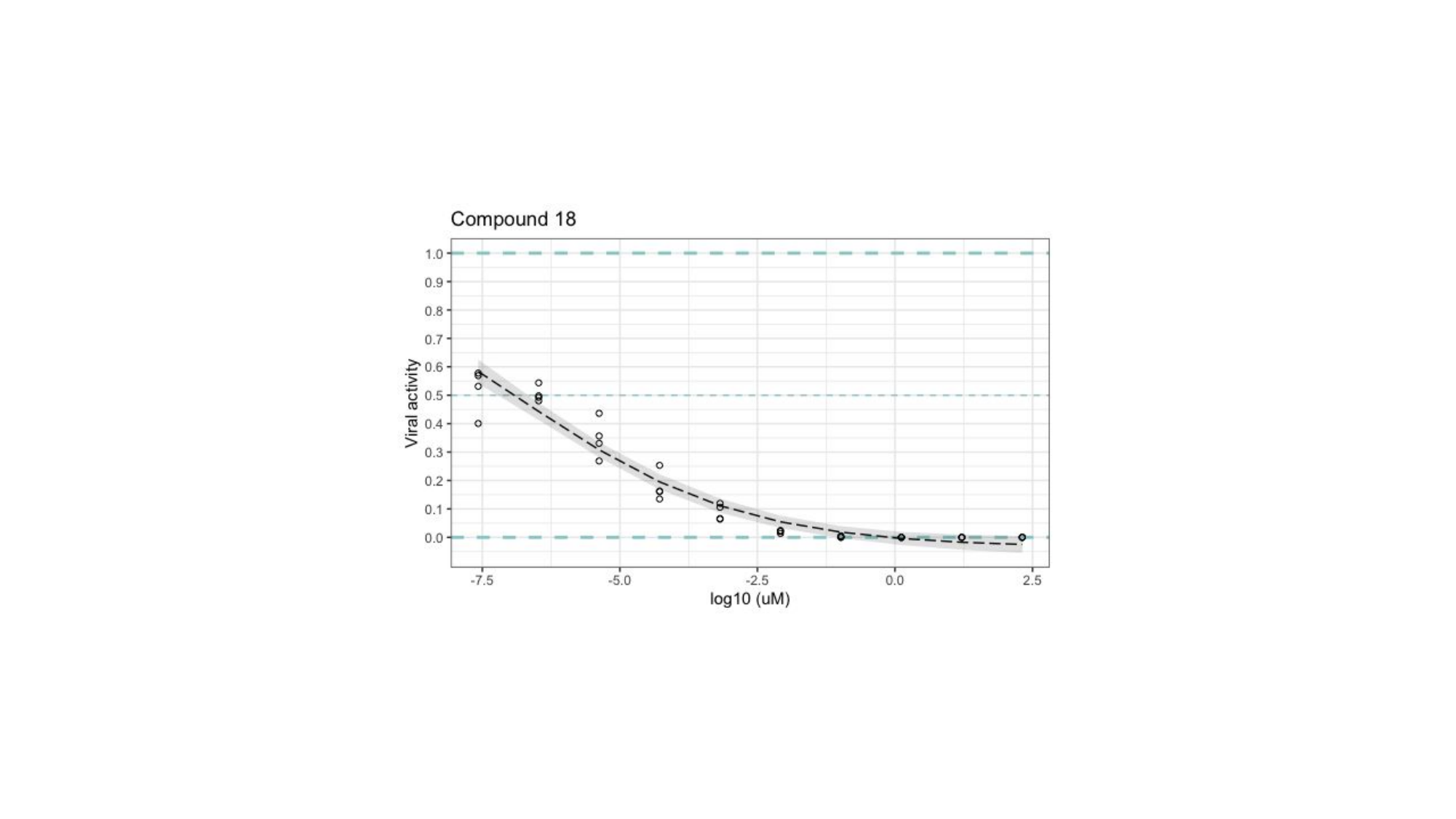

## Slide 21
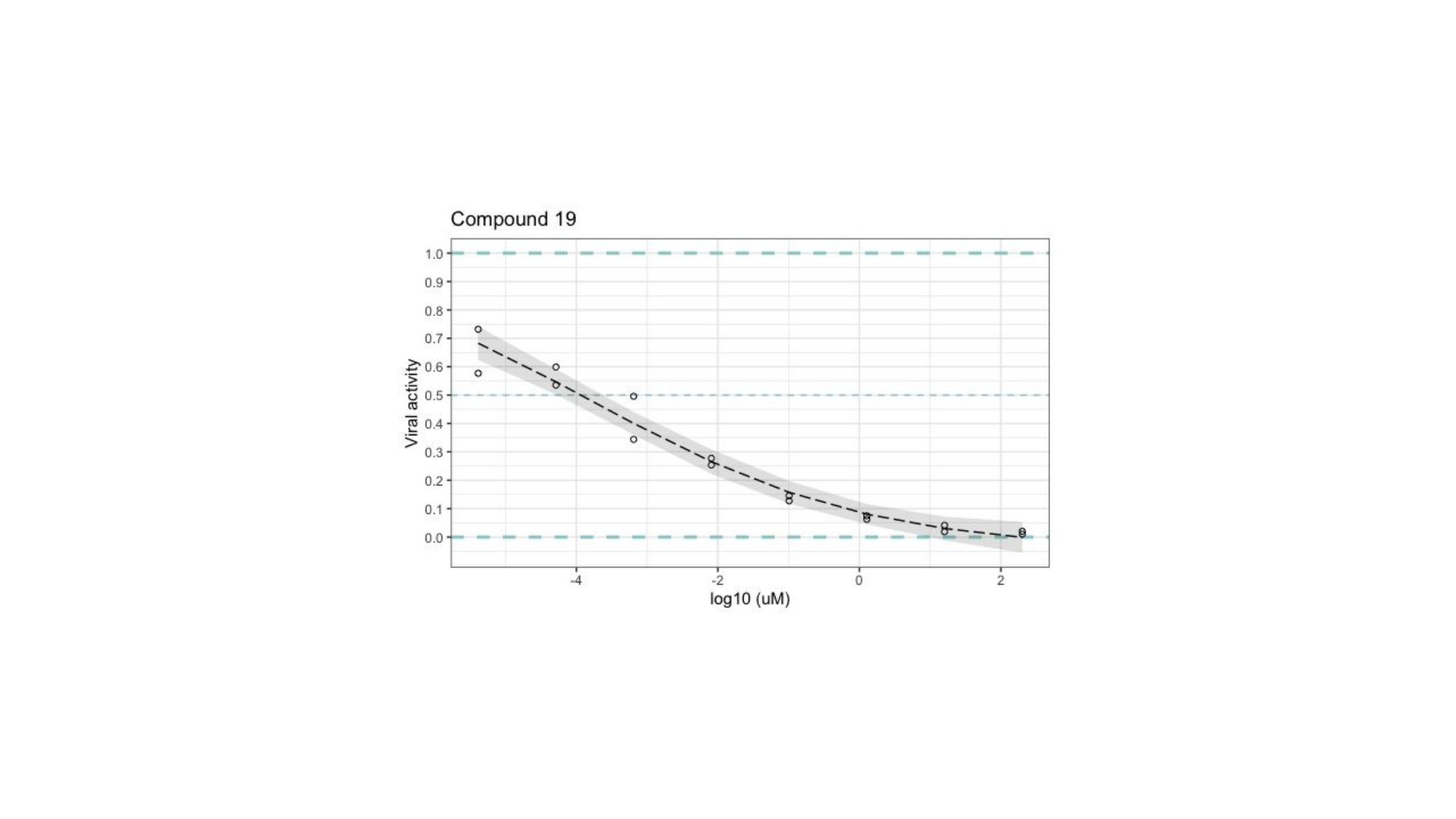

## Slide 22
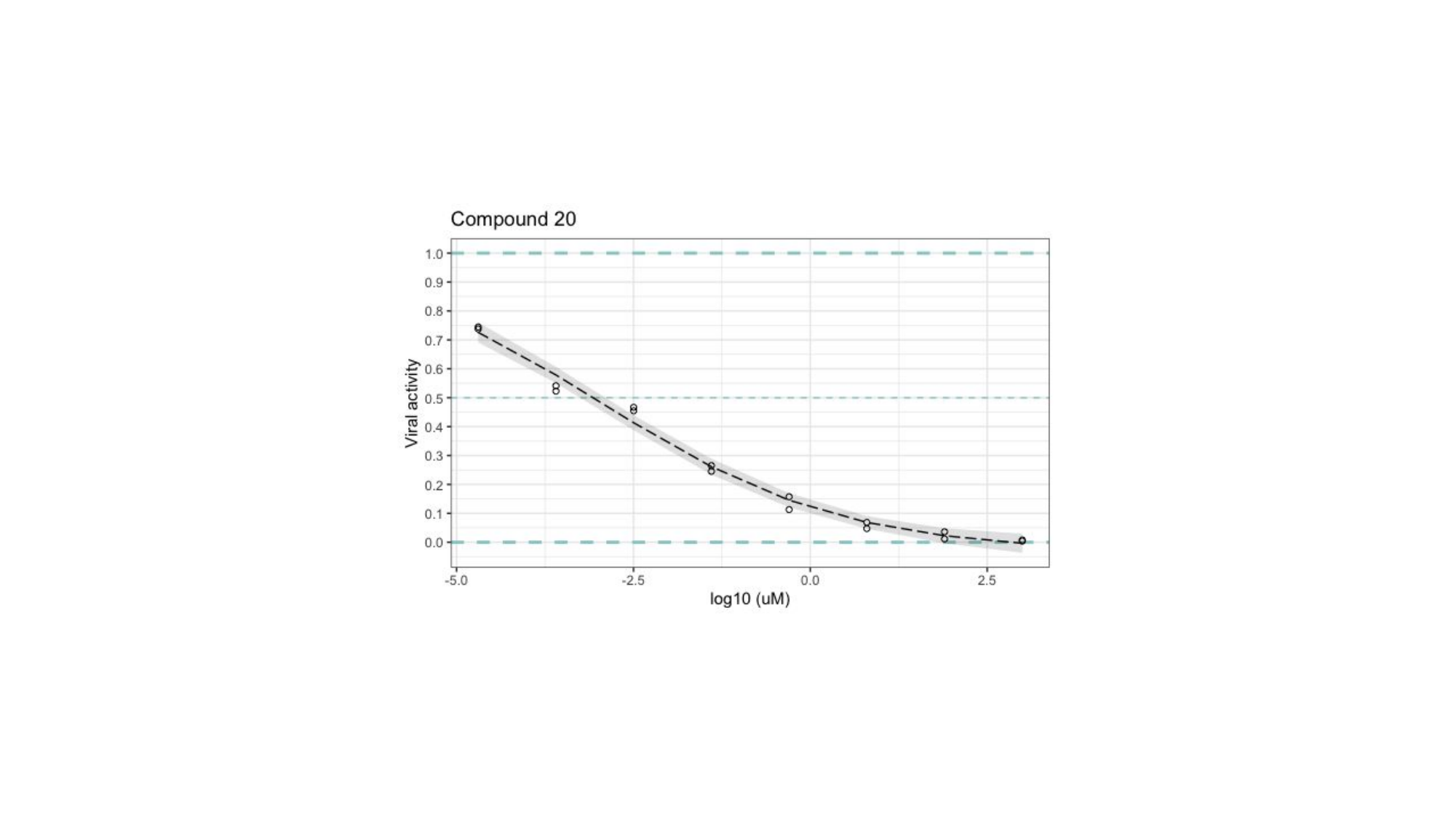

## Slide 23
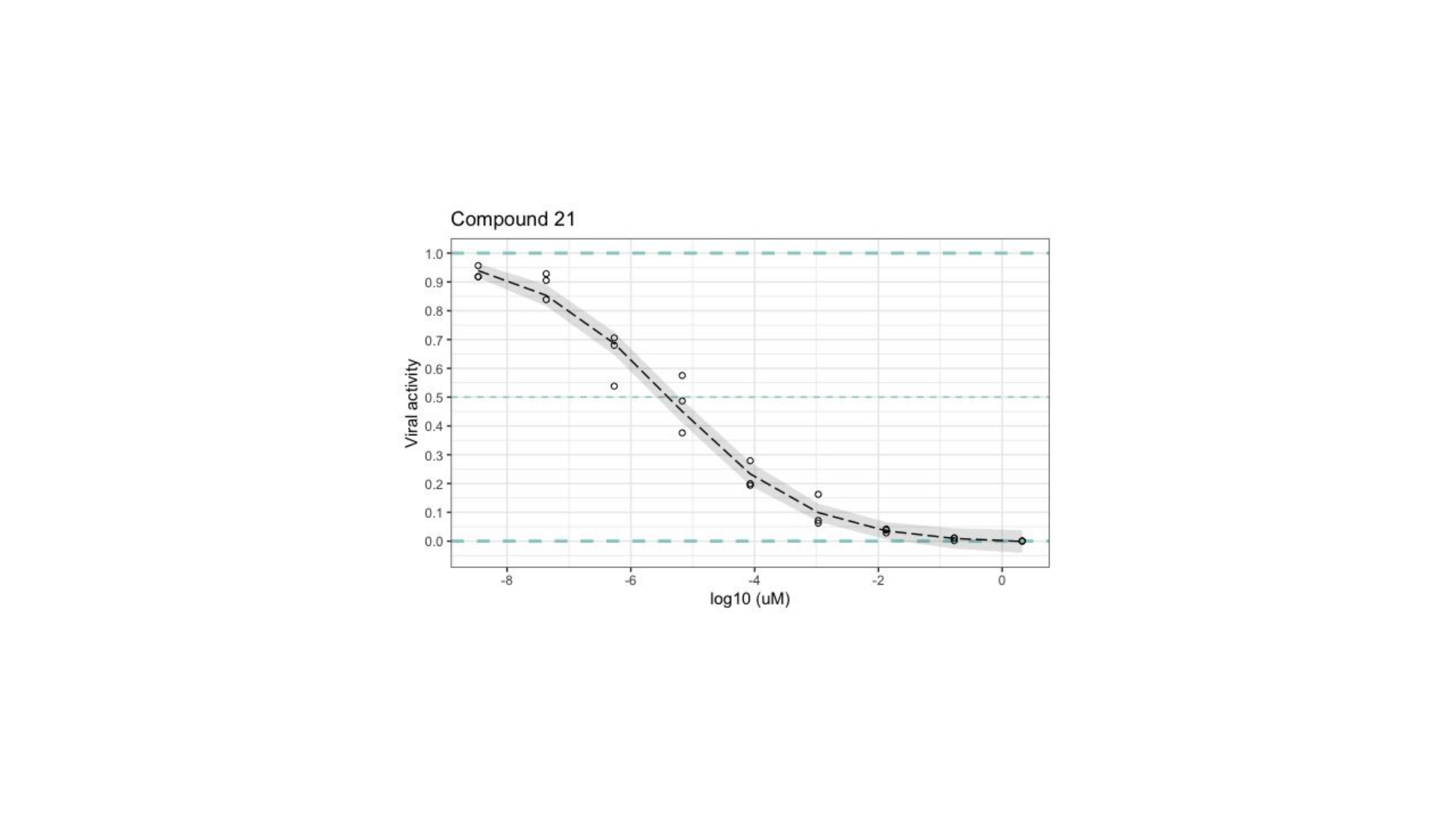

## Slide 24
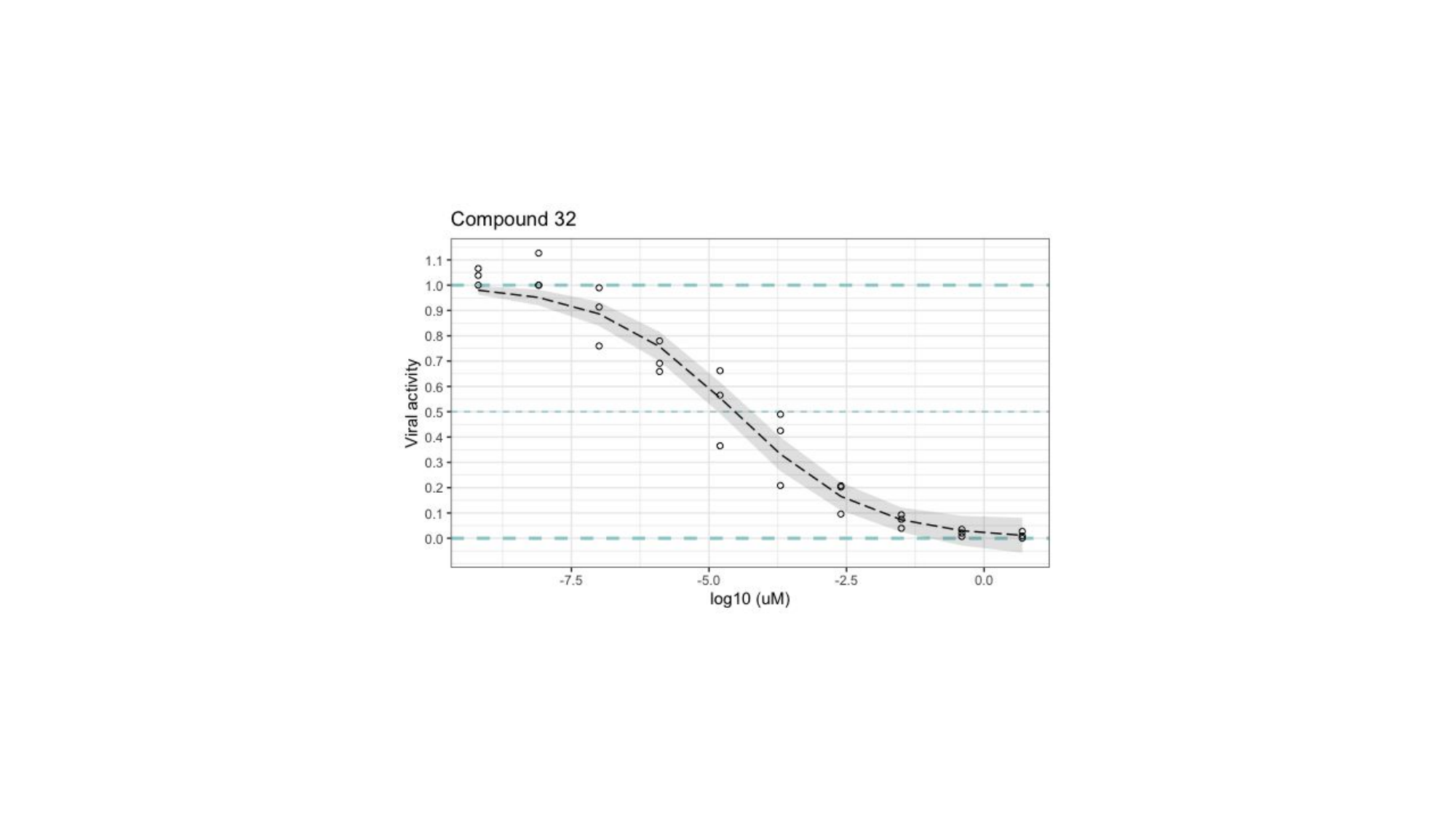

## Slide 25
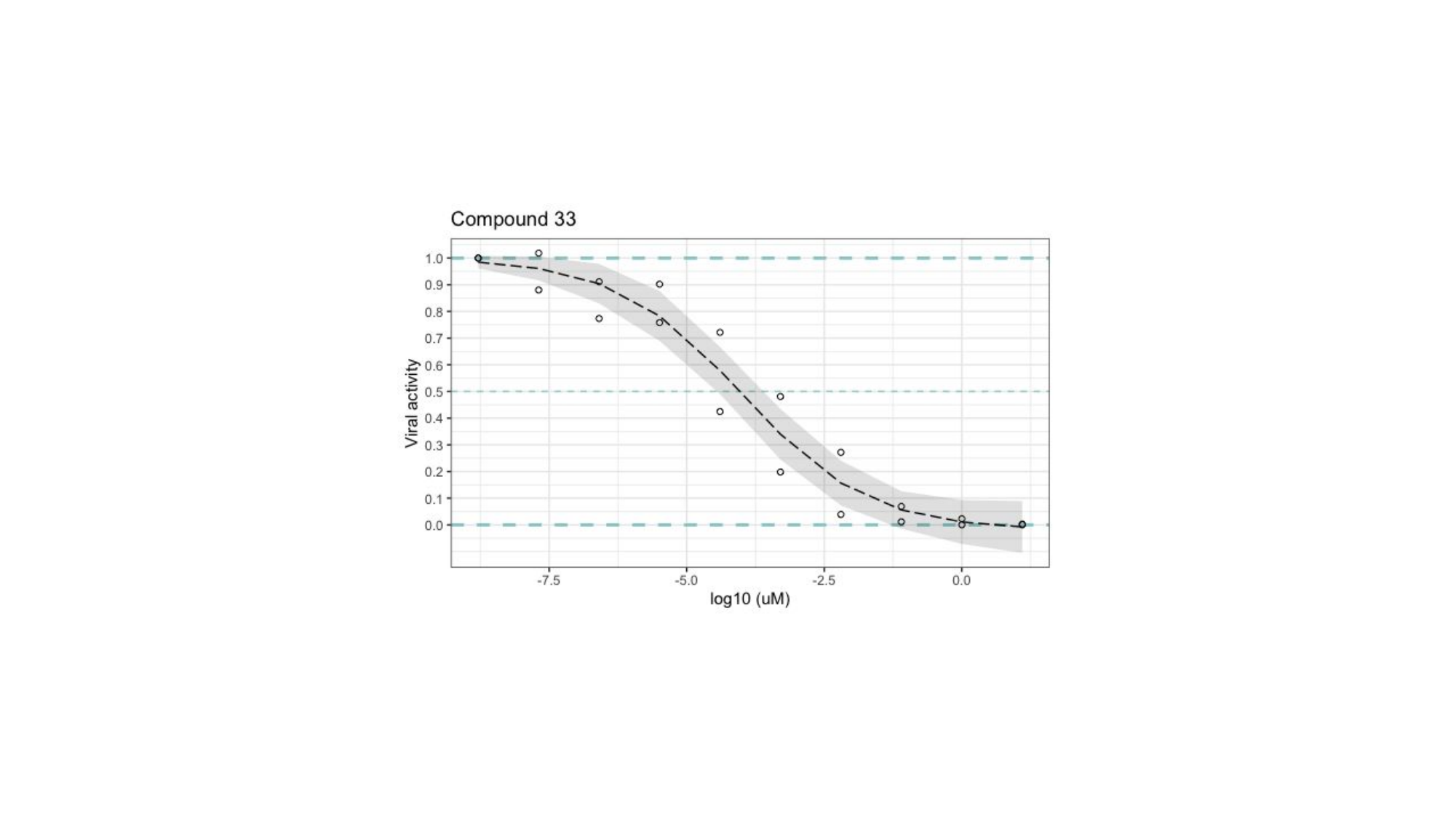

## Slide 26
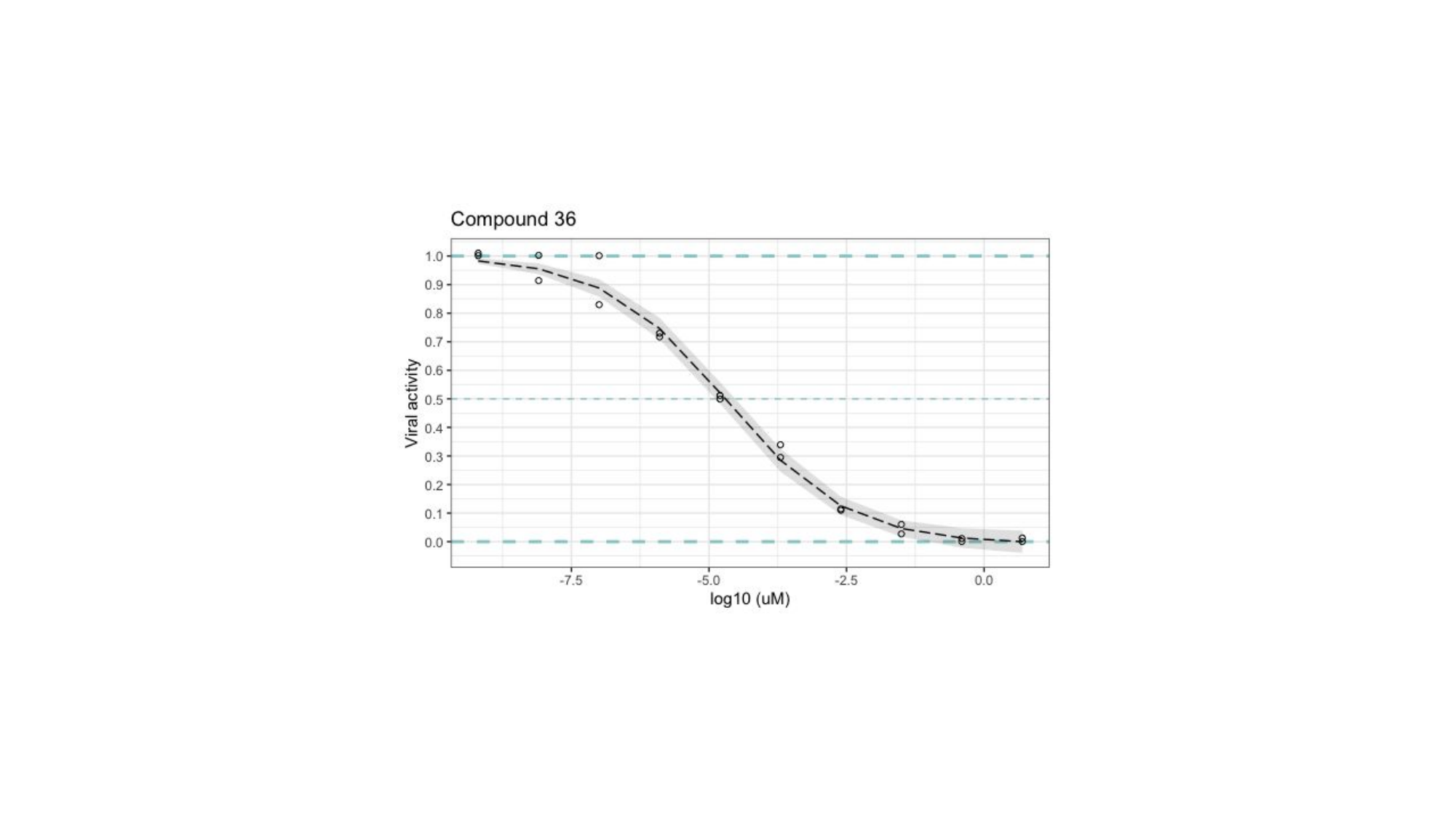

## Slide 27
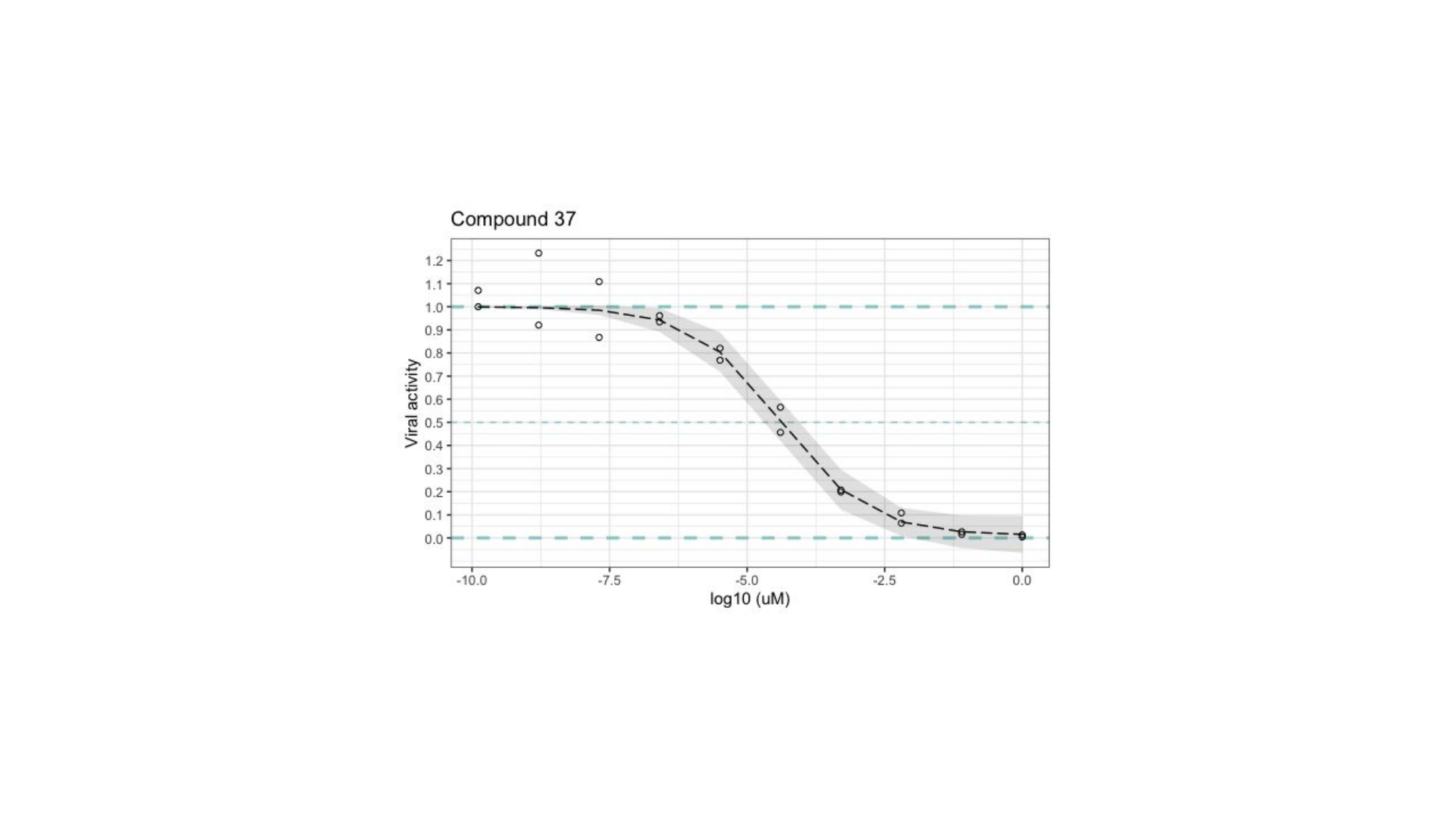

## Slide 28
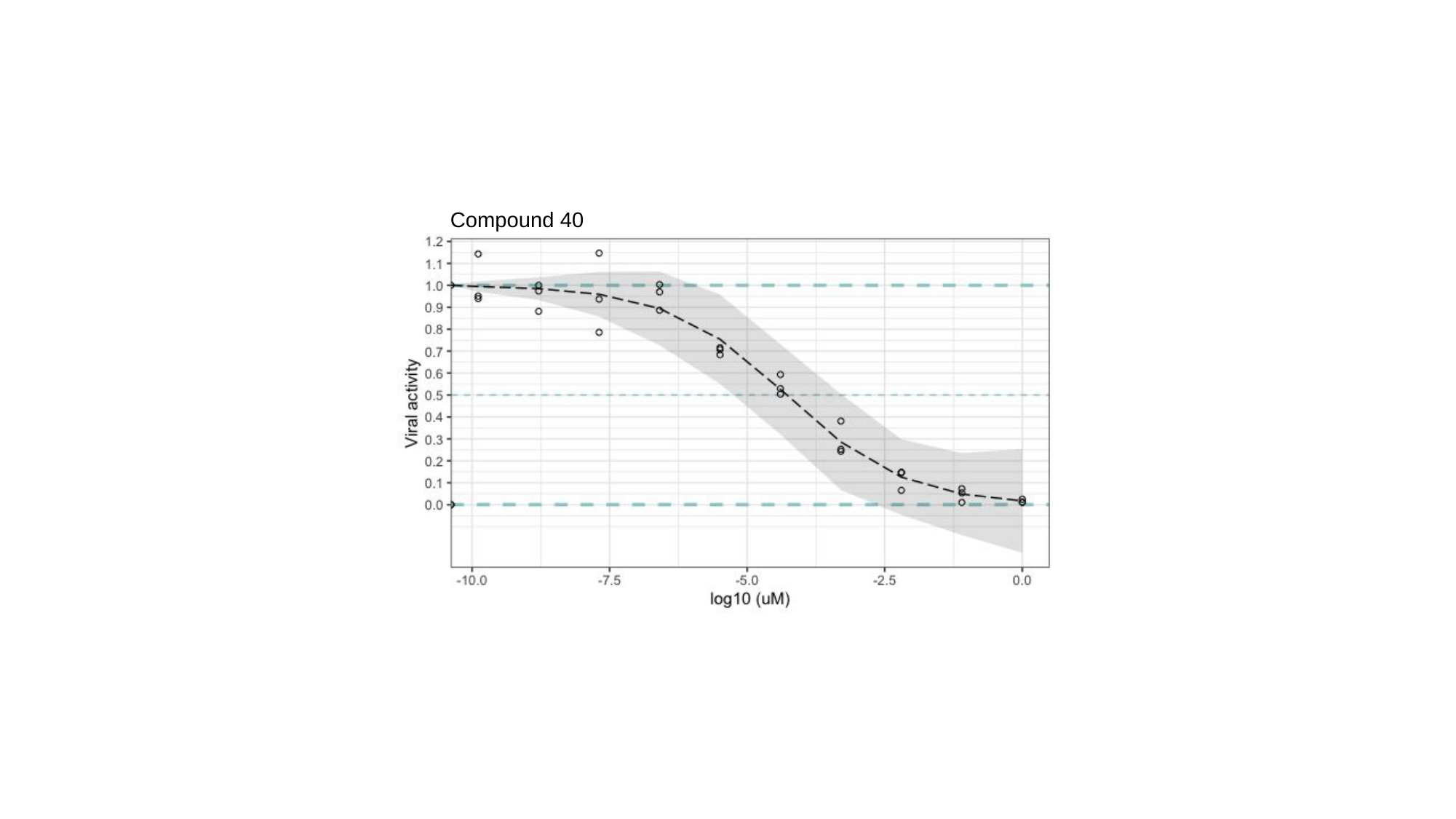

Compound 40

## Slide 29
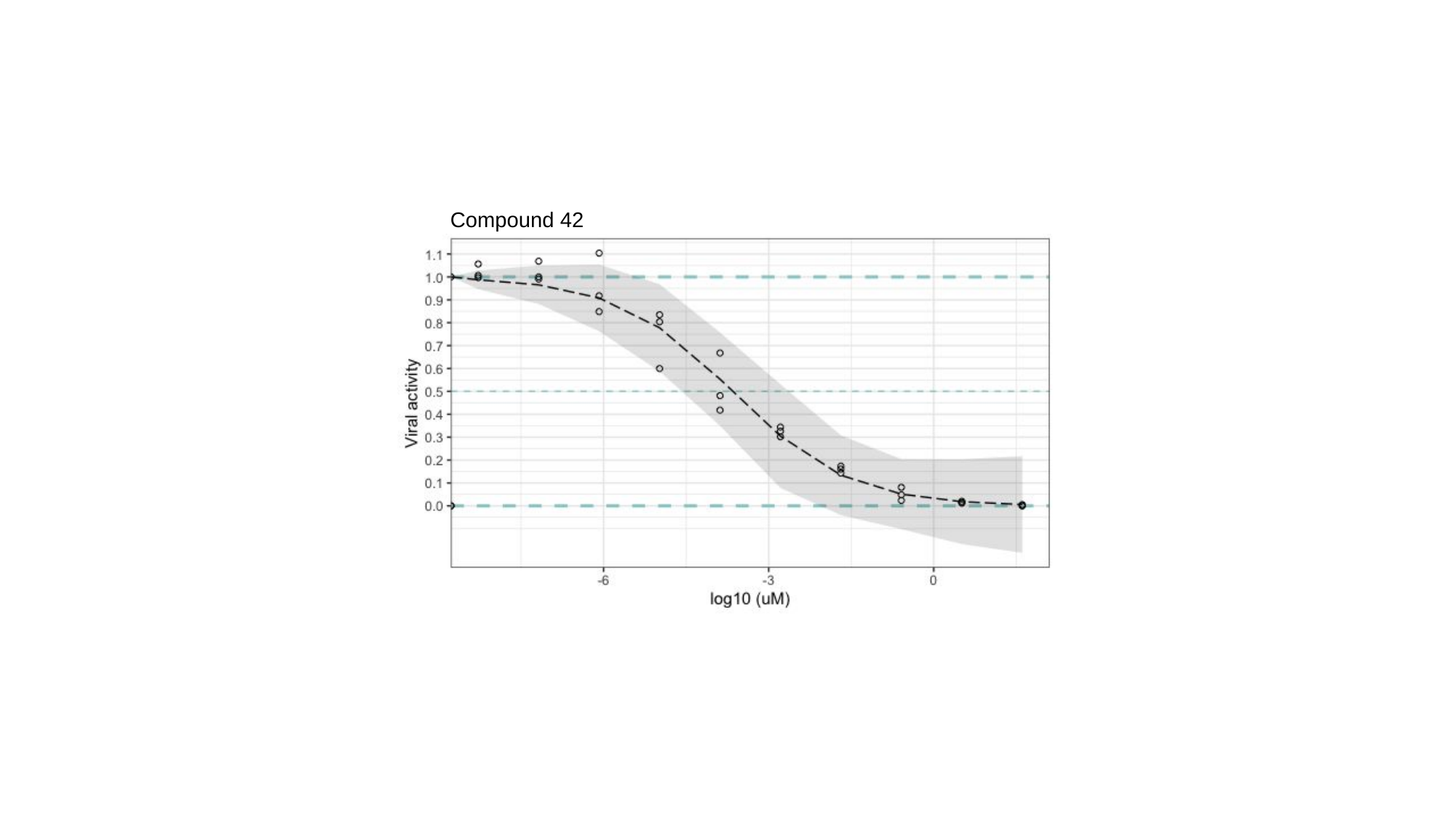

Compound 42
