# Supplementary material for: Expanding the toolbox of metabolically stable lipid prodrug strategies
Source: Front Pharmacol. 2023 Jan 6;13:1083284. doi: 10.3389/fphar.2022.1083284 (PMC9852841; doi:10.3389/fphar.2022.1083284)
Supplement: Supplementary file 2 [file DataSheet1.docx]

*Supporting Information*

Expanding the Toolbox of Metabolically Stable Lipid Prodrug Strategies

Kiran S. Toti^1’^, Nicole Pribut^1’^, Michael D’Erasmo^1^, Madhuri Dasari^1^, Savita K. Sharma^1^, Perry W. Bartsch^1^, Samantha L. Burton^1,2,3^, Hannah Gold^1^, Anatoliy Bushnev^1^, Cynthia A. Derdeyn^2,3,4,5^, Adriaan E. Basson^6^, Dennis C. Liotta^1^, Eric J. Miller^7^*.

^1^Department of Chemistry, Emory University, College of Arts & Sciences, Atlanta, GA 30322, USA

^2^Emory National Primate Research Center, Emory University, Atlanta, GA 30329, USA

^3^Emory Vaccine Center, Emory University, Atlanta, GA 30329, USA

^4^Department of Pathology & Laboratory Medicine, Emory University, School of Medicine, Atlanta, GA 30322

^5^Department of Laboratory Medicine & Pathology, University of Washington, Seattle, WA 98195 (current affiliation)

^6^HIV Pathogenesis Research Unit, Department of Molecular Medicine & Haematology, University of the Witwatersrand, Johannesburg, Gauteng 2193, South Africa

^7^Department of Pharmacology & Chemical Biology, Emory University, School of Medicine, Atlanta, GA 30322

^’^These authors contributed equally to this work and share first authorship.

***Correspondence:** Eric J. Miller, Ph.D. (orcid.org/0000-0003-3659-0105)
Corresponding Author
[ejmill2@emory.edu](mailto:ejmill2@emory.edu)

Abstract

Nucleoside- and nucleotide-based therapeutics are indispensable treatment options for patients suffering from malignant and viral diseases. These agents are most commonly administered to patients as prodrugs to maximize bioavailability and efficacy. While the literature provides a practical prodrug playbook to facilitate the delivery of nucleoside and nucleotide therapeutics, small context-dependent amendments to these popular prodrug strategies can drive dramatic improvements in pharmacokinetic (PK) profiles. Herein we offer a brief overview of current prodrug strategies, as well as a case study involving the fine-tuning of lipid prodrugs of acyclic nucleoside phosphonate tenofovir (TFV), an approved nucleotide HIV reverse transcriptase inhibitor (NtRTI) and the cornerstone of combination antiretroviral therapy (cART). Installation of novel lipid terminal motifs significantly reduced fatty acid hepatic ω-oxidation while maintaining potent antiviral activity. This work contributes important insights to the expanding repertoire of lipid prodrug strategies for the delivery and distribution of nucleoside and nucleotide therapeutics.

Table of Contents

HIV Pseudoviral Assay Concentration-Response Curves …………………………… 2–7

Human Liver Microsome (HLM) Stability Assay Details …………………………… 7–8

**HIV Pseudoviral Assay Concentration-Response Curves**


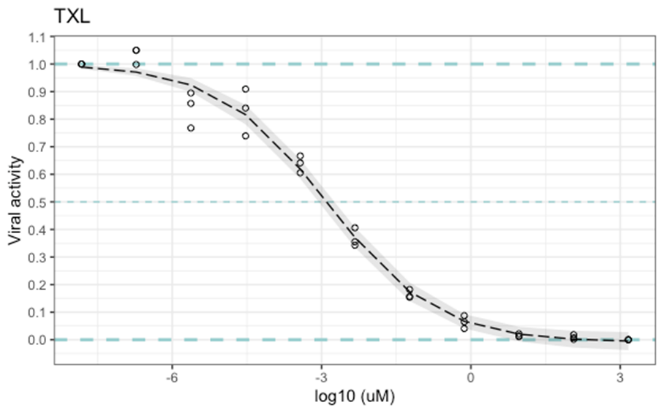


***Figure S1***: Concentration-response curve of TXL in HIV pseudoviral assays.


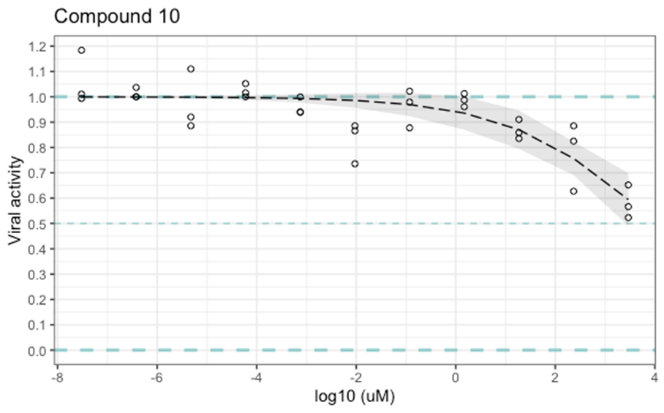


***Figure S2***: Concentration-response curve of prodrug **10** in HIV pseudoviral assays.


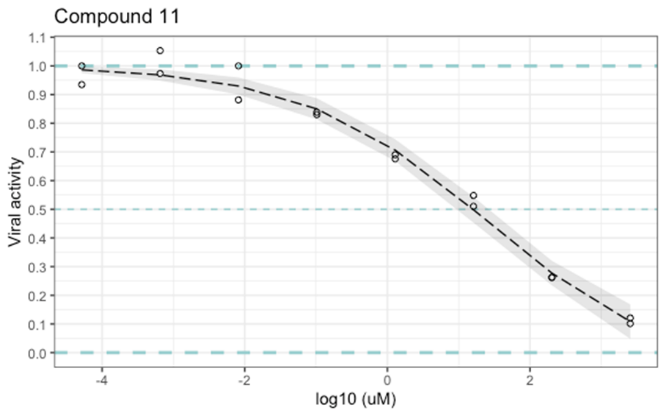


***Figure S3***: Concentration-response curve of prodrug **11** in HIV pseudoviral assays.


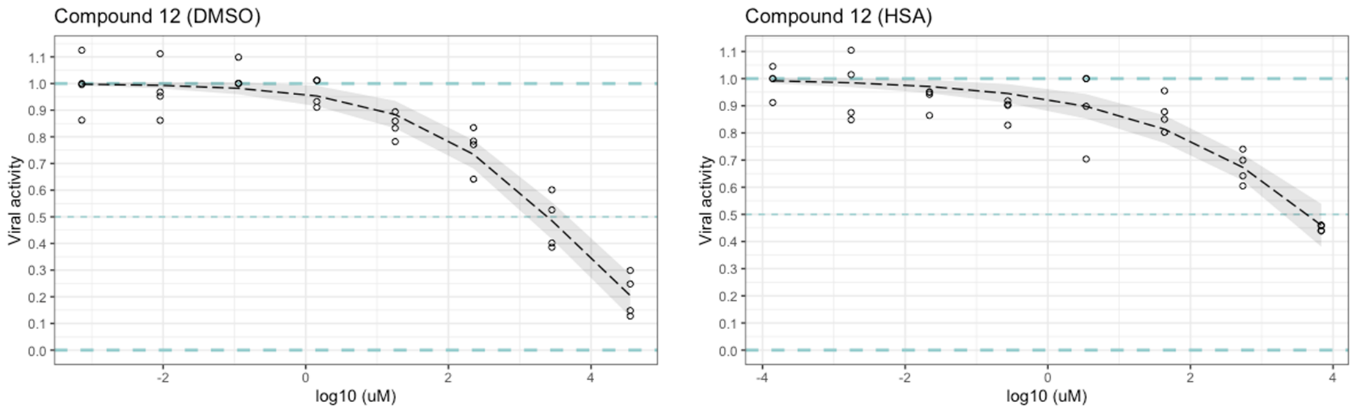


***Figure S4***: Concentration-response curve of prodrug **12** in HIV pseudoviral assays formulated with DMSO or human serum albumin (HSA).


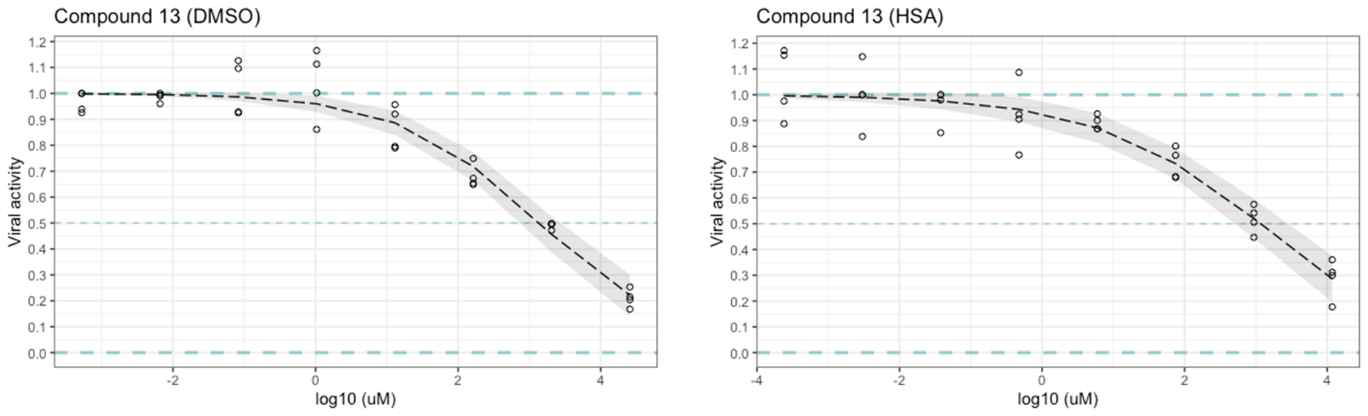


***Figure S5***: Concentration-response curve of prodrug **13** in HIV pseudoviral assays formulated with DMSO or human serum albumin (HSA).


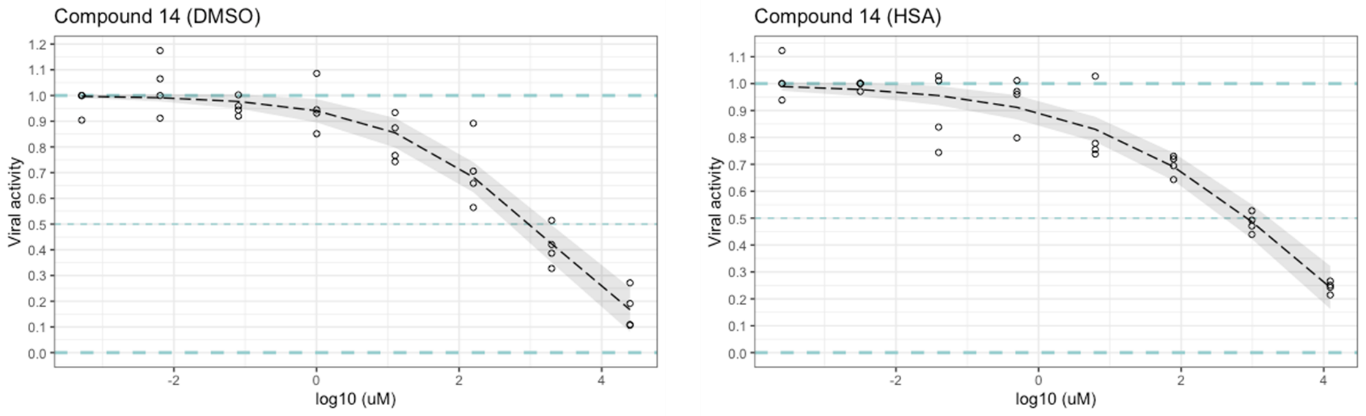


***Figure S6***: Concentration-response curve of prodrug **14** in HIV pseudoviral assays formulated with DMSO or human serum albumin (HSA).


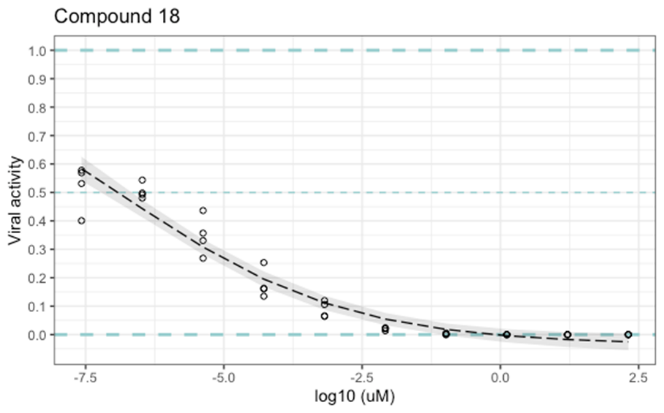


***Figure S7***: Concentration-response curve of prodrug **18** in HIV pseudoviral assays.


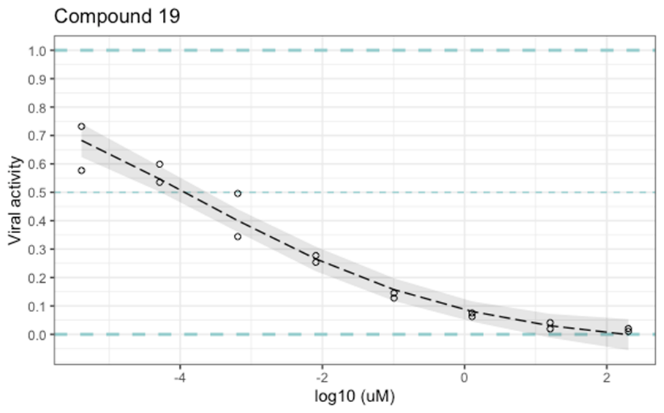


***Figure S8***: Concentration-response curve of prodrug **19** in HIV pseudoviral assays.


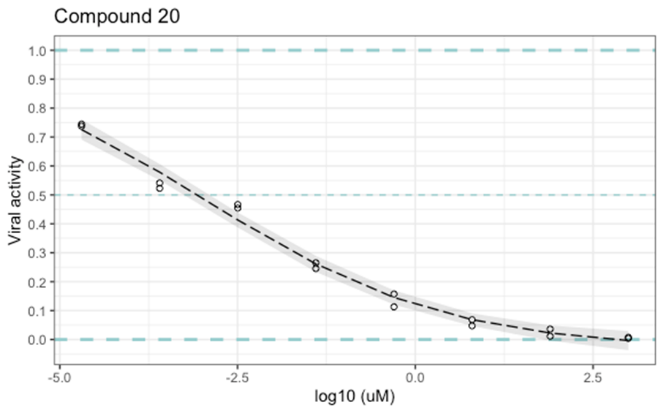


***Figure S9***: Concentration-response curve of prodrug **20** in HIV pseudoviral assays.


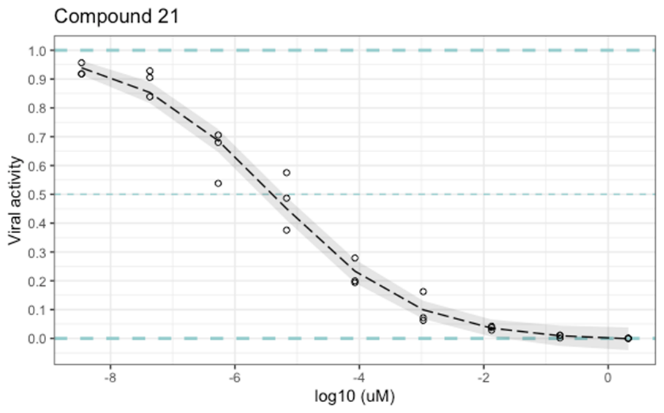


***Figure S10***: Concentration-response curve of prodrug **21** in HIV pseudoviral assays.


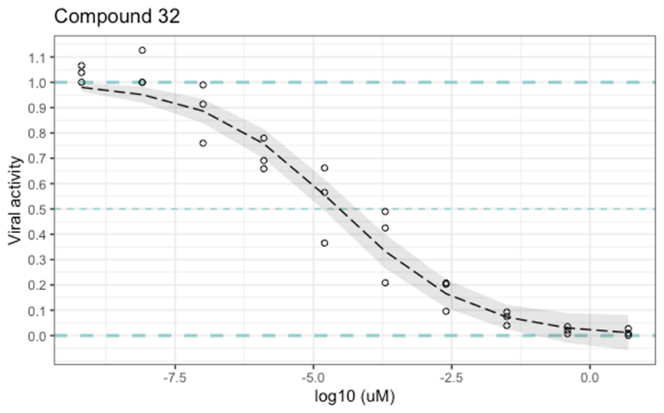


***Figure S11***: Concentration-response curve of prodrug **32** in HIV pseudoviral assays.


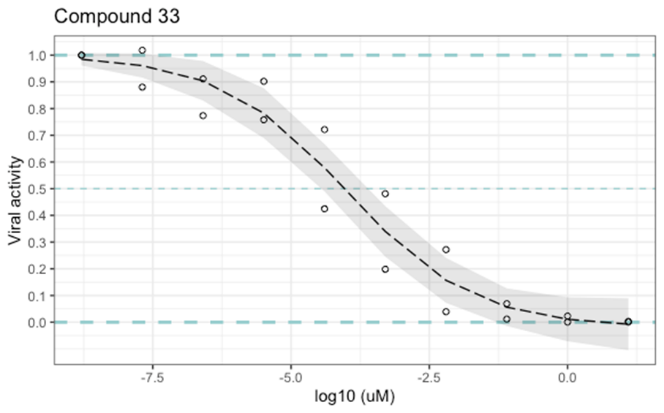


***Figure S12***: Concentration-response curve of prodrug **33** in HIV pseudoviral assays.


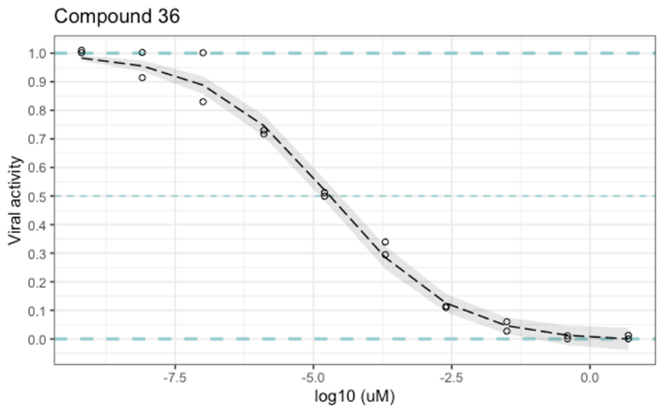


***Figure S13***: Concentration-response curve of prodrug **36** in HIV pseudoviral assays.


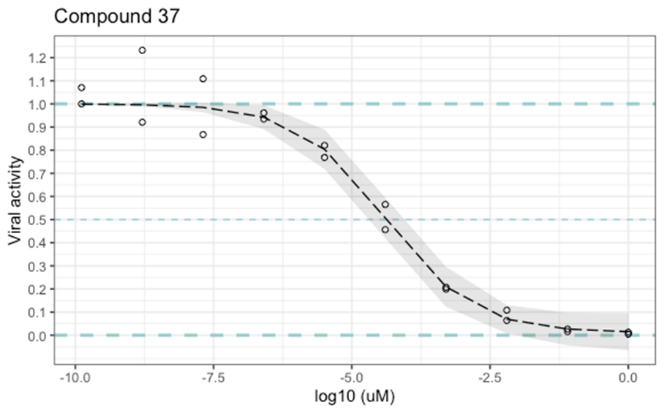


***Figure S14***: Concentration-response curve of prodrug **37** in HIV pseudoviral assays.


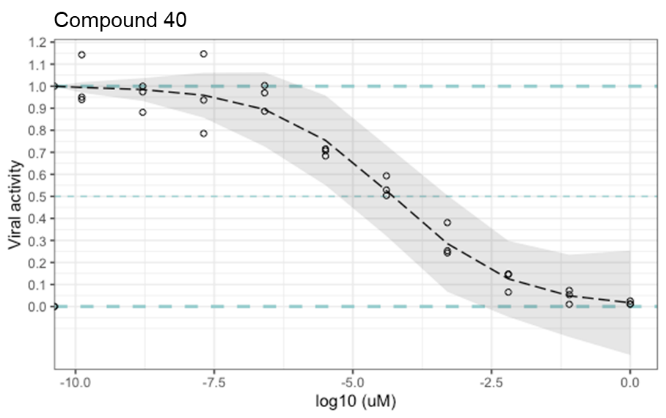


***Figure S15***: Concentration-response curve of prodrug **40** in HIV pseudoviral assays.


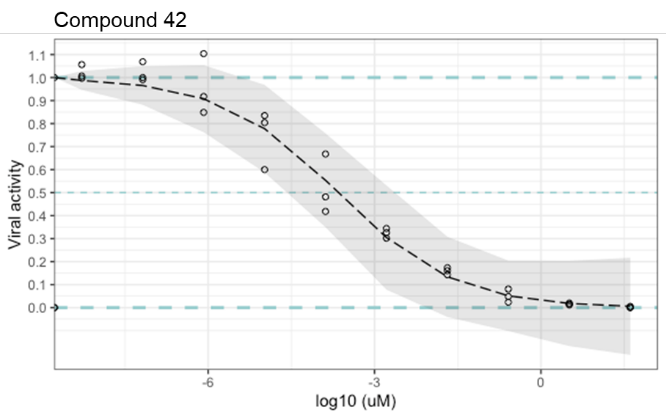


***Figure S16***: Concentration-response curve of prodrug **42** in HIV pseudoviral assays.

**Human Liver Microsome (HLM) Stability Assays**

Representative HLM Stability Assay Setup:

- Test Compound (TC): 928.4 μL Potassium Phosphate Buffer (100 mM) + 55 μL HLM + 110 μL NADPH (10 mM) + 6.6 μL TC stock solution (500 μM)
- Positive Control: 464.2 μL Potassium Phosphate Buffer (100 mM) + 27.5 μL HLM + 55 μL NADPH (10 mM) + 3.3 μL Verapamil (500 μM)
- Negative Control: 141.6 μL Potassium Phosphate Buffer (100 mM) + 7.5 μL HLM + 0.9 μL TC (500 μM)
- Quenching Mixture: 100 μL MeOH with ISTD (2 μM 7-ethoxy-*d*_5_ coumarin)
- Final Volume After Quenching: 200 μL (100 μL from reaction mixture + 100 μL quencher solution; ISTD final concentration of 1.0 μM)

| Compound ID | HLM t_1/2_ (min) | St. Dev. (min) | Cl_int_ (μL/min/mg) |
| --- | --- | --- | --- |
| TXL | 41.6 | 9.02 | 16.7 |
| 11 | 11.8 | 0.58 | 58.6 |
| 18 | 34.9 | 1.13 | 19.8 |
| 19 | 48.0 | 0.28 | 14.4 |
| 20 | 30.9 | 0.27 | 22.5 |
| 21 | 105 | 3.72 | 6.60 |
| 32 | >120 | - | <5.78 |
| 33 | >120 | - | <5.78 |
| 36 | >120 | - | <5.78 |
| 37 | >120 | - | <5.78 |
| 42 | >120 | - | <5.78 |
| 40 | >120 | - | <5.78 |

***Table S1***: Half-lives, standard deviation, and corresponding clearance values for all produgs evaluated for metabolic stability in HLM.

| Compound  ID | Precursor  Ion MS1 | Product  Ion MS2 | Fragmentor Voltage (V) | Collision  Energy (V) | Cell  Accelerator (V) | Polarity |
| --- | --- | --- | --- | --- | --- | --- |
| TXL | 570.4 | 270.1  206.1  176.0 | 200 | 36  44  64 | 4 | Positive |
| 11 | 572.4 | 199.2  176.1  87.2 | 182 | 21  61  29 | 4 | Positive |
| 18 | 570.4 | 270.1  206.1  176.1 | 212 | 33  45  57 | 4 | Positive |
| 19 | 570.4 | 283.3  73.2 | 172 | 17  29 | 4 | Positive |
| 20 | 570.4 | 176.1  87.2 | 192 | 61  29 | 4 | Positive |
| 21 | 584.4 | 288.1  270.1  176.1 | 170 | 33  37  50 | 4 | Positive |
| 32 | 606.5 | 288.2  270.2 | 110 | 37 | 4 | Positive |
| 33 | 620.3 | 288.2  270.2 | 100 | 37 | 4 | Positive |
| 36 | 610.3 | 288.3  270.2 | 110 | 37 | 4 | Positive |
| 37 | 624.4 | 288.2  270.2  206.2 | 100 | 37  37  50 | 4 | Positive |
| 42 | 702.3 | 288.1  270.1 | 144 | 41 | 4 | Positive |
| 40 | 698.3 | 288.1  270.1 | 124 | 41 | 4 | Positive |
| Verapamil | 455.1 | 303.2  165.1 | 126 | 24  28 | 4 | Positive |
| *7-Ethoxy-d_5_ coumarin* (ISTD) | 196.1 | 164.0 | 116 | 17 | 4 | Positive |

***Table S2***: LC-MS/MS scan parameters and corresponding transitions in MRM mode for all compounds evaluated for metabolic stability in HLM.
